# Physical activity monitors to enhance amount of physical activity in older adults – a systematic review and meta-analysis

**DOI:** 10.1186/s11556-019-0213-6

**Published:** 2019-05-04

**Authors:** Rasmus Tolstrup Larsen, Jan Christensen, Carsten Bogh Juhl, Henning Boje Andersen, Henning Langberg

**Affiliations:** 1 CopenRehab, Department of Public Health, Section of Social Medicine, University of Copenhagen, Gothersgade 160, 3rd floor, 1123 Copenhagen K, Denmark; 2 Department of Occupational- and Physiotherapy, Copenhagen University Hospital, Copenhagen, Denmark; 3 Technical University of Denmark, DTU Management Engineering Institute, Diplomvej 372 office 226, 2800 Lyngby, Denmark; 4 Research Unit of Musculoskeletal Function and Physiotherapy, Institute of Sports Science and Clinical Biomechanics, Faculty of Health Sciences, University of Southern Denmark, Odense, Denmark; 5 Department of Physiotherapy and Occupational Therapy, Copenhagen University Hospital, Herlev and Gentofte, Denmark; 6 National Centre for Rehabilitation and Palliative Care, University of Southern Denmark and Odense University Hospital, Odense, Denmark

**Keywords:** Aging, Physical activity, Older adults, Physical activity monitors, Technology, Motivation, Systematic review, Meta-analysis, Walking, Moderate to vigorous physical activity

## Abstract

**Background:**

The body of evidence related to the effect of physical activity monitor-based interventions has grown over the recent years. However, the effect of physical activity monitor-based interventions in older adults remains unclear and should be systematically reviewed.

**Objective:**

The objective of this systematic review was to estimate the effect of physical activity monitor-based interventions on physical activity behavior in participants aged 65 and above. Subsequently we explored the effect on body mass index, physical capacity, and health-related quality of life and finally the impact of patient- and intervention characteristics.

**Methods:**

Searches in MEDLINE, EMBASE, SPORTDiscus, CINAHL, and CENTRAL were performed on April 26, 2018. No publication date filters were applied. References of eligible studies were scrutinized and relevant journals were hand-searched. Randomized controlled trials and randomized cross-over trials investigating the effect of a physical activity monitor-based intervention on physical activity were included. Studies were included if the mean age of the participants was above 65 years, and participants could walk independently with or without walking aids. The Cochrane handbook was used as a template for extracting data and the RoB 2.0 tool was used to assess risk of bias. Random-effects meta-analysis using Hedges g, were used to pool the study results. The main outcome of this study was physical activity.

**Results:**

Twenty-one studies with 2783 participants were included. The median participant age in the studies was 70.5 years, the median percentage of male participants was 42%, and the median baseline daily step count was 5268. Physical activity monitor-based interventions had a moderate effect (SMD = 0.54, 95% CI: 0.34 to 0.73) compared to control interventions, corresponding to an average increase of 1297 steps per day in the intervention groups. No impact of patient and intervention characteristics on the effect estimates were found.

**Short conclusion:**

Low quality of evidence was found for a moderate effect of physical activity monitor-based interventions on physical activity compared with control interventions. More studies with higher research methodology standards are required.

**PROSPERO registration:**

CRD42018083648.

**Electronic supplementary material:**

The online version of this article (10.1186/s11556-019-0213-6) contains supplementary material, which is available to authorized users.

## Background

Physical inactivity is a growing worldwide problem and it has been reported to cause 9% of all premature death [1]. The amount of daily physical activity (PA) decreases with age [2–5] and one in eight European adults age 55 or older never or hardly ever, engage in moderate to vigorous PA (MVPA) [6]. Functional decline is expected and unavoidable in older adults, but regular exercise can minimize the physiological effects of an otherwise sedentary lifestyle and thus increase life expectancy by improving function of daily living and by slowing progression of disease and disability [7].

An older systematic review reported that physical activity monitor (PAM)-based interventions significantly enhanced the amount of PA with an average of 2491 steps per day, compared to the control group interventions, in adults [8]. Among older adults, the use of PAMs has been reported to be feasible [9, 10] and several recently published randomized controlled trials (RCTs) report promising results [11–16]. However, these studies differ with respect to sample characteristics, intervention length and setting, which might have resulted in the differences in the reported effect sizes between studies [11–16].

The body of evidence related to the effect of PAM-based interventions has grown over the recent years. However, the effect of PAM-based interventions in older adults remains unclear and should be systematically reviewed. Further, patient and intervention characteristics should be explored to understand their impact on PA levels. This information may be used to inform future research and provide guidance to clinical decision-makers considering the use PAMs in PA programs.

### Objective

The objective of this systematic review and meta-analysis was to estimate the effect of PAM-based interventions on amount of PA (e.g. daily step count) in participants aged 65 and above. Subsequently we aimed to explore the effect on time spent sedentary, MVPA time, physical capacity (e.g. measured by a cardiopulmonary exercise test or as a walking test), body mass index (BMI), and health-related quality of life (HRQoL) (e.g. by questionnaires). Finally, we sought to investigate the impact of participant- (e.g. diagnoses, age and sex distribution), intervention- (e.g. intervention length, type of PA measure and feedback frequency) and study (e.g. risk of bias) characteristics on the results.

## Methods

### Protocol and registration

This systematic review and meta-analysis is detailed according to the recommendations of the Cochrane Handbook [17] and it is reported according to the PRISMA statement. The method is described in the published review protocol as well as in the PROSPERO registration (CRD42018083648) [18, 19]. Unless otherwise stated, the methods used and reported in this systematic review followed the review protocol.

### Eligibility criteria

We included RCTs and randomized cross-over trials comparing any PAM-based intervention where the participants of the intervention group received any kind of feedback on their physical activity level measured by PAMs, and where the control intervention did not receive feedback from the PAMs. The mean age of the participants should be above 65 years, and participants should be able to walk independently with or without walking aids.

The primary outcome was PA. If more than one type of PA measure were reported, we extracted or calculated it in the following order: daily number of steps, daily number of meters walked, daily amount of energy expenditure (calories), daily metabolic equivalent of task (minutes or hours) and finally, if no objective measure was available, self-reported PA. The secondary outcomes included:

- Time spent as sedentary (measured objectively by PAMs)
- Time spent in MVPA (measured objectively by PAMs or secondly as self-reported behavior)
- Physical capacity (measured by a cardiopulmonary exercise test or secondly as a walking test)
- BMI
- Self- reported HRQoL determined by questionnaires.

End-point scores were used to calculate treatment effects. To avoid unit-of-analysis error with cross-over trials, the outcome was extracted at baseline and when the first period ended, as recommended in the Cochrane Handbook chapter 16.4.5 [20]. Reported adverse events or withdrawals due to illness were extracted if possible.

### Information sources

Preliminary searches and identification of relevant papers were performed to identify relevant search terms and subject headings. The final systematic search for eligible studies in MEDLINE, EMBASE, SPORTDiscus, CINAHL, and CENTRAL was performed on April 26, 2018. Additional studies that met the inclusion criteria were obtained through an independent review of article references by two reviewers (RTL and JC). The Clinicatrials.gov database was searched on February 13th 2018 to locate ongoing relevant studies.

### Search

The search string consisted of a combination of relevant keywords and subject headings for: PAMs, older adults, and randomized studies and can be found in the study protocol [19]. No restrictions on language or publication-time were applied. The authors of unobtainable studies or studies with missing data were contacted to obtain missing information.

#### Study selection, data items and data collection process

Citations was imported into the technology platform, *Covidence.* Two authors (RTL and JC) screened the titles and abstracts independently and assessed full-text reports. Any inconsistencies between authors was discussed and rectified in consultation with a third author (CJ). Data extraction was performed independently by two authors (RTL and JC).

#### Risk of bias in individual studies

Two review authors (RTL and JC) independently assessed study quality using the Risk of Bias 2.0 tool [21] on study outcome level. Disagreement was solved by discussion with a third reviewer (CJ).

#### Summary measures

Treatment effects, on continuous data, were expressed as standardized mean differences (SMD) with 95% confidence intervals. The SMD was translated back to a mean difference in steps for the primary outcome, MVPA time, meters on 6MWT for physical capacity and BMI (kg/m^2^) respectively, using the method described by Bliddal and Christensen [22]. The SDs used for translating the SMDs were extracted for each outcome from the intervention group from largest study with the lowest risk of bias, in which objectively measured values were favored. The SDs used were 2402 steps per day [23], 16.2 min of daily MVPA [14], 80 m on a 6MWT [24] and 4.8 kg/m^2^ on BMI [25]. When reported in text or study flow diagrams, adverse event and participant withdrawal rates were extracted and expressed as relative risks with 95% confidence intervals. If a study reported zero adverse events, the Der-Simonian & Laird method was used and 0.5 was added as a value to enable random effects meta-analysis [26].

#### Synthesis of results

The effect size was calculated using a random-effects model adjusting to Hedges’ *g,* using end-point scores only. In studies where no continuous data were available for the outcomes, we used dichotomous data and converted the odds ratios and the standard errors (log ES) into the standard mean difference using the Chinn et al. approach described in chapter 9.4.6 of the Cochrane Handbook [27]. An alpha level of 0.05 was considered statistically significant. Stata/IC 15.1 for Mac (64-bit Intel), Copyright 1985–2017 StataCorp LLC was used for all statistical analyses.

#### Unit of analysis issues

One study had two relevant intervention groups [11]. The intervention groups of the study were included as two separate comparisons and the control group from the study was separated according to guideline in the Cochrane Handbook chapter 16.5.4 [28].

#### Additional analyses

The heterogeneity of the extracted results was examined using the Cochrane Q test and quantified with I^2^ statistic. We performed subgroup analyses to explore the impact of characteristics of participants and intervention and stratified the effect size on the following nominal variables: type of PAM (accelerometer versus pedometer), diagnoses of participants (none, cardiac patients, COPD or osteoarthritis), feedback frequency (daily, weekly or monthly) and overall risk of bias (low, some concerns and high). We conducted three explorative subgroup analyses: one analysis on control intervention content (advice group, goal setting, maintain usual PA, other training, rehabilitation program, and usual care), one analysis on grouping the interventions into types (gamification, incremental goals, monthly feedback and reinforcement, ongoing counseling and pre-counseling), and one analysis on active control interventions versus non-active control interventions (maintain usual PA or no intervention).

We chose to investigate how the method of physical activity reporting (i.e., objective measurement, self-report, interview) affected the results. This sensitivity analysis was deemed more informative than the protocolled sensitivity analysis on mean differences in daily number of steps, daily number of meters walked, daily amount of energy expenditure measured as calories, daily metabolic equivalent of task, and self-reported physical activity.

Publication bias were assessed by Eggers test. If small study bias was present, the Duval and Tweedie nonparametric “trim and fill” analysis was conducted adjusting the effect size [29, 30].

We performed univariate meta-regressions on continuous data on the following variables:

Age (years), sex distribution (percent), number (or percent) of participants with walking aids, intervention length (weeks), baseline PA (steps), and BMI.

## Results

### Study selection

**Fig. 1:**
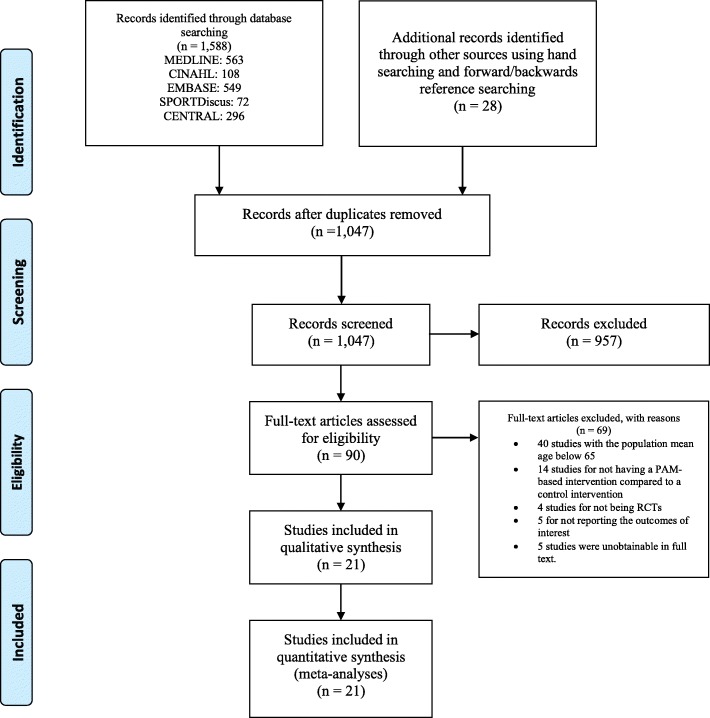
PRISMA flow diagram illustrating the inclusion process

Twenty-one studies were included in the review [11–16, 23–25, 31–42]. We identified one ongoing trial (Clinicaltrials.gov Identifier: NCT03086850), but we did not include this study as it was in the participant recruitment phase. Citations and reasons for exclusion from full text screening are listed in the Additional file 1: Table S3. The study selection process is illustrated in Fig. 1. A summary of the included studies is listed in Table 1. Characteristics of the 21 included studies (22 comparisons, 2783 participants) are listed in Additional file 1: Table S2.

**Table 1:** Summary of the characteristics of the included studies. Citations of studies that reported results on domains are listed after the domain

Methods	Number of studies (%)
RCT with parallel group design [11–16, 23–25, 31, 32, 34–42]	20 (95%)
RCT with cross over design [33]	1 (5%)
Setting	Number of studies (%)
Europe [12, 13, 23, 24, 31, 32, 35, 38]	8 (38%)
Australia and New Zealand [14, 16, 25, 37]	4 (19%)
Asia [15, 36, 40, 41]	4 (19%)
North America [11, 33, 34, 39, 42]	5 (24%)
Participant diagnoses	Number of studies (%)
Osteoarthritis [23]	1 (5%)
COPD [12, 31, 36]	3 (14%)
Cardiac patients [16, 40]	2 (10%)
None [11, 13–15, 24, 25, 32–35, 37–39, 41, 42]	15 (71%)
Participant characteristics	Median (range)
Median age in studies (k = 21)	70.5 (65 to 81.5)
Median body mass index in studies [11, 12, 14, 15, 23, 25, 31, 35, 36, 39, 40, 42]	27.9 (21.1 to 31.82)
Median percentage of male participants in studies [11–16, 23–25, 31, 33–36, 38–42]	42 (0 to 88)
Median percentage of married participants [13, 16, 24, 25, 32, 35, 39]	61.4 (39 to 80.5)
Median baseline daily step count [11–13, 15, 23, 31, 33–35, 39, 41]	5268 (2420 to 7697)
Intervention	Median (range)
Length median weeks (k = 21)	12 (4 to 52)
Physical activity monitor	Number of studies (%)
Accelerometer [12, 14, 32, 36, 41]	5 (24%)
Pedometer [11, 13, 15, 16, 23–25, 31, 33–35, 37–40, 42]	16 (76%)
Frequency of feedback	Number of studies (%)
Daily [11–13, 15, 16, 23–25, 32, 33, 35, 37–42]	17 (81%)
Weekly [14, 31, 33]	3 (14%)
Monthly [36]	1 (5%)
Outcomes	Number of studies (%)
Reported results on physical activity [11–16, 23–25, 31–36, 38–42]	20 (95%)
Reported results on sedentary time [13]	1 (5%)
Reported results on MVPA time [14, 24, 25, 31, 35, 41, 42]	7 (33%)
Reported results on physical capacity [24, 36, 37, 41]	4 (19%)
Reported results on health-related quality of life [13, 24, 25, 31, 36]	5 (24%)
Reported results on body mass index [25, 36]	3 (14%)
Reported results on adverse events [13, 14, 16, 24, 31, 32, 35–37, 40]	10 (48%)

*RCT* Randomized Controlled Trial, *COPD* Chronic Obstructive Pulmonary Disease, *MVPA* Moderate to Vigorous Physical Activity, *k* number of studies. The reported median of mean values are unweighted in relation to study size or reporting precision

### Study characteristics

#### Risk of bias within studies

**Fig. 2:**
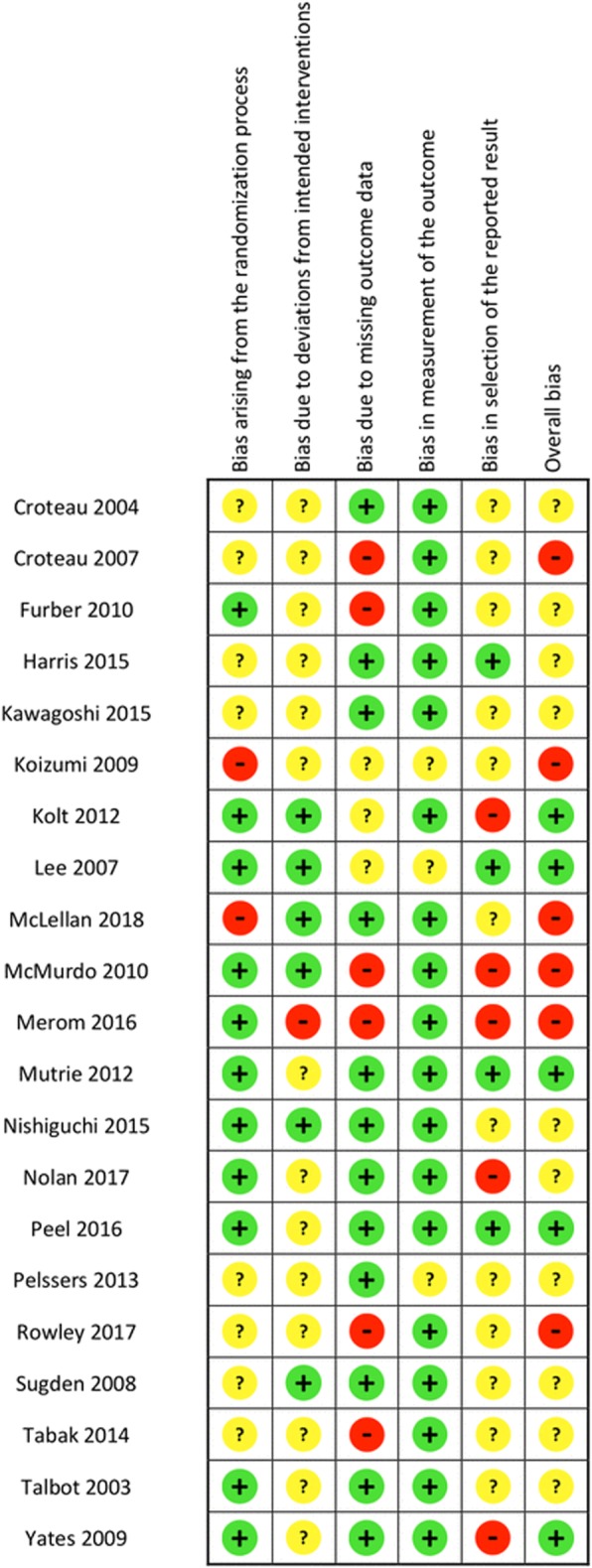
Risk of bias summary: review authors’ judgements about each risk of bias item for each included study. +: Low risk of bias,?:Some concerns, %: High risk of bias

**Fig. 3:**
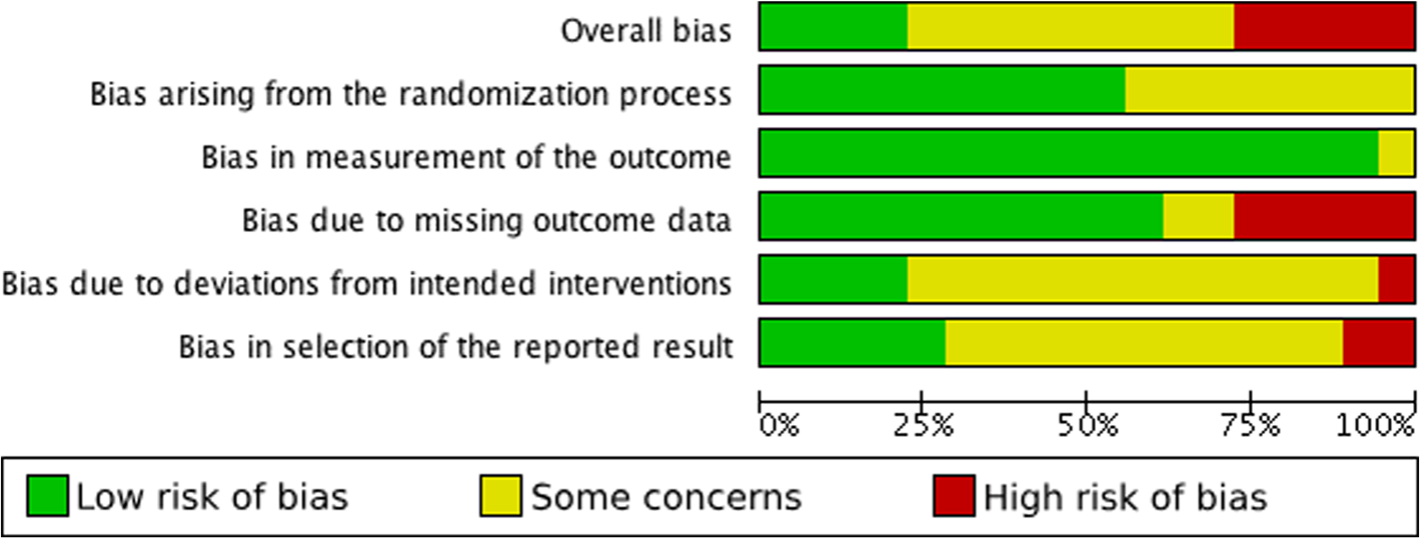
Risk of bias graph: review authors’ judgements about each risk of bias item presented as percentages across all included studies

The risk of bias summary and review authors’ judgements about each risk of bias item are presented in Fig. 2. Figure 3 illustrates the risk of bias as percentages across all included studies for each risk of bias item. Overall, five studies were considered as having a low risk of bias [13, 14, 23, 25, 40], 10 studies were considered as having some concerns [12, 15, 16, 24, 31, 34–36, 38, 39], and six studies were considered as having a high risk of bias [11, 12, 32, 33, 37, 41]. Judgements and support for judgement about each item is presented for all studies in characteristics of studies in Additional file 1.

In two studies the risk of bias assessment differed between outcomes. Kolt et al. and Nolan et al. were assessed to have high risk of selective outcome reporting, on self-reported HRQoL (SF-36) [25, 31].

### Synthesis of the results and effect of the interventions

**Fig. 4:**
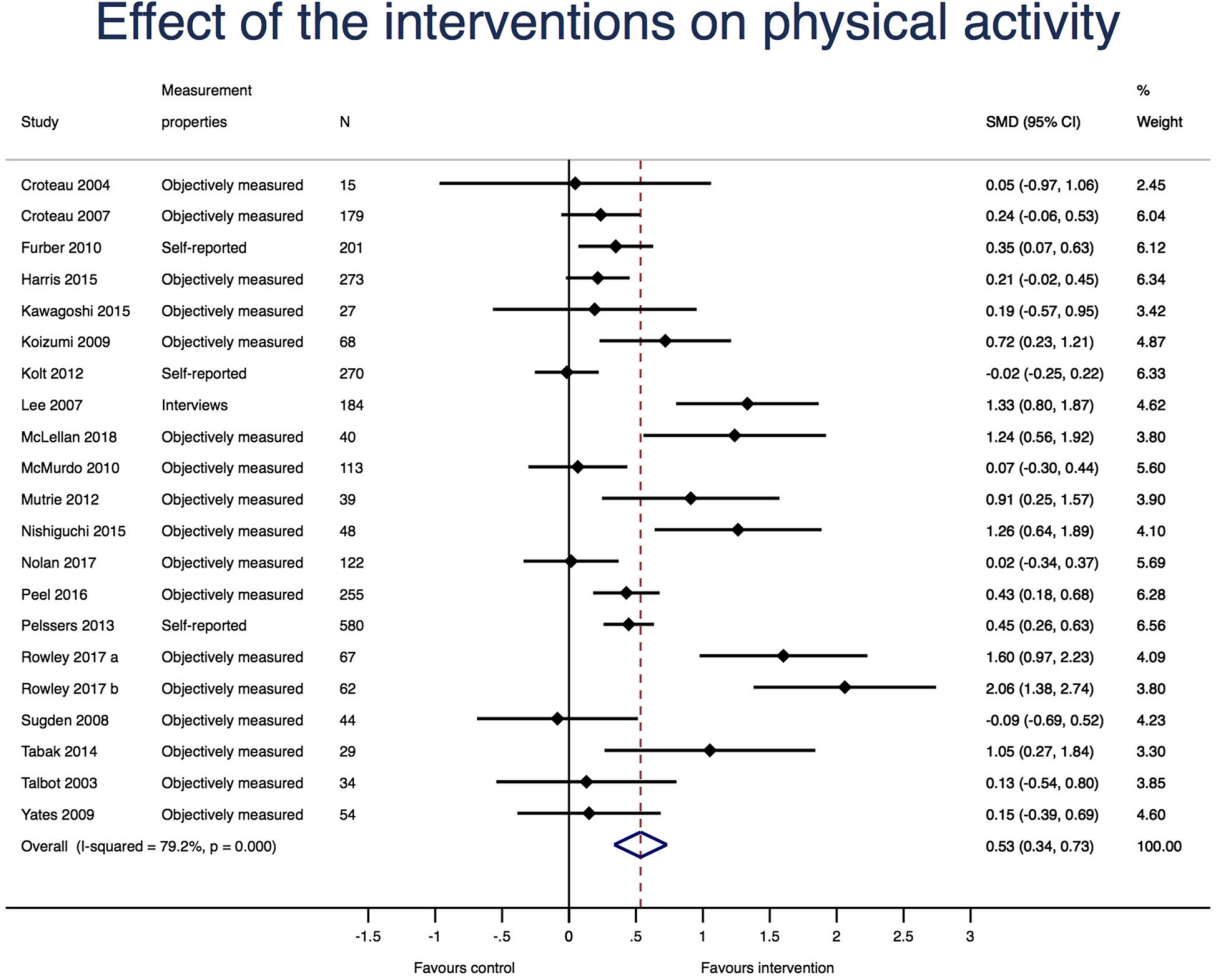
Random effects meta-analysis with effect of the interventions on physical activity using Hedges g. N: Number of participants; SMD; standardized mean difference. For each study, the diamond represents the standardized mean difference of the intervention effect with the horizontal line representing 95% confidence intervals. The large diamonds represent the pooled standardized mean difference between the intervention groups and the control groups

Twenty studies (21 study comparisons and 2704 participants) evaluated the effect of PAM on PA. The random effects meta-analysis is illustrated in Fig. 4. The overall SMD was 0.54, (95% CI: 0.34 to 0.73), I^2^ = 79.2%, *p* < 0.001, favoring the PAM interventions. When using a SD of 2402 steps per day, this corresponds to a weighted mean difference of 1297 (95% CI: 817 to 1753) favoring the intervention groups [23].

### Secondary outcomes

Only one study (35 participants) reported the effect of the intervention on time spent sedentary [13]. The SMD of this study was calculated to be − 0.40 (95% CI: -1.07 to 0.27), favoring the PAM intervention. The difference in weekly sedentary time was 44.0 min (95% CI: 37.1 to 50.9) with the control group being most sedentary.

A total of eight studies (1686 participants) reported data on effect of the interventions on MVPA time. The overall SMD was 0.34 (95% CI: 0.15 to 0.52), I^2^ = 65.8%, *p* = 0.005, favoring the PAM interventions. When using a SD of 16.2 of daily MVPA, this corresponds to a weighted mean difference of 5.5 min per day (95% CI: 2.4 to 8.4) with more MVPA in the intervention groups [14].

A total of four studies (754 participants) reported the effect of the intervention on physical capacity. The overall SMD was 0.19 (95% CI: -0.10 to 0.48), I^2^ = 48.8%, *p* = 0.118, favoring the PAM intervention. When using a SD of 80 m, this corresponds to a weighted mean difference on 15 m (95% CI: -8 to 38) with more meters walked on a 6MWT in the intervention groups [24].

A total of three studies (570 participants) reported data for effect of the interventions on BMI. The overall SMD was 0.15, (95% CI: -0.01 to 0.31), I^2^ = 0%, *p* = 0.752, favoring the control intervention. When using a SD of 4.8 kg/m^2^, this corresponds to a mean difference on 0.72 kg/m^2^ (95% CI: -0.05 to 1.50) with the control groups having the lowest BMI [25].

A total of five studies (1038 participants) reported data for effect of the interventions on HRQoL. The overall SMD was 0.01, (95% CI: -0.12 to 0.14), I^2^ = 0.0%, *p* = 0.541, favoring the PAM interventions.

**Fig. 5:**
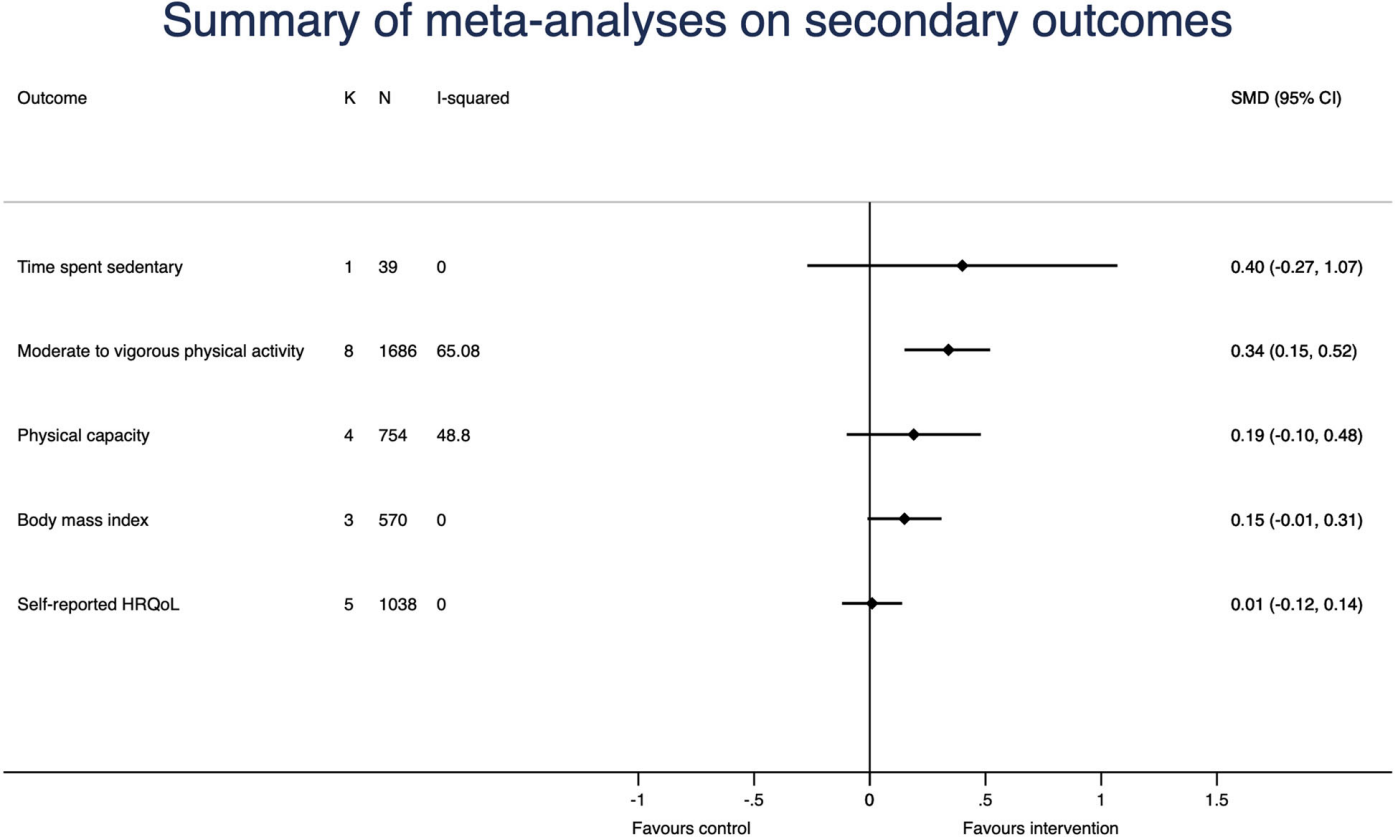
Summary of random effects meta-analyses with effect of the interventions on secondary outcomes. K: number of studies; N: Number of participants; SMD: standardized mean difference; HRQoL: health-related quality of life. For each analysis, the diamond represents the standardized mean difference of the pooled intervention effect with the horizontal line representing 95% confidence intervals

A summary of the analyses on the secondary outcomes are illustrated in Fig. 5.

#### Meeting the study specific recommended level of physical activity

No studies reported data on this.

### Additional analyses

Subgroup analyses on the type of PAM, diagnoses, feedback frequency, risk of bias judgement and type of PA measure on the effect of the intervention on PA (Additional file 1: Figure S6), MVPA time (Additional file 1: Figure S7), physical capacity (Additional file 1: Figure S8), BMI (Additional file 1: Figure S9), and HRQoL (Additional file 1: Figure S10) are presented in the Additional file 1. No significant differences in the subgroup analyses were observed for any outcomes.

Additional file 1: Table S3 reports data from sensitivity-analyses (univariate meta-regressions) on how the SMD from all outcomes sere affected by the following variables: age in years, sex distribution in percent male, percent of participants with walking aids, intervention length in weeks, baseline PA measured in steps per day, BMI in kg/m^2^. None of the above-mentioned variables were significantly correlated with the effect size for any outcomes, nor did any variable reduce Tau^2^ statistic. There were insufficient observations to analyze the correlation between effect size and percent of participants with walking aids for all outcomes.

Egger’s test showed significant small study bias for the effect on PA (*p* = 0.036), indicating that the analyzes are overestimating the effect on PA (Additional file 1: Figure S11). The bias adjusted (trimmed and filled) analysis with random effects revealed an adjusted SMD on 0.37, (95% CI: 0.15 to 0.59) after filling the analysis with three fictive studies. Analyzing the effect on time spent in MVPA, physical capacity, BMI, and HRQoL, no small study bias was found using Egger’s test.

### Adverse events

A total of 11 studies (1927 participants) reported data for adverse events. The overall relative risk for adverse events was 0.91, (95% CI: 0.66 to 1.25), I^2^ = 0.0% *p* = 0.942, with more adverse events and withdrawals due to illness in the control groups. The random effects meta-analysis for the adverse events is shown in Additional file 1: Figure S12.

### Explorative post-hoc subgroup analyses

Additional file 1: Figure S13, illustrates an explorative analysis of effect of interventions on PA sorted on type of control intervention and Additional file 1: Figure S14, illustrates an explorative analysis of effect of interventions on PA sorted on groupings of intervention types. However, none of the findings were significant. Additional file 1: Figure S15, illustrates an explorative analysis of effect of interventions on PA sorted on active control interventions versus non-active control interventions. The 11 study comparisons (1219 participants) with non-active control interventions had a significantly larger effect compared to the 10 study comparisons (1485 participants) with an active control intervention.

## Discussion

The objective of this systematic review was to investigate the effect of PAM-based interventions on older adults.

Our primary outcome of interest was PA and the main results include a moderate effect, equivalent to a larger increase on 1297 more steps per day in the intervention groups and the small to moderate effect on MVPA time equivalent to a larger increase on 8 more minutes per day in the intervention groups. As we were not able to explain the heterogeneity of the results with any of our sub- or sensitivity analyses, the effect of the interventions may be applicable to the broadly defined older adult population. However, further potential influences, such as medication and disease specific treatments need to be considered.

In terms of translating the effect on 1297 more steps per day, there is a lack of evidence on how much is clinically relevant change in general, for older adults. The WHO recommends that older adults are equally physically active as their younger counterparts but if co-morbidities limits their ability to be physically active, they should be as active as their conditions allow [43]. A systematic review suggests that the WHO-recommended 30 min of MVPA per day is equivalent to 7000 to 10,000 steps per day in older adults [43, 44]. According to Table 1, the median baseline daily step count in the studies was 5268 which makes the effect on 1297 steps equivalent to a 25% increase in daily number of steps. If the effect size is added to the median baseline daily step count, the average older adult will get close to 7000 steps per day. This highlights the clinical relevance of the results. Other more invasive exercise interventions may be more effective in increasing the amount of daily PA in older adults, but as PAM based interventions are not very invasive, they could be implemented in large scale projects as well.

This review provides evidence for the use of PAMs as an intervention to promote PA among older adults. Our finding of a moderate effect is in line with a former systematic review by Bravata et al. that estimated the effect size to be 2491 steps per day (95% CI: 1098 to 3885) in a population with a mean age on 49 years [8]. The population of interest in the Bravata et al. systematic review is more than 20 years younger than the median mean age in the included studies from this review [8]. As the level of physical activity decrease with age [2–5], a younger population is expected to be more active which may explain why the effect in steps per day is almost twice as large in the Bravata et al. systematic review [8]. However, as the effect sizes are not significantly different from each other, the above-mentioned explanation is only relevant if future systematic reviews find a significant effect modification from age, which we did not find in this review*.*

Even though we only included one study with results on sedentary time [13], this study was also included in a recent published systematic review from Qui et al. that reports PAM usage to be significantly associated with reduced sedentary time among adults [45].

Among older adults, level of PA is associated with, age, BMI and sex [46]. Contrary to this we were not able to explain the variance in the effect of the interventions through participant age, BMI or sex. However, this also means that we did not find any specific subgroup of older adults that may not benefit from using PAMs to enhance the level of physical activity.

The prevalence of frailty and chronic diseases are high in older adults [47, 48]. At first glance, our results could be limited to older adults with a higher function and a lower disease prevalence as the majority of the included studies included community dwelling older adults without specific diseases [11, 13–15, 24, 25, 32–35, 37, 38, 41, 42]. However, among these studies, several samples were inactive or did not meeting PA recommendations [11, 13, 32, 38]. One study was conducted in a post-acute care rehabilitation setting [14] and other studies included patients with hypertension [24], osteoarthritis [34], cancer [34], and other chronic diseases [35]. Four studies did not describe the disease characteristic of the participants [15, 24, 41, 42]. The broad range of participant characteristics across studies is a strength of this systematic review as it increases the generalizability of the findings to the general population.

None of the subgroup analysis showed any significant impact of risk of bias on the effect. We did however find an overestimation of the effect size on the PA caused by small study bias. Publication bias will normally overestimate the effect of the published interventions due to type 1 errors or selective outcome reporting [49]. In summary, we have chosen to downgrade the overall quality of the evidence due to publication bias.

We conducted three additional analyses to investigate the impact of intervention and control intervention content. PAM-based interventions had a significant greater effect in studies with non-active control interventions compared to studies with active control interventions. No other effects were significant. This analysis is recommended to obtain a meaningful estimate of the effect of the interventions and to avoid a confused picture of absolute intervention effects [50]. Using non-active controls will by nature give a larger effect size, as most interventions (also control interventions) will have some effect. Thus, future studies should use direct comparisons to investigate if PAMs can be an effective add-on *intervention, or if other types of behavior change strategies can effectively increase the effect from the PAMs.*

A Hawthorne-effect, meaning that the participants in control groups could be expected to increase their level of PA, simply due to participation in a PA study may be present in the included studies. This was also discussed in the systematic review published by Bravata et al. [8]. When comparing a PAM-based intervention where the participants receive feedback to a control group that are aware that they are being measured, the effect size might be slightly underestimated when compared to PAM-based walking programs with no control groups. This has been addressed systematically by Waters et al., who found a similar effect in both control and intervention groups in eight of 29 PA trials [51]. This is in line with our explorative results from Additional file 1: Figure S13 that illustrates a larger effect in studies that includes control groups who were asked to maintain usual PA and Additional file 1: Figure S15 that illustrates a larger effect size in studies that uses non-active control interventions. This may be explained by participants who volunteer for trials because they wish to increase their level of PA, participants being refractory or nonadherent after being allocated to a control group and several other factors which should be kept in mind when interpreting results from PA trials or reviews.

### Limitations

There were some deviations from the published study protocol [19]. Firstly, there were insufficient data to determine if participants met the study-specific recommendation for level of physical activity. We proposed to study this outcome in our protocol; however, none of the studies included in this review reported on this outcome. Secondly, we chose to pool the moderate and vigorous activity as most of the included studies did not distinguish between these intensity categories in their reporting.

We performed a wide and comprehensive literature search across several relevant databases, and used a pearl growing strategy where two reviewers independently located relevant references through journal sites and reference lists of included studies. Additionally, we obtained relevant references by using forward and backwards reference searches. Despite this wide and robust search strategy, it is possible that not all relevant studies were included in this systematic review.

In terms of translating the SMDs back to number of steps, MVPA time, meters walked in a 6MWT and BMI, the translation should only be read as a way of making our results easier to interpret and comes with limitations to generalizability. Firstly, we assume that the SMD can be used to extrapolate results, but some studies used different scales and outcome measures which might bring some problems. Secondly, the true SD of the population is impossible to estimate. However, when choosing the SDs from the largest study with the lowest risk of bias rating we have tried to be transparent and avoid bias in the selection. It should be noted that interpretation must happen with caution as it basically only represents the study from which the SD was chosen.

This systematic review is focused on older adults above the age of 65 years. As reported in Table 1, some of the studies will include results from participants younger than 65 which might bring some bias to our external validity. However, according to Additional file 1: Table S3, the association between study mean age and the effect size was clearly not significant for all outcomes and the study mean age explained almost no effect size heterogeneity. We hereby acknowledge the limitation that some included studies would have had younger participants, but we find no evidence for affecting the external validity to the population of interest.

### Body of evidence

We found that the quality of the body of evidence of PAM-based interventions was low to moderate. Our results were affected by unexplained heterogeneity, publication bias and imprecision. The pooled effects for time spent sedentary, physical capacity, BMI and self-reported HRQoL were not significant. Furthermore, the confidence interval for the effect size of the primary outcome, PA, suggests that the overall effect is small to moderate. However, a moderate quality of evidence was found on the risk of adverse events being the same in the intervention and the control groups. PAMs seem useful for public health interventions as it seems to be safe and effective to include them in PA programs for old adults. The grading of the body of evidence for each outcome is reported in the summary of findings table (Additional file 2). 

## Conclusion

### General interpretation of results

This review demonstrates low quality of evidence for a moderate effect on PA, equivalent to a larger increase at 1297 more steps per day, when comparing PAM-based interventions with control interventions in 21 studies. Furthermore, this review demonstrates moderate quality of evidence for a small to moderate effect on MVPA time equivalent to 8 more minutes per day. This review did not find an effect on physical capacity, BMI or HRQoL. Given the heterogeneity of the study samples, the results are likely to be applicable to a broad older population, but medication and disease specific treatments need to be considered.

### Implications for future practice and research

It seems safe and feasible to use PAMs in PA interventions in older adults. To avoid publication bias and unexplained heterogeneity, more randomized studies with high methodological quality and large sample sizes, are needed to determine possible participant characteristics associated with the adherence to and effect of the interventions. Furthermore, future studies should investigate if PAMs should be included as add-on interventions, or if other types of behavior change strategies should be applied to PAM-based interventions. The evolution of Internet of Things in medicine will emerge and have a great impact on how clinical decision making, preventive medicine and rehabilitation will take place in the future [52, 53]. To ensure that the costs and expenses are used correctly, it seems highly important to have ongoing reviewing of the literature and to include recent published RCTs in updated version of systematic reviews in this area of behavioral intervention research.

### Summary box (bullets)

- PAM-based interventions seem to be safe and effective in enhancing the level of PA in older adults.
- Low quality of evidence exists for PAM-based interventions having a moderate effect on PA, equivalent to 1297 more steps per day.
- Moderate quality of evidence exists for PAM-based interventions having a small to moderate effect on MVPA time equivalent to 8 more minutes per day.
- This review could not demonstrate an effect of PAM intervention on physical capacity, BMI or HRQoL.
- Future studies should not use non-active control interventions but instead compare PAM-based interventions with other active interventions or conduct add-on designs to investigate if the effect size of the PAM-intervention can be increased.

## Additional files

Additional file 1: **Figure S6.** Subgroup analysis on effect of the interventions on physical activity sorted on type of physical activity monitor, diagnoses, feedback frequency, risk of bias judgement and type of physical activity measure. Results are from random effects model using Hedges g. K: Number of studies; SMD: standardized mean difference; PAM: physical activity monitor; COPD: chronic obstructive pulmonary disease. For each analysis, the diamond represents the standardized mean difference of the pooled intervention effect with the horizontal line representing 95% confidence intervals. **Figure S7.** Subgroup analysis on effect of the interventions on moderate to vigorous physical activity, sorted on type of physical activity monitor, diagnoses, feedback frequency, and risk of bias judgement. Results are from random effects model using Hedges g. K: Number of studies; SMD: standardized mean difference; PAM: physical activity monitor; COPD: chronic obstructive pulmonary disease. For each analysis, the diamond represents the standardized mean difference of the pooled intervention effect with the horizontal line representing 95% confidence intervals. **Figure S8.** Subgroup analysis on effect of the interventions on physical capacity, sorted on type of physical activity monitor, diagnoses, feedback frequency, and risk of bias judgement. Results are from random effects model using Hedges g. K: Number of studies; SMD: standardized mean difference; PAM: physical activity monitor; COPD: chronic obstructive pulmonary disease. For each analysis, the diamond represents the standardized mean difference of the pooled intervention effect with the horizontal line representing 95% confidence intervals. **Figure S9.** Subgroup analysis on effect of the interventions on body mass index, sorted on type of physical activity monitor, diagnoses, feedback frequency, and risk of bias judgement. Results are from random effects model using Hedges g. K: Number of studies; SMD: standardized mean difference; PAM: physical activity monitor; COPD: chronic obstructive pulmonary disease. For each analysis, the diamond represents the standardized mean difference of the pooled intervention effect with the horizontal line representing 95% confidence intervals. **Figure S10.** Subgroup analysis on effect of the interventions on health-related qualify of life, sorted on type of physical activity monitor, diagnoses, feedback frequency, and risk of bias judgement. Results are from random effects model using Hedges g. K: Number of studies; SMD: standardized mean difference; PAM: physical activity monitor; COPD: chronic obstructive pulmonary disease; HRQoL: Health-related quality of life. For each analysis, the diamond represents the standardized mean difference of the pooled intervention effect with the horizontal line representing 95% confidence intervals. Positive values favor the intervention. **Figure S11.** Funnel plot with Eggers line illustrating risk of publication bias in the analysis of effect of the interventions on physical activity. SMD: standardized mean difference. **Figure S12.** Random effects meta-analysis on withdrawals due to illness and adverse events. For each study, the diamond represents the specific relative risk of withdrawing with the horizontal line representing 95% confidence intervals. Results are from random effects model with relative risks. RR: Relative risk. The large diamond represents the pooled relative risk. Values below one equals more events in the intervention groups. **Figure S13.** Explorative subgroup analyses of effect of interventions on physical activity sorted on control intervention. For each study, the diamond represents the standardized mean difference of the intervention effect with the horizontal line representing 95% confidence intervals. Results are from random effects model using standardized mean difference (SMD) adjusted to Hedges g. PA: physical activity. The large diamonds represent the pooled standardized mean difference between the intervention groups and the control groups. Positive values favor the intervention. **Figure S14.** Explorative subgroup analyses of effect of interventions on physical activity sorted on additional intervention content. Results are from random effects model using standardized mean difference (SMD) adjusted to Hedges g. For each study, the diamond represents the standardized mean difference of the intervention effect with the horizontal line representing 95% confidence intervals. The large diamonds represent the pooled standardized mean difference between the intervention groups and the control groups. Positive values favor the intervention. **Figure S15.** Figure S15. Explorative subgroup analyses of effect of interventions on physical activity sorted on active control intervention or non-active control intervention. Results are from random effects model using standardized mean difference (SMD) adjusted to Hedges g. For each study, the diamond represents the standardized mean difference of the intervention effect with the horizontal line representing 95% confidence intervals. The large diamonds represent the pooled standardized mean difference between the intervention groups and the control groups. **Table S1.** Characteristics of included studies. **Table S2.** Univariate meta-regressions between standardized mean differences from all outcomes and age, gender distribution, number of participants with walking aids, intervention length, baseline physical activity and body mass index. **Table S3.** Citations and reasons for exclusion from full text screening. (DOCX 4040 kb)

Additional file 2: Summary of findings table. (DOCX 17 kb)

## Abbreviations

6MWT: Six-minute walking test
95%CI: 95% confidence interval
BMI: Body mass index
HRQoL: Health-related quality of life
MVPA: Moderate to vigorous physical activity
PA: Physical Activity
PAMs: Physical activity monitors
RCT: Randomized controlled trial
SD: Standard deviation
SMD: Standard mean difference
