# Supplementary material for: Physical activity monitors to enhance amount of physical activity in older adults – a systematic review and meta-analysis
Source: Eur Rev Aging Phys Act. 2019 May 4;16:7. doi: 10.1186/s11556-019-0213-6 (PMC6500067; doi:10.1186/s11556-019-0213-6)
Supplement: Supplementary file 1 — Figure S6. Subgroup analysis on effect of the interventions on physical activity sorted on type of physical activity monitor, diagnoses, feedback frequency, risk of bias judgement and type of physical activity measure. Results are from random effects model using Hedges g. K: Number of studies; SMD: standardized mean difference; PAM: physical activity monitor; COPD: chronic obstructive pulmonary disease. For each analysis, the diamond represents the standardized mean difference of the pooled intervention effect with the horizontal line representing 95% confidence intervals. Figure S7. Subgroup analysis on effect of the interventions on moderate to vigorous physical activity, sorted on type of physical activity monitor, diagnoses, feedback frequency, and risk of bias judgement. Results are from random effects model using Hedges g. K: Number of studies; SMD: standardized mean difference; PAM: physical activity monitor; COPD: chronic obstructive pulmonary disease. For each analysis, the diamond represents the standardized mean difference of the pooled intervention effect with the horizontal line representing 95% confidence intervals. Figure S8. Subgroup analysis on effect of the interventions on physical capacity, sorted on type of physical activity monitor, diagnoses, feedback frequency, and risk of bias judgement. Results are from random effects model using Hedges g. K: Number of studies; SMD: standardized mean difference; PAM: physical activity monitor; COPD: chronic obstructive pulmonary disease. For each analysis, the diamond represents the standardized mean difference of the pooled intervention effect with the horizontal line representing 95% confidence intervals. Figure S9. Subgroup analysis on effect of the interventions on body mass index, sorted on type of physical activity monitor, diagnoses, feedback frequency, and risk of bias judgement. Results are from random effects model using Hedges g. K: Number of studies; SMD: standardized mean difference; PAM: physi [file 11556_2019_213_MOESM1_ESM.docx]

# Additional file 1

#
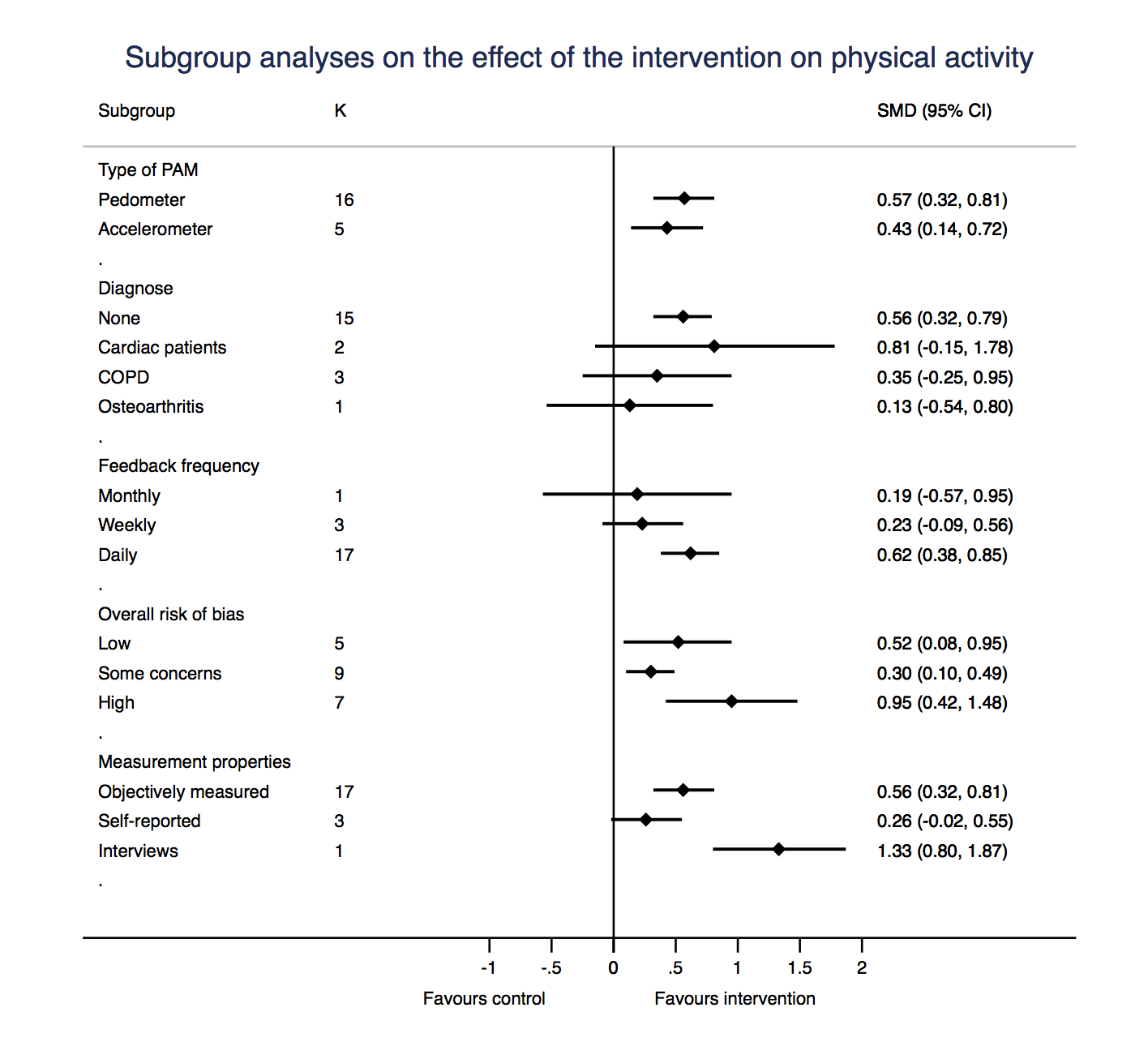


**Figure S6.** Subgroup analysis on effect of the interventions on physical activity sorted on type of physical activity monitor, diagnoses, feedback frequency, risk of bias judgement and type of physical activity measure. Results are from random effects model using Hedges g. K: Number of studies; SMD: standardized mean difference; PAM: physical activity monitor; COPD: chronic obstructive pulmonary disease. For each analysis, the diamond represents the standardized mean difference of the pooled intervention effect with the horizontal line representing 95% confidence intervals.


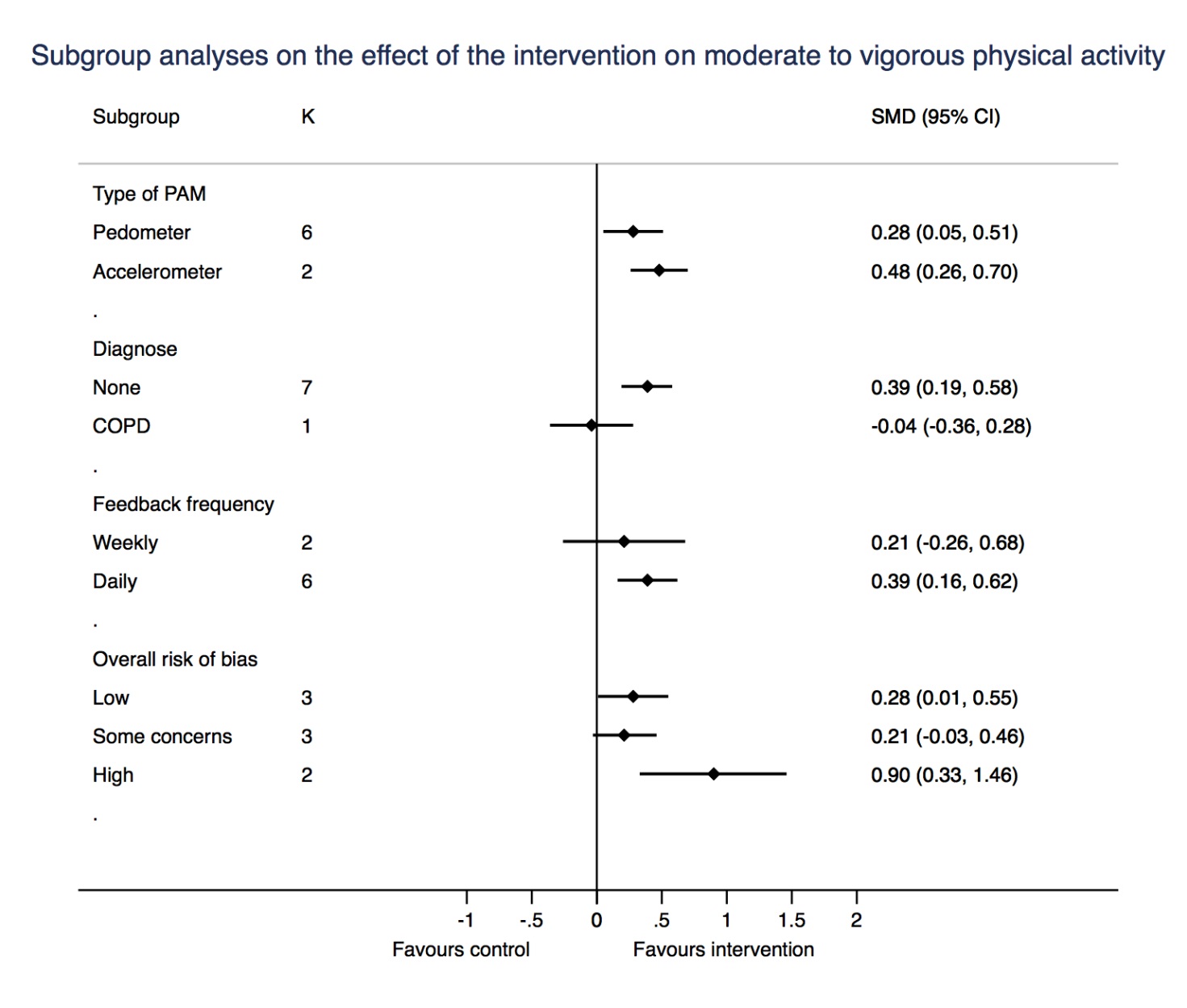


**Figure S7.** Subgroup analysis on effect of the interventions on moderate to vigorous physical activity, sorted on type of physical activity monitor, diagnoses, feedback frequency, and risk of bias judgement. Results are from random effects model using Hedges g. K: Number of studies; SMD: standardized mean difference; PAM: physical activity monitor; COPD: chronic obstructive pulmonary disease. For each analysis, the diamond represents the standardized mean difference of the pooled intervention effect with the horizontal line representing 95% confidence intervals.


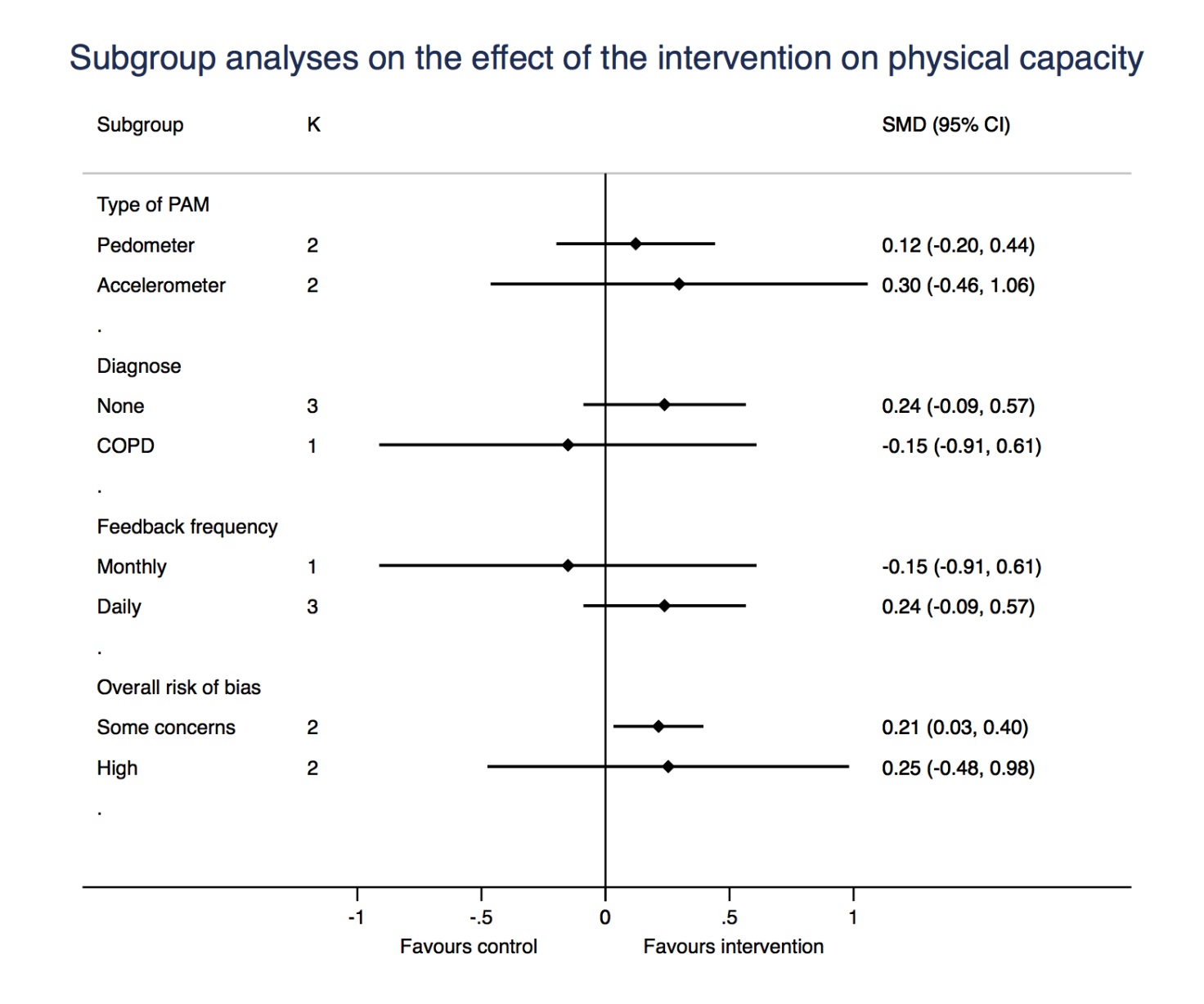


**Figure S8.** Subgroup analysis on effect of the interventions on physical capacity, sorted on type of physical activity monitor, diagnoses, feedback frequency, and risk of bias judgement. Results are from random effects model using Hedges g. K: Number of studies; SMD: standardized mean difference; PAM: physical activity monitor; COPD: chronic obstructive pulmonary disease. For each analysis, the diamond represents the standardized mean difference of the pooled intervention effect with the horizontal line representing 95% confidence intervals.


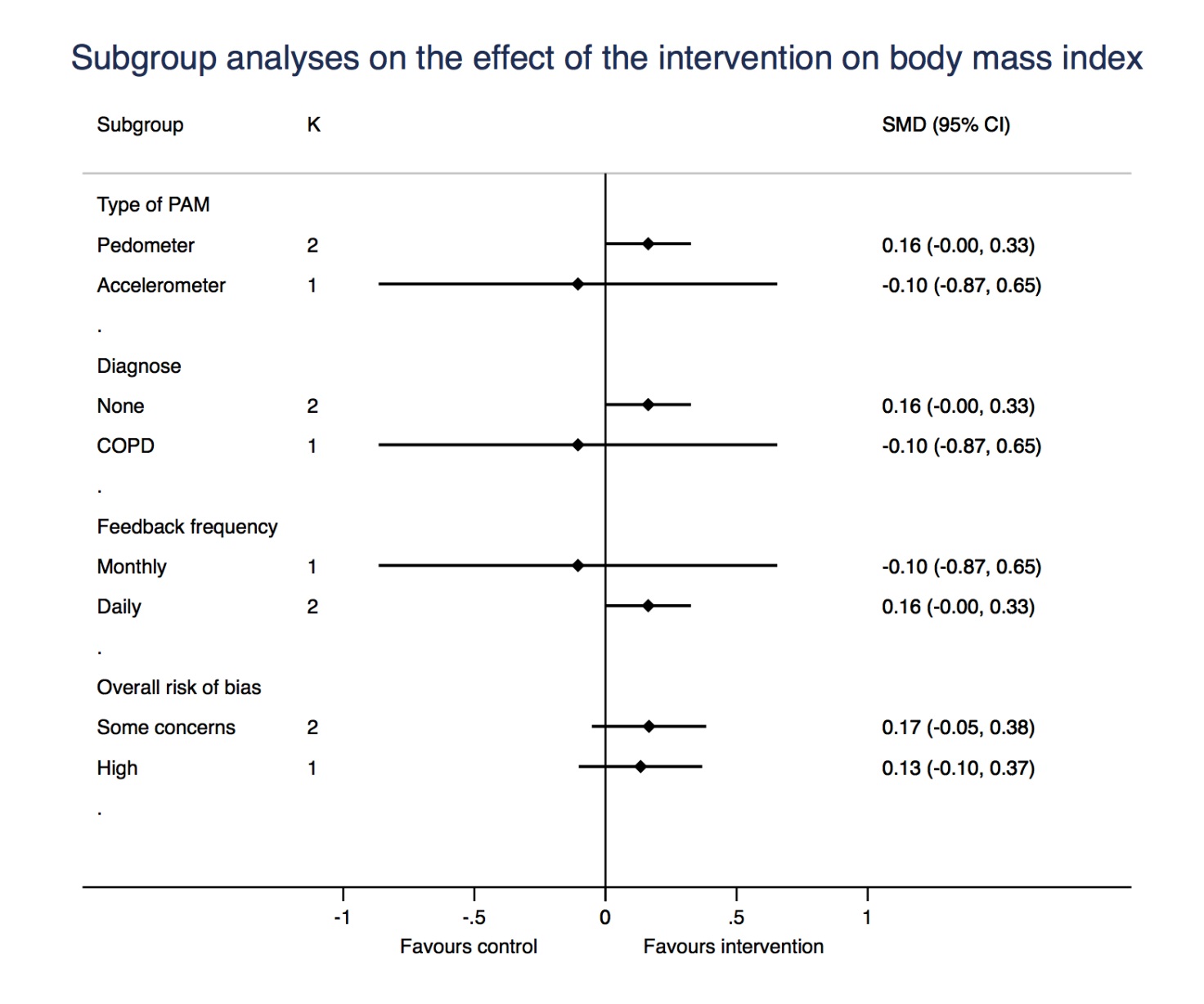


**Figure S9.** Subgroup analysis on effect of the interventions on body mass index, sorted on type of physical activity monitor, diagnoses, feedback frequency, and risk of bias judgement. Results are from random effects model using Hedges g. K: Number of studies; SMD: standardized mean difference; PAM: physical activity monitor; COPD: chronic obstructive pulmonary disease. For each analysis, the diamond represents the standardized mean difference of the pooled intervention effect with the horizontal line representing 95% confidence intervals.


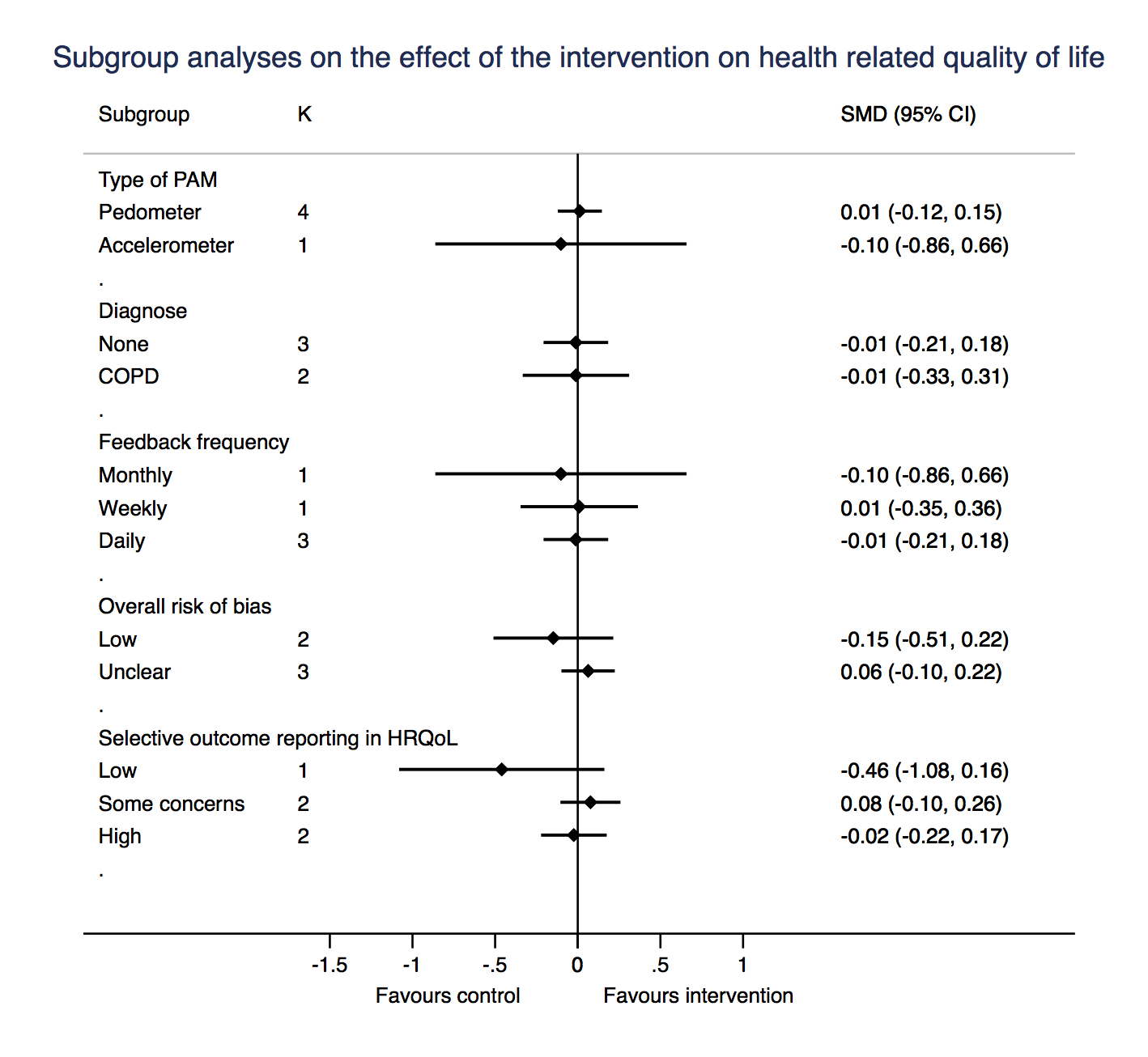


**Figure S10.** Subgroup analysis on effect of the interventions on health-related qualify of life, sorted on type of physical activity monitor, diagnoses, feedback frequency, and risk of bias judgement. Results are from random effects model using Hedges g. K: Number of studies; SMD: standardized mean difference; PAM: physical activity monitor; COPD: chronic obstructive pulmonary disease; HRQoL: Health-related quality of life. For each analysis, the diamond represents the standardized mean difference of the pooled intervention effect with the horizontal line representing 95% confidence intervals. Positive values favor the intervention.


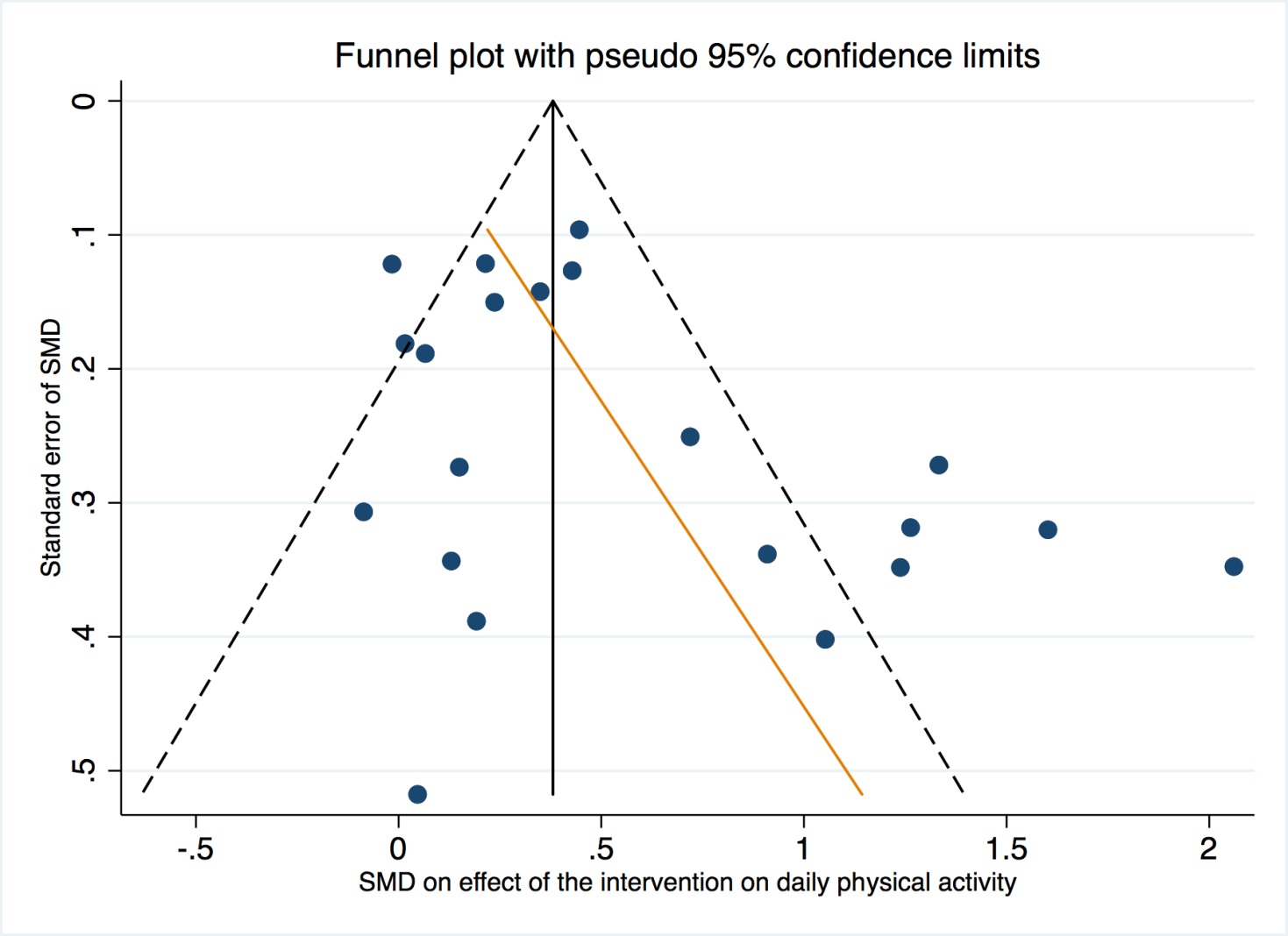


**Figure S11.** Funnel plot with Eggers line illustrating risk of publication bias in the analysis of effect of the interventions on physical activity. SMD: standardized mean difference.


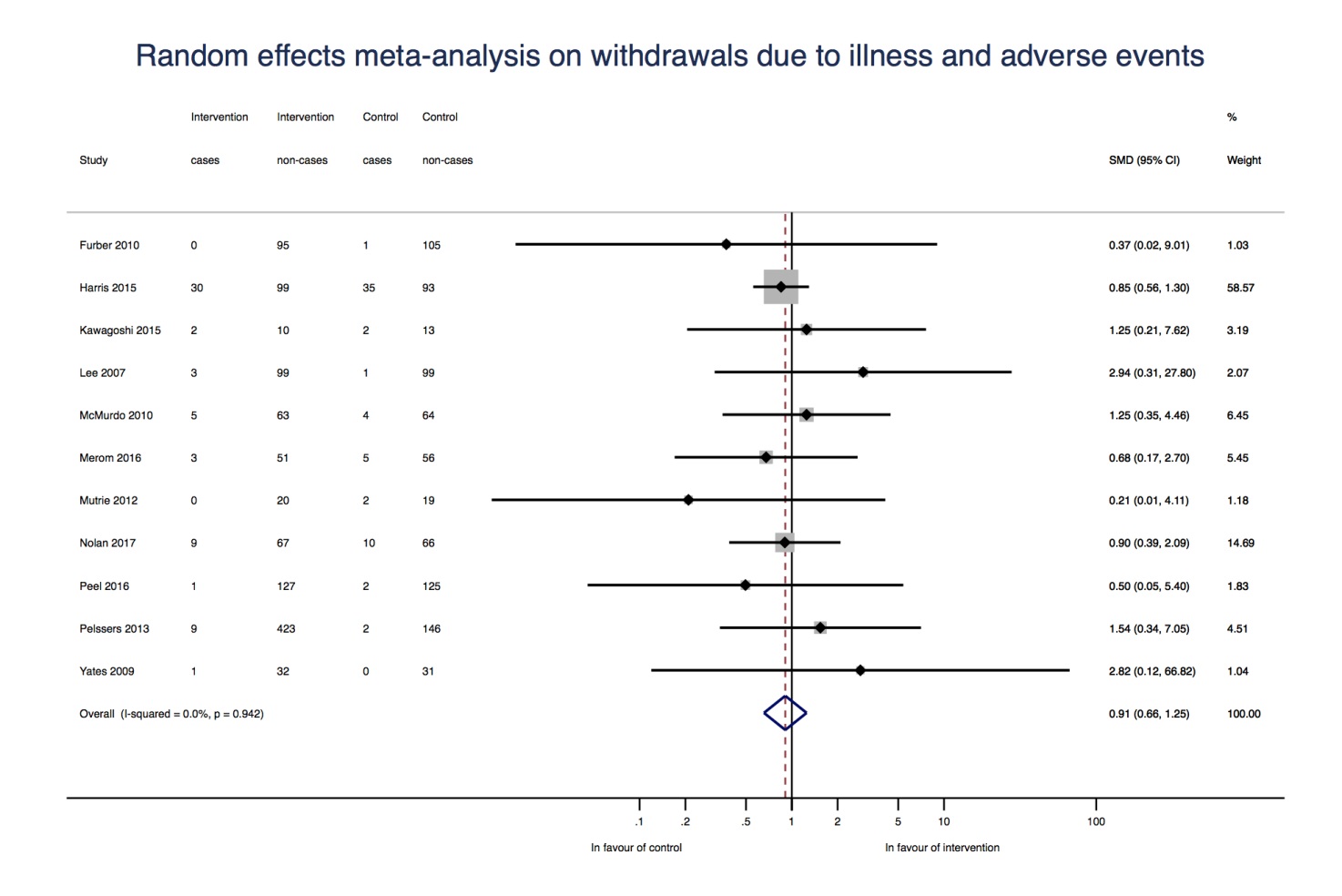


**Figure S12.** Random effects meta-analysis on withdrawals due to illness and adverse events. For each study, the diamond represents the specific relative risk of withdrawing with the horizontal line representing 95% confidence intervals. Results are from random effects model with relative risks. RR: Relative risk. The large diamond represents the pooled relative risk. Values below one equals more events in the intervention groups.


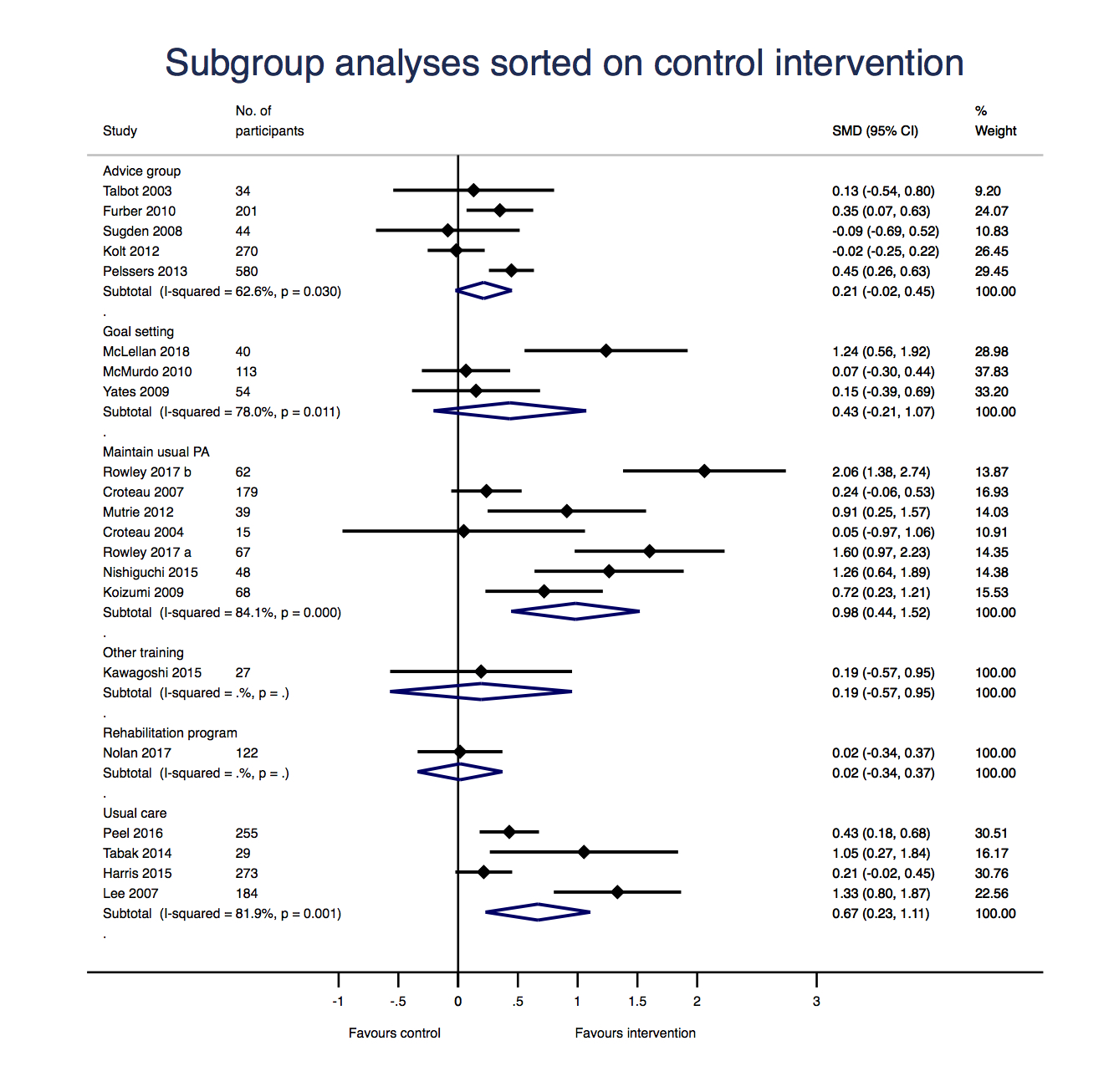
**Figure S13.** Explorative subgroup analyses of effect of interventions on physical activity sorted on control intervention. For each study, the diamond represents the standardized mean difference of the intervention effect with the horizontal line representing 95% confidence intervals. Results are from random effects model using standardized mean difference (SMD) adjusted to Hedges g. PA: physical activity. The large diamonds represent the pooled standardized mean difference between the intervention groups and the control groups. Positive values favor the intervention.


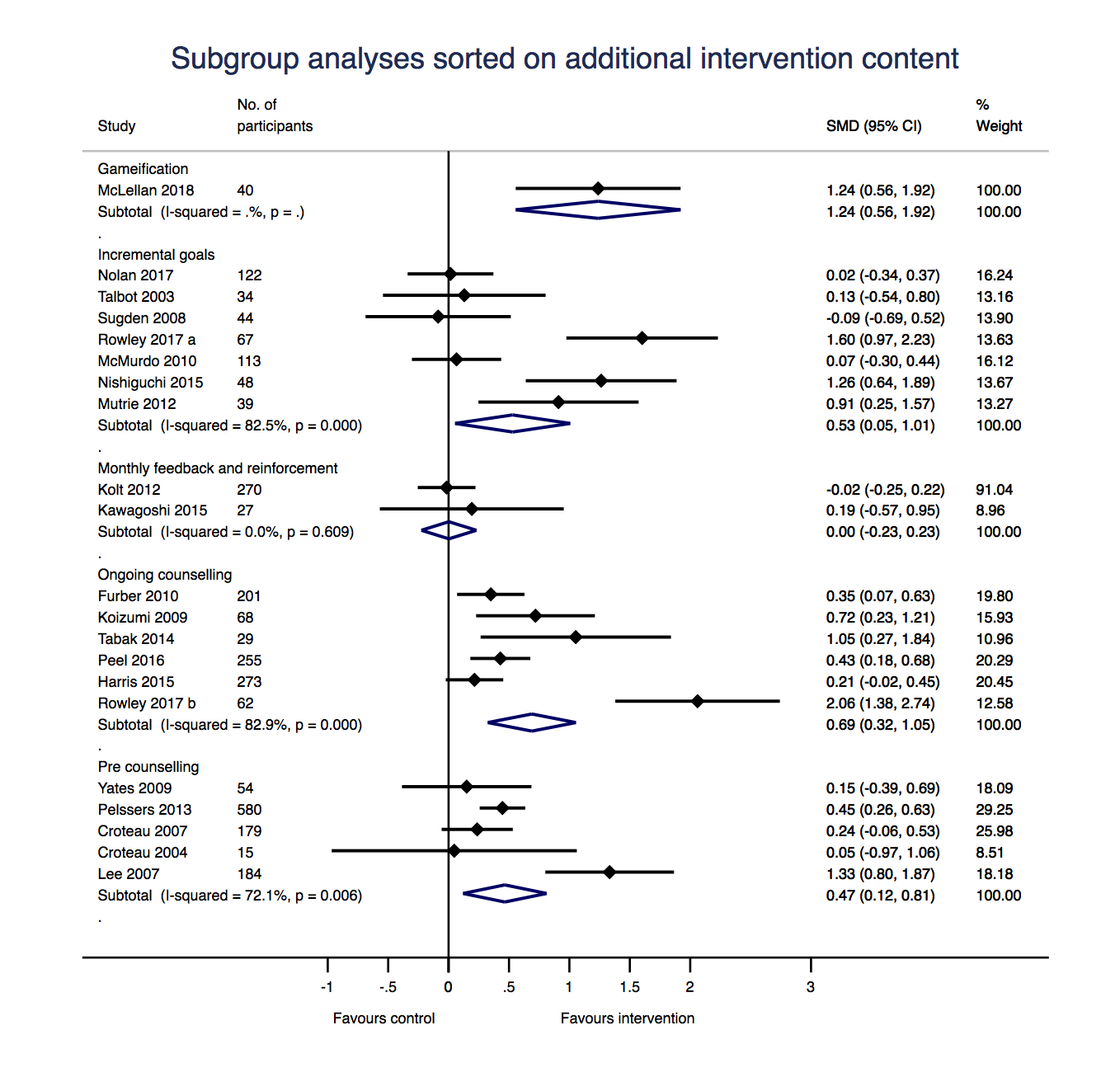


**Figure S14.** Explorative subgroup analyses of effect of interventions on physical activity sorted on additional intervention content. Results are from random effects model using standardized mean difference (SMD) adjusted to Hedges g. For each study, the diamond represents the standardized mean difference of the intervention effect with the horizontal line representing 95% confidence intervals. The large diamonds represent the pooled standardized mean difference between the intervention groups and the control groups. Positive values favor the intervention.


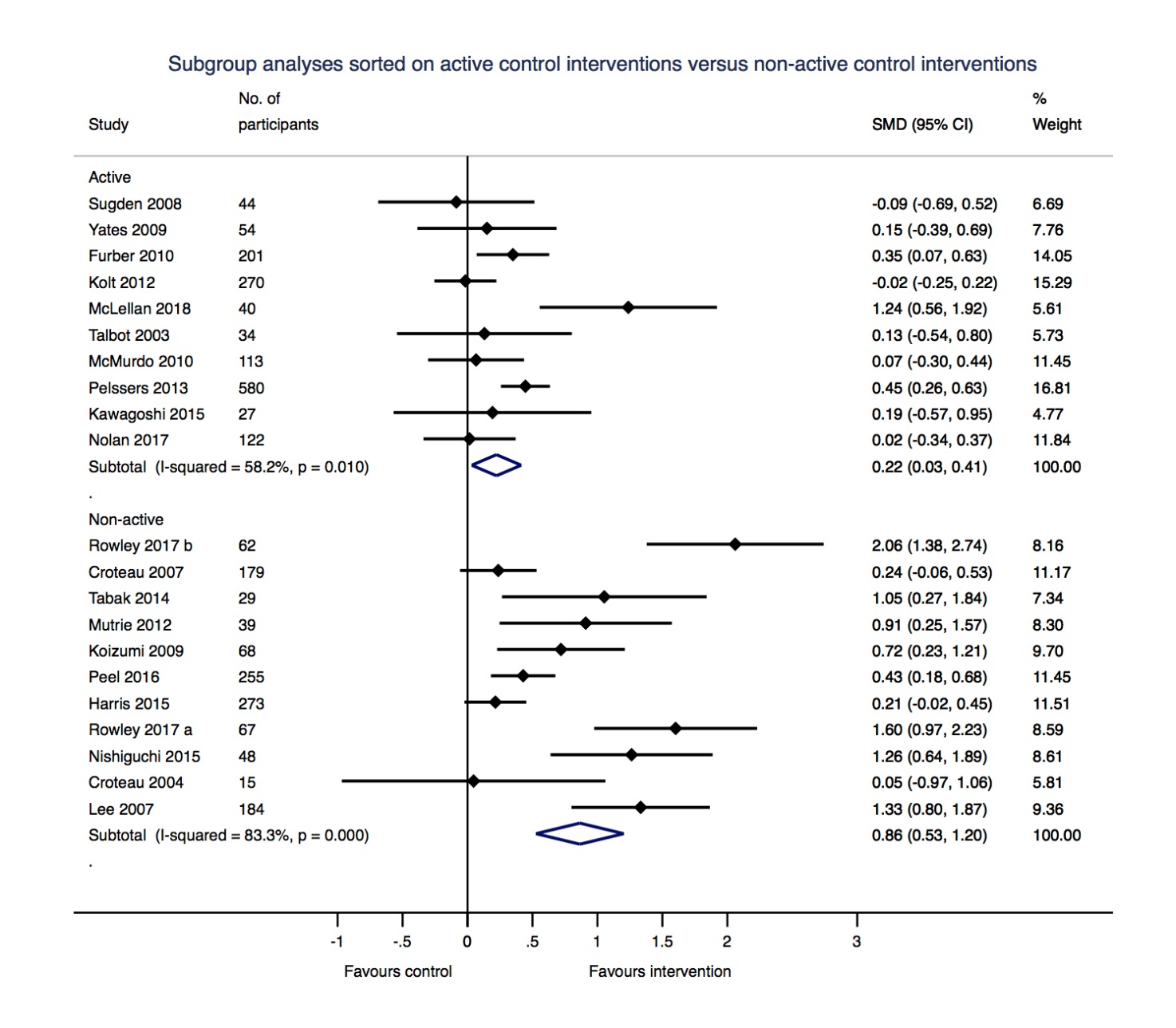


**Figure S15.** Explorative subgroup analyses of effect of interventions on physical activity sorted on active control intervention or non-active control intervention. Results are from random effects model using standardized mean difference (SMD) adjusted to Hedges g. For each study, the diamond represents the standardized mean difference of the intervention effect with the horizontal line representing 95% confidence intervals. The large diamonds represent the pooled standardized mean difference between the intervention groups and the control groups.

**Table S1.** Characteristics of included studies

***Croteau 2004***

| **Methods** | **Study design:** Randomized controlled trial  **Study grouping:** Parallel group  **Number of arms:** 2  **Informed consent and ethical approval:** Before participating in the study, all participants read and signed an informed consent form approved by the University of Southern Maine’s Institutional Review Board. |
| --- | --- |
| **Participants** | **Baseline Characteristics**  Intervention   - *Age*: 78 (7.57) - *Gender*: 14% male - *Osteoarthritis*: 100% - *Hypertension*: 85.7% - *Coronary Artery Disease*: 28.6% - *Cancer*: 42.9% - *Assistive devices*: -   Control   - *Age*: 83 (6.71) - *Gender*: 0% male - *Osteoarthritis*: Control 87.5% - *Hypertension*: 75% - *Coronary Artery Disease*: 50% - *Cancer*: 37.5%   Overall   - *Age*: IV 78 (7.57) / Control 83 (6.71) - *Gender*: IV Overall 7% male / 14% male/Control 0% male - *Osteoarthritis*: IV 100%/Control 87.5% - *Hypertension*: IV 85.7% /Control 75% - *Coronary artery disease*: IV 28.6%/Control 50% - *Cancer*: IV 42.9% / Control 37.5% - *Assistive devices*: "significantly more in IV group compared to control group"   **Included criteria:** The selection criteria for admission into the study were:(1) 68 years of age, (2) 23 on the Mini-Mental State Examination, (3)10 on the Performance Battery Test, (4) ambulates independently (assistive-devices permitted), and (5) not currently enrolled in a structured exercise program.  **Excluded criteria:** -  **Pretreatment:** -  **Total number of participants:** 15  **possible diagnostic criteria:** -  **country:** USA |
| **Interventions** | **Intervention Characteristics**  **Name of intervention:** Life Steps program  **Duration:** 4 weeks  **Short description:** *Individually tailored intervention utilizing behavioral components of Social Cognitive Theory. The intervention consisted of a counseling session followed by 4 weeks of pedometer usage and weekly follow-up.*  **Placement of Physical Activity Monitor:** Hip  **Type of Physical Activity Monitor:** Pedometer  **Feedback frequency:** Weekly  **Control Intervention:** Maintain usual physical activity |
| **Outcomes** | *Average daily steps*   - **Outcome type**: Continuous Outcome - **Reporting**: Fully reported - **Unit of measure**: Steps per day - **Direction**: Higher is better - **Data value**: Change from baseline |
| **Identification** | **Sponsorship source:** Research was supported by grants from University of Southern Maine College of Nursing and Health Professions, Payson Funds.  **Country:** USA, Maine  **Setting:** The participants of this pilot study consisted of 15 older adults (68 years of age) who live in a community-based, subsidized, assisted-living facility.  **Comments:** None  **Authors name:** Karen A. Croteau, EdD, Assistant Professor of Sports Medicine  **Institution:** Department, College of Nursing and Health Professions, University of Southern Maine  **Email:** kcroteau@usm.maine.edu  **Address:** 219 Costello Complex, Gorham, ME 04038 (E-mail: kcroteau@usm.maine.edu).  **Aim:** Given the need for improving mobility levels of older adults and the recommended use of pedometers in physical activity interventions, the primary purpose of this study was to examine the influence of a pedometer-based intervention on physical activity levels, mobility-related self-efficacy and functional mobility of older adults residing in an assisted-living facility. Given the gap in literature related to physical activity interventions for older adults residing in assisted living facilities, the researchers felt this was an important population to study. Additionally, the group of researchers comprised an interdisciplinary team consisting of two Certified Therapeutic Recreation Specialists™, a Registered Nurse and a Health Fitness Instructor  **Key conclusion:** There were no significant differences between the groups. |
| **Notes** | Paper: <http://www.tandfonline.com/doi/abs/10.1300/J016v28n02_02> |

Risk of bias table

| **Bias** | **Authors' judgement** | **Support for judgement** |
| --- | --- | --- |
| Bias arising from the randomization process | Unclear risk | Question 1.1 Yes, the allocation sequence was random (flip of a coin)  Question 1.2 No information about concealment of allocation sequence  Question 1.3 There were no baseline imbalances to suggest a problem with the randomization. |
| Bias due to deviations from intended interventions | Unclear risk | Question 2.1 Participants were probably aware of intervention  Question 2.2 Personnel were probably aware of intervention  Question 2.3 Probably no deviations from intended interventions  Question 2.4 Deviations did probably not affect outcome  Question 2.5 No information  Question 2.6 No information |
| Bias due to missing outcome data | Low risk | Question 3.1 Outcome data were probably available for all participants. |
| Bias in measurement of the outcome | Low risk | Question 4.1 Outcome assessors were probably aware of intervention  Question 4.2 The assessment of the outcome were not likely to be affected by knowledge on the intervention. |
| Bias in selection of the reported result All outcomes | Unclear risk | Question 5.1 and 5.1 No information |
| Overall bias All outcomes | Unclear risk |  |

***Croteau 2007***

| **Methods** | **Study design:** Randomized controlled trial  **Study grouping:** Crossover  **Aim:** This study aimed to investigate the effect of a 12-week pedometer-based intervention on the daily step counts of community-re-siding older adults and to determine its effectiveness at 12-weeks postintervention.  **Number of groups:** 2  **Ethical approval and informed consent:** Before the study, all participants read and signed an informed consent form approved by the primary researcher’s institutional review board. |
| --- | --- |
| **Participants** | **Baseline Characteristics**  Intervention   - *Age*: 74.4 years, SD (9.1)   Control   - *Age*: 71.2 years, SD (8.2)   Overall   - *Age*: 72.9 years, SD (8.8) - *Sex*: 22% male - *Country*: USA - *Co-morbidities*: Yes   **Included criteria:** Participants were selected if they met the following criteria: (a) > 55 years of age, (b) able to ambulate independently, (c) able to walk at a velocity and/or with appropriate gait patterns necessary to permit adequate pedometer readings, (d) wear clothing that permits appropriate pedometer placement, and (e) have not had a physician indicate that increased physical activity would be harmful.  **Excluded criteria:** NA  **Pretreatment:** NA  **Total number of participants:** 147 (115 women, 32 men)  **Possible diagnostic criteria:** NA  **Co-morbidities:** Hypertension (48.3%), osteo-arthritis (36.7%), current/previous cancer (17.7%), osteoporosis (12.9%), lipid disorders (12.9%), depression (10.2%), coronary artery disease (8.8%), and Type II diabetes (8.8%). |
| **Interventions** | **Intervention Characteristics**  **Name of intervention: -**  **Duration:** 12 Weeks  **Short description:** *On completion of pre-test data collection, intervention group participants met individually with a facilitator to set daily step goals, select strategies for increasing daily step counts, review pedometer usage, and discuss procedures for keeping a step calendar. From a list of sample strategies (such as parking further away in a parking lot, walking while waiting for an appointment, walking with a friend, taking your dog for an extra walk, etc.) and through brainstorming, participants selected strategies they wanted to use to increase their daily physical activity.*  **Placement of Physical Activity Monitor:** Hip  **Type of Physical Activity Monitor:** Pedometer  **Feedback frequency:** Daily  **Control Intervention:** Maintain usual physical activity |
| **Outcomes** | *Daily step scores among the groups*   - **Outcome type**: Continuous Outcome - **Reporting**: Fully reported - **Unit of measure**: Steps per day - **Direction**: Higher is better - **Data value**: Endpoint - **Notes**: Baseline |
| **Identification** | **Sponsorship source:** This study was supported by a research grant from the College of Nursing and Health Professions at the University of Southern Maine. The authors wish to thank Linda Buettner, Suzanne Fitzsimmons, and Jeanne Csuy from Florida Gulf Coast University, Andy McMahon, Kristine Sterling, and Sara Goodine from the University of Southern Maine, and the staff at the Partnership for Healthy Aging and the Learning Resource Center of Maine Medical Center, for their contributions to this study. Please address all correspondence concerning this article to Karen A. Croteau, 219 Costello Complex, University of Southern Maine, Gorham, ME 04038.  **Country:** USA, Maine  **Setting:** Participants were recruited from various health, educational, and social programs offered in the Portland, ME, and Port Charlotte, FL, areas, as both regions have high concentrations of older adults  **Comments:** None  **Authors name:** Karen A. Croteau, Nancy E. Richeson, Bonnie C. Farmer, and David B. Jones  **Institution:** Karen A. Croteau is with the Department of Exercise, Health, and Sport Sciences at the University of Southern Maine.  **Email:** kcroteau@usm.maine.edu  **Address:** Karen A. Croteau, 219 Costello Complex, University of Southern Maine, Gorham, ME 04038  **Key conclusions:** The pedometer-based intervention was effective in increasing participants' daily step counts. |
| **Notes** | Paper:  <http://www.tandfonline.com/doi/abs/10.1080/02701367.2007.10599439>  Paper with results:  <https://www.ncbi.nlm.nih.gov/pmc/articles/PMC3501246/>  Study design paper:  <https://www.ncbi.nlm.nih.gov/pmc/articles/PMC3050749/>  Head author has been contacted to get full data (without exclusion of the outliers).  Access to end-point data has been obtained. |

Risk of bias table

| **Bias** | **Authors' judgement** | **Support for judgement** |
| --- | --- | --- |
| Bias arising from the randomization process | Unclear risk | Question 1.1 No information about randomization  Question 1.2 No information about concealment of allocation sequence  Question 1.3 There were no baseline imbalances to suggest a problem with the randomization. |
| Bias due to deviations from intended interventions | Unclear risk | Question 2.1 Participants were probably aware of intervention  Question 2.2 Personnel were probably aware of intervention  Question 2.3 Probably no deviations from intended interventions  Question 2.4 Deviations did probably not affect outcome  Question 2.5 No information  Question 2.6 No information |
| Bias due to missing outcome data | High risk | More than 20% dropout and no available data on those. |
| Bias in measurement of the outcome | Low risk | Question 4.1 Outcome assessors were probably aware of the intervention received by the study participants.  Question 4.2 The assessment were probably not likely to be influenced by the knowledge. |
| Bias in selection of the reported result All outcomes | Unclear risk | No information about 5.1 and 5.2 |
| Overall bias All outcomes | High risk |  |

***Furber 2010***

| **Methods** | **Study design:** Randomized controlled trial  **Study grouping:** Parallel group  **Number of groups:** 2  **Informed consent obtained and ethical approval.:** When a patient had verbally agreed to enroll into the study they were sent additional information and an informed consent form to sign and return to the researcher.... The study was approved by the Human Research Ethics Committees of the University of Wollongong/Illawarra Area Health Service and the University of New South Wales. |
| --- | --- |
| **Participants** | **Baseline Characteristics**  Intervention   - *Age*: 66.7 (10.6) - *Gender*: 71.2% male - *Body mass index*: 27.3 (5.2) - *Married*: 74.0% - *Education (Secondary level or less)*: 67.3% - *Education (Tertiary level)*: 32.7% - *Active above 150 min/week*: 57.7% - *Born in Australia*: 79.8% - *Coronary artery bypass graft surgery*: 14.4% - *Percutaneous coronary intervention*: 22.1% - *Myocardial infarction*: 28.9% - *Acute coronary syndrome*: 19.2% - *Other co-morbidity*: 15.4%   Control   - *Age*: 65.4 (11.5) - *Gender*: 68.5% male - *Body mass index*: 27.8 (4.8) - *Married*: 73.9% - *Education (Secondary level or less)*: 63.1% - *Education (Tertiary level)*: 36.9% - *Active above 150 min/week*: 55.0% - *Born in Australia*: 68.5% - *Coronary artery bypass graft surgery*: 18.9% - *Percutaneous coronary intervention*: 25.2% - *Myocardial infarction*: 18.9% - *Acute coronary syndrome*: 19.8% - *Other co-morbidity*: 17.2%   **Included criteria:** Cardiac patients who were referred to an outpatient CRP in the Illawarra or Shoalhaven area of New South Wales, Australia, over a 10-month period during 2005–2006, were identified from a centralized referral database maintained by the area health service. Patients were eligible for this study if they had not attended an outpatient CRP, spoke English and had no co-morbidities affecting physical activity participation; they were invited to participate 2 months after discharge from hospital.  **Excluded criteria:** -  **Pretreatment:** - |
| **Interventions** | **Intervention Characteristics**  **Name of intervention: -**  **Duration:** Six months  **Short description:** *The intervention was based on social cognitive theory. The intervention strategies included a focus on increasing self-efficacy, increasing beliefs about the positive health consequences of taking action (outcome expectancies) and establishing physical activity goals. The intervention comprised a pedometer, a step calendar for self-monitoring, and telephone support which included goal setting and behavioral reinforcement.*  **Placement of Physical Activity Monitor:** Hip  **Type of Physical Activity Monitor:** Pedometer  **Feedback frequency:** Daily  **Control Intervention:** Advice group |
| **Outcomes** | *Self-reported walking mean minutes (SD)*   - **Outcome type**: Continuous Outcome - **Reporting**: Fully reported - **Unit of measure**: Minutes - **Direction**: Higher is better - **Data value**: Change from baseline   *Adverse events*   - **Outcome type**: Adverse Event - **Reporting**: Fully reported - **Direction**: Lower is better - **Data value**: Endpoint |
| **Identification** | **Sponsorship source:** Funding: NSW Health Promotion Research Demonstration Grant Scheme.  **Country:** Australia  **Setting:** Cardiac patients who were referred to an outpatient CRP in the Illawarra or Shoalhaven area of New South Wales, Australia,  **Comments:** -  **Authors name:** Susan Furber  **Institution:** South Eastern Sydney and Illawarra Area Health Service, NSW, Australia b University of New South Wales, Australia  **Email:** susan.furber@sesiahs.health.nsw.gov.au (S. Furber).  **Address:** South Eastern Sydney and Illawarra Area Health Service, Locked Bag 9, Wollongong, NSW 2500, Australia  **Aim:** The aim of this study was to determine the effectiveness of an individualized pedometer-based telephone intervention on physical activity levels of cardiac patients who were referred to, but could not, or chose not to attend, a CRP.  **Key conclusion:** The findings that the pedometer-based telephone intervention was successful in increasing physical activity levels in cardiac patients who did not attend a CRP could result in major health benefits for this group of people. |
| **Notes** | Paper:  <https://ac.els-cdn.com/S0738399109005898/1-s2.0-S0738399109005898-main.pdf?_tid=dc92c61e-efaa-11e7-8c8a-00000aab0f6b&acdnat=1514890458_f14ff7866e510e10ac4264ddcd6ab4bb>  Register/protocol: NA |

Risk of bias table

| **Bias** | **Authors' judgement** | **Support for judgement** |
| --- | --- | --- |
| Bias arising from the randomization process | Low risk | Question 1.1 Yes, the allocation sequence was random  Question 1.2 Yes, the allocation sequence was concealed  Question 1.3 There were no baseline imbalances to suggest a problem with the randomization. |
| Bias due to deviations from intended interventions | Unclear risk | Question 2.1 Participants were probably aware of intervention  Question 2.2 Personnel were probably aware of intervention  Question 2.3 No information  Question 2.4 Deviations did probably not affect outcome  Question 2.5 No information  Question 2.6 No information |
| Bias due to missing outcome data | High risk | Question 3.1 Outcome data were not available for all participants  Question 3.3 No evidence that the results are robust to the presence of missing outcome data (no ITT)  The only analysis used was complete case and thus, bias towards an overestimation of the effect exists. |
| Bias in measurement of the outcome | Low risk | Question 4.1 Outcome assessors were probably aware of the intervention received by the study participants.  Question 4.2 The assessment were probably not likely to be influenced by the knowledge. |
| Bias in selection of the reported result All outcomes | Unclear risk | Judgement Comment: No protocol/register has been located |
| Overall bias All outcomes | Unclear risk |  |

***Harris 2015***

| **Methods** | **Study design:** Randomized controlled trial  **Study grouping:** Parallel group  **Number of groups:** 2  **Informed consent obtained and ethical approval:** Interested patients attended a baseline assessment at the practice, where individual informed written consent was obtained. The trial was approved by Oxfordshire Research Ethics Committee C, UK (11/H0606/2). |
| --- | --- |
| **Participants** | **Baseline Characteristics**  Intervention   - *Age*: 67.5 (4.2) - *Gender (male)*: 47% - *Married*: 80% - *No chronic diseases*: 35% - *1-2 Chronic diseases*: 57% - *Above 3 chronic diseases*: 8% - *Body mass index above 25*: 62% - *Body mass index*: 27.1 (4.5)   Control   - *Age*: 65.9 (4.3) - *Gender (male)*: 46% - *Married*: 82% - *No chronic diseases*: 26% - *1-2 Chronic diseases*: 63% - *Above 3 chronic diseases*: 11% - *Body mass index above 25*: 72% - *Body mass index*: 27.6 (4.1)   Overall   - *Age*: 66.7 (4.3) - *Gender (male)*: 46% - *Married*: 80.5% - *No chronic diseases*: 31% - *1-2 Chronic diseases*: 60% - *Above 3 chronic diseases*: 10% - *Body mass index above 25*: 67% - *Body mass index*: 27.4 (4.3)   **Included criteria:** Eligible participants were patients aged 60–74 years registered at three general practices in Oxfordshire and Berkshire, UK, who could walk outside and had no contra-indications to increasing PA.  **Excluded criteria:** Patients were excluded if they were in a residential or nursing home or were identified as having a medical or psychiatric condition unsuitable for the intervention either through Read code from their electronic primary care records or by their general practitioner (family practice doctor) prior to invitation mail-out (S1 Text) [13].  **Pretreatment:** -  **Total number of participants:** 298 |
| **Interventions** | **Intervention Characteristics**  **Name of intervention:** PACE  **Duration:** One year  **Short description:** *The key components were as follows: (i) pedometers (SW-200, Yama Digi-Walker) given to participants by nurses at their first visit to record their step-counts in the PA diary; (ii) accelerometers (GT3X+, Actigraph) that participants were asked to wear before the second, third, and fourth nurse visits and from which nurses downloaded data during the consultation to show participants the time they were spending in different PA intensities;(iii) practice nurse consultations based on key BCTs including goal-setting, self-monitoring, building self-efficacy and social support, overcoming barriers, preventing relapses, and building lasting habits; (iv) PACE-Lift patient handbook, which supported BCTs, given to patients to keep at their first appointment and used during consultations; (v) individual walking/PA plan agreed upon during consultations between nurses and participants to encourage adding in both steps and time spent walking in moderate intensity PA, in bouts of at least 10 minutes, teach individual’s baseline; (vi) PA diary to record PA and step-counts, used with the monitors to set goals, monitor progress, and aid feedback, by relating specific diary activities to accelerometer recorded PA intensities.*  **Placement of Physical Activity Monitor:** Hip  **Type of Physical Activity Monitor:**  **Feedback frequency:** Daily  **Control Intervention:** Usual care |
| **Outcomes** | *Daily step count*   - **Outcome type**: Continuous Outcome - **Reporting**: Fully reported - **Unit of measure**: Steps per day - **Direction**: Higher is better - **Data value**: Change from baseline   *MVPA: Total weekly minutes*   - **Outcome type**: Continuous Outcome - **Unit of measure**: Minutes - **Direction**: Higher is better - **Data value**: Change from baseline   *Change in body mass index*   - **Outcome type**: Continuous Outcome - **Reporting**: Fully reported - **Unit of measure**: kg/m2 - **Direction**: Higher is better - **Data value**: Change from baseline   *Adverse events*   - **Outcome type**: Adverse Event - **Reporting**: Fully reported - **Direction**: Lower is better - **Data value**: Endpoint |
| **Identification** | **Sponsorship source:** Funding: This paper presents independent research funded by the National Institute of Health Research(NIHR) under its Research for patient benefit program (Grant reference number PB-PG-0909-20055). Authors who received funding were: TH DGCCRV SMK AW SI UE PHW CB. The funding body had no role in study design, data collection and analysis, decision to publish or preparation of the manuscript.  **Country:** United Kingdom  **Setting:** General practices in Oxfordshire and Berkshire, UK  **Comments:** -  **Authors name:** Tess Harris  **Institution:** Population Health Research Institute, St George’s University of London, London, United Kingdom  **Email:** tharris@sgul.ac.uk  **Address:** -  **Aim:** We therefore conducted a randomized trial to determine whether an intervention based on pedometer and accelerometer feedback combined with practice nurse PA consultations increases PA levels in 60–75-year-olds over 3 months and whether any change is maintained at12 months. Secondary aims were to assess whether effects were modified by age, gender, body mass index (BMI), disability, exercise self-efficacy or taking part as a couple, and to estimate effects on patient reported outcomes and anthropometric measures. The trial was designed to be population-based and to measure objectively assessed PA, including MVPA (providing a direct link to PA guidelines). Qualitative studies were undertaken with intervention participants and practice nurses to assess the intervention’s acceptability and the barriers and facilitators to increasing PA.  **Key conclusion:** The PACE-Lift trial increased both step-counts and objectively measured MVPA in 10-minute bouts in 60–75-year-olds at 3 and 12 months, with no effect on adverse events. |
| **Notes** | Paper:  <http://journals.plos.org/plosmedicine/article?id=10.1371/journal.pmed.1001783>  Protocol: <https://bmcpublichealth.biomedcentral.com/articles/10.1186/1471-2458-13-5>  Tess Harris has been contacted to obtain mean/SD data from table 2. Mean age (SD) and mean BMI (SD) was obtained from the head author and the study-statistician). |

Risk of bias table

| **Bias** | **Authors' judgement** | **Support for judgement** |
| --- | --- | --- |
| Bias arising from the randomization process | Unclear risk | Judgement Comment: The first selection of households lacks information. |
| Bias due to deviations from intended interventions | Unclear risk | Control group were aware of them being in the “control group” |
| Bias due to missing outcome data | Low risk | Question 3.1 Outcome data were probably available for all participants. |
| Bias in measurement of the outcome | Low risk | Question 4.1 Outcome assessors were probably aware of the intervention received by the study participants.  Question 4.2 The assessment were probably not likely to be influenced by the knowledge. |
| Bias in selection of the reported result All outcomes | Low risk | Question 5.1. Probably not  Question 5.2 Probably not |
| Overall bias All outcomes | Unclear risk | Judgement Comment: There are a lack of outcome assessors, participants and a problem with the randomization, however, it is not possible to state in what direction the bias goes. Furthermore, the study primary outcome is objectively measured (hence, only unclear). |

***Kawagoshi 2015***

| **Methods** | **Study design:** Randomized controlled trial  **Study grouping:** Parallel group  **Number of groups:** 2  **Informed consent obtained and ethical approval.:** Written consent was obtained after all patients were informed that they could decide whether or not to participate based on their own freewill and that their privacy would be sufficiently considered. This study was reviewed and approved by the Ethics Committees of our hospital and the Akita University Graduate School of Medicine, and carried out in conformity with the Declaration of Helsinki, 2008 |
| --- | --- |
| **Participants** | **Baseline Characteristics**  Intervention   - *Age*: 74 (8) - *Gender male%*: 83% - *Osteoporosis*: 25% - *Diabetes*: 25% - *Chronic heart failure*: 0% - *Hyper tension*: 8% - *Low back pain*: 17% - *Arthritis*: 8% - *Body mass index*: 21.7 (3.1)   Control   - *Age*: 75 (9) - *Gender male%*: 93 % - *Osteoporosis*: 25% - *Diabetes*: 17% - *Chronic heart failure*: 8% - *Hyper tension*: 17% - *Low back pain*: 8% - *Arthritis*: 8% - *Body mass index*: 22.0 (3.1)   **Included criteria:** The inclusion criteria for this study were: (1) the patient was in stable condition with no infection or exacerbation of COPD for at least the prior 3 months; (2) the patient was able to walk unassisted and operate the device to measure their PA;(3) the patient had no severe and/or unstable cardiac disease, orthopedic disease, or mental disorder that could impair physical activities in daily life.  **Excluded criteria:** -  **Pretreatment:** -  **Possible diagnostic criteria:** Forty-two patients who were diagnosed with stable COPD from mild to very severe stage (GOLD) were enrolled in the present study. Reference: Global Initiative for Chronic Obstructive Lung Disease. Global strategy for the diagnosis, management and prevention of chronic obstructive pulmonary disease-executive summary. Heart, Lung, and Blood Institute, World health Organization;2003. |
| **Interventions** | **Intervention Characteristics**  **Name of intervention:** Pulmonary rehabilitation and feedback from pedometer use  **Duration:** One year  **Short description:** *The patients received the feedback monthly with their average daily PA of the previous month from the PR staff. The patients received this feedback 11 times in the year. The Nakanojo study reported that 8000 steps per a day was correlated with lower limb function [18], and thus the COPD patients in that study were given the goal of taking 8000 steps per a day. The PR staff also gave verbal reinforcement to the patients to increase their PA when they received their PA feedback.*  **Placement of Physical Activity Monitor: Hip**  **Type of Physical Activity Monitor:**  **Feedback frequency:** Monthly  **Control Intervention:** Other training |
| **Outcomes** | *Walking time*   - **Outcome type**: Continuous Outcome - **Reporting**: Fully reported - **Unit of measure**: Minutes - **Direction**: Higher is better - **Data value**: Change from baseline - **Notes**: Extracted from figure 5 with pixel zoomer   *Change in body mass index*   - **Outcome type**: Continuous Outcome - **Reporting**: Fully reported - **Unit of measure**: kg/m2 - **Direction**: Lower is better - **Data value**: Change from baseline   *Change in six-minute walking test*   - **Outcome type**: Continuous Outcome - **Reporting**: Fully reported - **Unit of measure**: Meters - **Direction**: Higher is better - **Data value**: Change from baseline   *Chronic respiratory questionnaire*   - **Outcome type**: Continuous Outcome - **Reporting**: Fully reported - **Direction**: Higher is better - **Data value**: Change from baseline - **Notes**: http://qol.thoracic.org/sections/instruments/ae/pages/crq.html   *Adverse events*   - **Outcome type**: Adverse Event - **Reporting**: Fully reported - **Direction**: Lower is better - **Data value**: Endpoint - **Notes**: In both groups one was hospitalized and one died |
| **Identification** | **Sponsorship source:** NI  **Country:** Japan  **Setting:** Hospital and home  **Comments:** Change in physical activity was extracted from figure 5, with pixel zoomer.  **Authors name:** Atsuyoshi Kawagoshi  **Institution:** Department of Rehabilitation, Akita City Hospital, Akita, Japan  **Email:** acr00009@med.city.akita.akita.jp  **Address:** NI  **Aim:** The objective of the present study was to evaluate the effect of our PR program over a long term with or without the feedback of daily pedometer use by patients with COPD.  **Key conclusion:** These data suggest that low-intensity and home-based PR with the feedback from using pedometer was effective in improving PA, and the improvements of physiological factors were correlated with increased walking time in stable elderly patients with COPD. |
| **Notes** | Paper:  <http://www.sciencedirect.com/science/article/pii/S0954611115000116?via%3Dihub>  The corresponding author has been contacted in vain, about access to the protocol.  The corresponding author was also contacted to obtain full data on table 3. |

Risk of bias table

| **Bias** | **Authors' judgement** | **Support for judgement** |
| --- | --- | --- |
| Bias arising from the randomization process | Unclear risk | Question 1.1 Yes, the allocation sequence was random (flip of a coin)  Question 1.2 No information about concealment of allocation sequence  Question 1.3 There were no baseline imbalances to suggest a problem with the randomization. |
| Bias due to deviations from intended interventions | Unclear risk | Question 2.1 Participants were probably aware of intervention  Question 2.2 Personnel were probably aware of intervention  Question 2.3 No information  Question 2.4 Deviations did probably not affect outcome  Question 2.5 Probably not |
| Bias due to missing outcome data | Low risk | Question 3.2 proportions of missing outcome data and reasons are similar across groups. |
| Bias in measurement of the outcome | Low risk | Question 4.1 Outcome assessors were probably aware of the intervention received by the study participants.  Question 4.2 The assessment were probably not likely to be influenced by the knowledge. |
| Bias in selection of the reported result All outcomes | Unclear risk | Judgement Comment: NI but neither 5.1 or 5.2 Y/PY |
| Overall bias All outcomes | Unclear risk |  |

***Koizumi 2009***

| **Methods** | **Study design:** Randomized controlled trial  **Study grouping:** Parallel group  **Number of groups:** 2  **Ethics:** The Ethical Committee of the Graduate School of Natu­ral Sciences at Nagoya City University approved the study. |
| --- | --- |
| **Participants** | **Baseline Characteristics**  **Included criteria: NI**  **Excluded criteria:** *Gait abnormalities which would affect the measurement of daily activity via an accelerometer; were taking medica­tion prescribed for stroke, hypertension, or hormone replacement therapy; had diagnosed coronary heart dis­ease; or were engaging in an established walking pro­gram for health benefits.*  **Pretreatment: NI** |
| **Interventions** | **Intervention Characteristics**  **Name of intervention:** LIFE  **Duration:** 12 weeks  **Short description:** *The LIFE prescription recommended participants accu­mulate 9000 steps and 30 minutes of moderate intensity physical activity per day… LIFE partici­pants were provided with graphs and scatter plots repre­senting their activity levels for the previous 2-wk period (Figure 1). Based on activity level, recommendations (e.g., increase STEPS by 500 and engage in 3 more min­utes of MPA per day) were provided to each participant. Therefore, every 2 weeks participants could modify their DPA level to better meet the targeted goal.*  **Placement of Physical Activity Monitor:** Hip  **Type of Physical Activity Monitor:** Accelerometer  **Feedback frequency:** Daily  **Control Intervention:** Maintain usual PA |
| **Outcomes** | *Average daily steps*   - **Outcome type**: Continuous Outcome - **Direction**: Higher is better - **Data value**: Endpoint - **Notes**: Extracted from figure 2   *Mod Intensity Activity (min)*   - **Outcome type**: Continuous Outcome - **Reporting**: Fully reported - **Scale**: Minutes - **Direction**: Higher is better - **Data value**: Change from baseline   *Physical capacity 12 Minute Walk*   - **Outcome type**: Continuous Outcome - **Reporting**: Fully reported - **Direction**: Higher is better - **Data value**: Change from baseline |
| **Identification** | **Sponsorship source: NI**  **Country: Japan**  **Setting: Senior centers**  **Comments:**  **Authors name:** Daisuke Koizumi  **Institution:**  Koizumi, Islam, and Takeshima are with the Graduate School of Natural Sciences, Nagoya City University, Nagoya, Japan.  **Aim:** The purpose of this study was to evaluate the effi­cacy of an accelerometer-based lifestyle intervention for community-dwelling older women. It was hypothesized that accelerometer-based feedback and recommenda­tions provided to the intervention group would result in an increase in the quantity and intensity of DPA and a subsequent improvement in cardiorespiratory endurance.  **Primary conclusion:**  Pro­motion of DPA using accelerometers can sig­nificantly improve quantity and quality of daily physical activity as well as cardiorespiratory endurance in older women. |
| **Notes** | Step counts (SD) were extracted from figure |

Risk of bias table

| **Bias** | **Authors' judgement** | **Support for judgement** |
| --- | --- | --- |
| Bias arising from the randomization process | High risk | No baseline data are reported... High risk |
| Bias due to deviations from intended interventions | Unclear risk | Question 2.1 Participants were probably aware of intervention  Question 2.2 Personnel were probably aware of intervention  Question 2.3 No information  Question 2.5 Probably no participants were analyzed in the wrong group |
| Bias due to missing outcome data | Unclear risk | No information |
| Bias in measurement of the outcome | Unclear risk | No information |
| Bias in selection of the reported result All outcomes | Unclear risk | No information |
| Overall bias All outcomes | High risk | Due to the missing baseline data we rate this study overall as high risk |

***Kolt 2012***

| **Methods** | **Study design:** Randomized controlled trial  **Study grouping:** Parallel group  **Number of groups:** 2  **Informed consent obtained/ethical approval:** A home visit was arranged to obtain written informed consent, assess baseline measures, and arrange an appointment with their physician for commencement of the intervention. In addition, participants were given a calendar for recording any falls and injuries. Recruitment took place between July 2006 and December 2007.The trial protocol was approved by the New Zealand Ministry of Health’s Health and Disability Ethics Committee in 2005. |
| --- | --- |
| **Participants** | **Baseline Characteristics**  Intervention   - *Total number of participants*: 165 - *Age*: 74.3 (6.2) - *Gender, male%*: 44.2% - *Hypertension*: 34.4%   Control   - *Total number of participants*: 165 - *Age*: 73.9 (5.9) - *Gender, male%*: 47.9% - *Hypertension*: 40%   **Included criteria:** Inclusion criteria included an age of 65 years or older, ability to communicate in English, ability to give informed consent, residing in the community, ability to walk, and freedom from health conditions that contraindicate participation in physical activity.  **Excluded criteria:** Exclusion criteria included visual impairment that would make it impossible to read a pedometer screen. Physicians screened a list of all their patients for eligibility rst using the 65 years and older criterion, and then excluding only those within this age range who did not meet the above criteria.  **Pretreatment:** |
| **Interventions** | **Intervention Characteristics**  Intervention  **Name of intervention:** Green Prescription  **Duration:** One year  **Short description:** *Goal setting based on steps was used as a main component of this intervention, and participants were encouraged to use their pedometer to monitor steps taken throughout the day. Goals were set by participants as part of their interaction with the physical activity counselor, in which they received guidance on how to set relevant goals based on identified barriers and on factors that enable increasing activity.*  **Placement of Physical Activity Monitor:** Hip  **Type of Physical Activity Monitor:** Pedometer  **Feedback frequency:** Daily  **Control Intervention:** Advice group |
| **Outcomes** | *Total walking activity*   - **Outcome type**: Continuous Outcome - **Reporting**: Fully reported - **Unit of measure**: min/week - **Direction**: Higher is better - **Data value**: Change from baseline   *Minutes in MVPA*   - **Outcome type**: Continuous Outcome - **Reporting**: Fully reported - **Unit of measure**: minutes - **Direction**: Higher is better - **Data value**: Change from baseline   *Change in body mass index*   - **Outcome type**: Continuous Outcome - **Reporting**: Fully reported - **Unit of measure**: kg/m2 - **Direction**: Lower is better - **Data value**: Change from baseline   *SF-36*   - **Outcome type**: Continuous Outcome - **Reporting**: Fully reported - **Direction**: Higher is better - **Data value**: Change from baseline |
| **Identification** | **Sponsorship source:** Funding support: The Health Research Council of New Zealand(05/279R) and Sport and Recreation New Zealand funded this trial.  **Country:** Australia  **Setting:** Primary care physicians from 10 general practices in Auckland, New Zealand.  **Comments:** Trial registration number: Australia and New Zealand Clinical Trial Register ACTRN012606000023550  **Authors name:** Gregory S. Kolt, PhD  **Institution:** School of Science and Health, University of Western Sydney, Australia  **Email:** g.kolt@uws.edu.au  **Address:** University of Western Sydney School of Science and Health Locked Bag 1797Penrith, NSW 2751Australia  **Aim:** Based on an overall goal of improving this national scripting program, the Healthy Steps trial reported here had the aim of comparing the effectiveness of a pedometer-based Green Prescription with that of the proven time-based (standard) Green Prescription.9,10 The trial also sought to investigate the effects of the interventions on blood pressure, body mass index (BMI), functional status, and health-related quality of life.  **Primary conclusion:** Pedometer use resulted in a greater increase in leisure walking without any impact on overall activity level. All participants increased physical activity, and on average, their blood pressure decreased over 12 months, although the clinical relevance is unknown |
| **Notes** | Paper: <https://www.ncbi.nlm.nih.gov/pmc/articles/PMC3354969/>  Trial protocol: <https://bmcpublichealth.biomedcentral.com/articles/10.1186/1471-2458-9-404>  The first author has been contacted about SF36 scores.  SF36 physical functioning score used as HRQoL. |

Risk of bias table

| **Bias** | **Authors' judgement** | **Support for judgement** |
| --- | --- | --- |
| Bias arising from the randomization process | Low risk | Question 1.1 Yes, the allocation sequence was random  Question 1.2 Yes, the allocation sequence was concealed  Question 1.3 There were no baseline imbalances to suggest a problem with the randomization. |
| Bias due to deviations from intended interventions | Low risk | Question 2.1 Participants were probably aware of intervention  Question 2.2 Personnel were probably aware of intervention  Question 2.3 Probably no deviations from indented interventions  Question 2.4 Deviations did probably not affect outcome  Question 2.5 Probably not  Question 2.6 No information |
| Bias due to missing outcome data | Unclear risk | Judgement Comment: Number of included subjects in analysis are not available. |
| Bias in measurement of the outcome | Low risk | Question 4.1 Outcome assessors were probably not aware of intervention |
| Bias in selection of the reported result All outcomes | High risk | Judgement Comment: 3.1 PY Selective outcome reporting on Sf36 scores as only 4 domains have been reported in the final paper. |
| Bias in selection of the reported result Changes in self- reported outcomes | High risk | Judgement Comment: 3.1 PY Selective outcome reporting on Sf36 scores as only 4 domains have been reported in the final paper. |
| Overall bias All outcomes | Low risk | Judgement Comment: Selective outcome reporting on HRQoL All other outcomes are "low" |
| Overall bias Changes in self- reported outcomes | High risk | Judgement Comment: Selective outcome reporting on HRQoL All other outcomes are "low" |

***Lee 2007***

| **Methods** | **Study design:** Randomized controlled trial  **Study grouping:** Parallel group  **Ethics:** The trial was granted ethical approval by the University of Nottingham Medical School Ethics Committee (Study reference number: D/6/2003). |
| --- | --- |
| **Participants** | **Baseline Characteristics**  Intervention   - *Age*: 71.3 (6.4) - *Male %*: 62.7 - *Body mass index*: 25.4 (3.8)   Control   - *Age*: 71.3 (5.7) - *Male %*: 54.0 - *Body mass index*: 25.31 (3.5)   **Included criteria:** The criteria for inclusion in the trial were being resident in a local township, aged 60 years and over and with a resting systolic blood pressure between 140 mmHg and 179 mmHg and thereby classified as having mild to moderate hypertension. |
| **Interventions** | **Intervention Characteristics**  **Name of intervention: -**  **Duration: 6 months**  **Short description:** *The content and timing of subsequent visits were aimed to motivate intervention group participants to walk regularly by increasing the frequency and time spent walking. A pedometer, walking log and advice about regular walking and the use of the pedometer were provided to facilitate participants' initial regular walking. Advice about regular walking was based on established physical activity guidelines.*  **Placement of Physical Activity Monitor:** Hip  **Type of Physical Activity Monitor:** Pedometer  **Feedback frequency:** Daily  **Control Intervention:** Usual Care |
| **Outcomes** | *Walking more*   - **Outcome type**: Standard Mean Difference calculated on: dichotomous outcome - **Reporting**: Fully reported - **Unit of measure**: Interviews and categories - **Direction**: Higher is better - **Data value**: Change from baseline   *Adverse events*   - **Outcome type**: Dichotomous Outcome - **Reporting**: Fully reported - **Direction**: Lower is better - **Data value**: Change from baseline |
| **Identification** | **Sponsorship source:** NI  **Country:** Taiwan  **Setting:** The study was carried out in a rural area of Taiwan  **Comments:**  **Authors name:** Ling-Ling Lee  **Institution:**  **Email:** E-mail addresses: lllee@tccn.edu.tw (L.-L. Lee), tony.arthur@nottingham.ac.uk (A. Arthur), mark.avis@nottingham.ac.uk (M. Avis).  **Address:**  **Aim:** The current study aimed to test whether a community-based walking intervention based on self-efficacy theory could lower systolic blood pressure among older people with mild to moderate hypertension.  **Primary conclusion:** Among hypertensive older people, a six-month community-based walking intervention was effective in increasing their exercise self-efficacy and reducing systolic blood pressure. |
| **Notes** | SMD and SE are calculated by the method described in chapter 9.4.6 of the Cochrane Handbook by Chinn et al. 2000 |

Risk of bias table

| **Bias** | **Authors' judgement** | **Support for judgement** |
| --- | --- | --- |
| Bias arising from the randomization process | Low risk | Question 1.1 Yes, the allocation sequence was random  Question 1.2 Yes, the allocation sequence was concealed  Question 1.3 There were no baseline imbalances to suggest a problem with the randomization. |
| Bias due to deviations from intended interventions | Low risk | Question 2.1 Participants were probably aware of intervention  Question 2.2 Personnel were probably aware of intervention  Question 2.3 Probably no deviations from intended interventions |
| Bias due to missing outcome data | Unclear risk | No information |
| Bias in measurement of the outcome | Unclear risk | Question 4.1 Outcome assessors were probably aware of intervention  Question 4.2 The assessment of the outcome were not likely to be affected by knowledge on the intervention. |
| Bias in selection of the reported result All outcomes | Low risk | Judgement Comment: 5.1 PN5.2 PN |
| Overall bias All outcomes | Low risk |  |

***McLellan 2018***

| **Methods** | **Study design:**  **Study grouping:** Parallel group  **Ethics:** Consent was obtained from all participants (IRB # E2014:062). |
| --- | --- |
| **Participants** | **Baseline Characteristics**  Overall   - *Age*: 72.2(6.3) - *Male %*: 30% - *Body mass index*: 27.9(4.0) - *% university degree*: 65%   **Excluded criteria:** Exclusion criteria included the previous participation in training on exercise intensity using a heart rate monitor (e.g., cardiac rehabilitation program) and using a walking aid. |
| **Interventions** | **Intervention Characteristics**  **Name of intervention: -**  **Duration: 6 weeks**  **Short description:** *The goal of the 6-week program in the intervention group was to walk 150 min per week, reaching at least moderate intensity in bouts of 10 min or more, while using a validated pedometer (StepsCount Piezo®Rx) indicating the walking cadence needed to reach such intensity. After a participant completed one bout of at least 10 minutes the minimum required intensity, a star was shown on the pedometer display. Thus, the goal was to cumulate a minimum of 15 stars in a week.*  **Placement of Physical Activity Monitor:** Hip  **Type of Physical Activity Monitor:** Pedometer  **Feedback frequency:** Daily  **Control Intervention:** Goal setting |
| **Outcomes** | *Average daily steps*   - **Outcome type**: Continuous Outcome   *Minutes MVPA in 10-minute bouts*   - **Outcome type**: Continuous Outcome - **Reporting**: Fully reported - **Direction**: Higher is better - **Data value**: Endpoint |
| **Identification** | **Sponsorship source:** This project was funded by the Manitoba Medical Services Foundation # 8-2013-06RS  **Country:** Canada  **Setting:**  **Comments:** The project was approved by the University of Manitoba Ethics Board E204:062Informed consent was obtained for all participants’8-2013-06RS.  **Authors name:** A. G. McLellan  **Institution:** Faculty of Kinesiology, University of New Brunswick, P.O. Box 4400, Fredericton, NB, Canada  **Email:** Danielle.Bouchard@unb.ca  **Address:**  **Aim:** The first objective of this study was to evaluate whether older adults could improve their ability to correctly identify moderate intensity walking after a 6-week program using a pedometer that provides individualized walking cadence. The second objective was to evaluate change in time spent at a minimum of moderate intensity, in 10-min bouts, per week.  **Primary conclusion:** Results The ability to identify moderate intensity while walking did not significantly improve in neither group (p = 0.530). However, participants in the intervention group increased significantly the time spent at moderate intensity, in 10 min bouts (p 0.01). Discussion A pedometer providing walking cadence to reach moderate intensity is a good tool for increasing time walked at the recommended intensity, but not because participants know more what is considered moderate intensity. |
| **Notes** |  |

Risk of bias table

| **Bias** | **Authors' judgement** | **Support for judgement** |
| --- | --- | --- |
| Bias arising from the randomization process | High risk | Judgement Comment: 1.2 NI1.1 PY 1.3 NI/PY There are no possible way to see the baseline data for demographic values but the fig 2 illustrates that there is a slight tendency to the IV group being more active (however not significant), but in the light of the missing information about the randomization process we evaluate this as High risk. |
| Bias due to deviations from intended interventions | Low risk | Question 2.1 Participants were probably aware of intervention  Question 2.2 Personnel were probably aware of intervention  Question 2.3 Probably no deviations from intended interventions  Question 2.4 Deviations did probably not affect outcome  Question 2.5 Probably not |
| Bias due to missing outcome data | Low risk | Question 3.1 Outcome data were probably available for all participants. |
| Bias in measurement of the outcome | Low risk | Question 4.1 Outcome assessors were probably aware of the intervention received by the study participants.  Question 4.2 The assessment were probably not likely to be influenced by the knowledge. |
| Bias in selection of the reported result All outcomes | Unclear risk | Judgement Comment: 5.1 NI 5.2 NI |
| Overall bias All outcomes | High risk |  |

***McMurdo 2010***

| **Methods** | **Study design:** Randomized controlled trial  **Study grouping:** Parallel group  **Number of groups:** Participants were allocated at random to one of three groups: no intervention(control), a pedometer plus a BCI (pedometer plus group), and only a BCI (BCI group). The control group was not used in this systematic review.  **Informed consent obtained, ethical approval:** The Tayside Committee on Medical Research Ethics approved the study (REC 07/S1402/33), which was conducted in accordance with the Declaration of Helsinki. Written informed consent was obtained from each participant. |
| --- | --- |
| **Participants** | **Baseline Characteristics**  Intervention   - *Age, mean(SD)*: 77.1 (4.9) - *Married, %*: 38%   Control   - *Age, mean(SD)*: 77.6 (5.4) - *Married, %*: 50%   **Included criteria:** Eligibility criteria included community-dwelling, aged 70 and older, and considered inactive (no participation in moderate-intensity physical activity of at least 30 minutes 5 days per week or at least 20 minutes of continuous vigorous-intensity physical activity 3 or more times a week)  **Excluded criteria:** Participants were excluded if they lived in an institution, were housebound or wheelchair bound (thus unable to increase outdoor walking), had moderate to severe cognitive impairment (Mini-Mental State Examination (MMSE)score o18) precluding informed consent, or had significant visual impairment and so were unable to read the pedometer count screen.  **Pretreatment:** |
| **Interventions** | **Intervention Characteristics**  **Name of intervention:** Behaviour change intervention  **Duration:** 6 months  **Short description:** *The average daily pedometer step count or average daily minutes walked outdoors from at least 3 days (at baseline before the intervention) was used to set a target of achieving 20% increase in step counts or minutes walked during the first month. If participants succeeded in meeting their target, it was increased a further 20% at the end of the first and second months. If targets were not being met, the target remained unchanged and was reviewed the following month.*  **Placement of Physical Activity Monitor:** Hip  **Type of Physical Activity Monitor:** Accelerometer  **Feedback frequency:** Daily  **Control Intervention: Goal setting** |
| **Outcomes** | *Accelerometry vector maximum, mean (SD)*   - **Outcome type**: Continuous Outcome - **Reporting**: Fully reported - **Direction**: Higher is better - **Data value**: Endpoint - **Notes**: SD calculated from CI and data extracted with pixelzoomer   *Adverse events*   - **Outcome type**: Adverse Event - **Reporting**: Partially reported - **Direction**: Lower is better - **Data value**: Endpoint - **Notes**: Extracted from the flow chart Adverse events include: ill health and mortality |
| **Identification** | **Sponsorship source:** The trial was funded by Scottish Executive Health Department Grant CZH/4/463. The funder had no role in theconduct, analysis or interpretation of the data.  **Country:** United Kingdom  **Setting:**  **Comments:**  **Authors name:** Marion E. T. McMurdo  **Institution:** Division of Medical Sciences, Ninewells Hospital and Medical School  **Email:** m.e.t.mcmurdo@dundee.ac.uk  **Address:** Ageing and Health,Division of Medical Sciences, Ninewells Hospital and Medical School,Dundee DD1 9SY, United Kingdom.  **Aim:** The hypothesiswas that the provision of a pedometer with a BCI wouldconfer an advantage over a BCI alone in objectively increasingphysical activity levels in sedentary older women.Women were specifically targeted, because inactivity is an established modifiable predictor of functional decline inolder women.  **Established modifiable predictor of functional decline in older women:**  **Primary conclusion:** Provision of a pedometer yielded no additional benefit inphysical activity, but may have motivated participants toremain in the trial. |
| **Notes** | Paper: <http://onlinelibrary.wiley.com/doi/10.1111/j.1532-5415.2010.03127.x/full>  Protocol: <http://www.isrctn.com/ISRCTN26786857>  The change in physical activity was extracted with *pixelzoom* software. |

Risk of bias table

| **Bias** | **Authors' judgement** | **Support for judgement** |
| --- | --- | --- |
| Bias arising from the randomization process | Low risk | Question 1.1 Yes, the allocation sequence was random  Question 1.2 Yes, the allocation sequence was concealed  Question 1.3 There were no baseline imbalances to suggest a problem with the randomization. |
| Bias due to deviations from intended interventions | Low risk | Question 2.1 Participants were probably aware of intervention  Question 2.2 Personnel were probably not aware of intervention  Question 2.3 Probably no deviations from intended interventions  Question 2.4 Deviations did probably not affect outcome  Question 2.5 Probably not |
| Bias due to missing outcome data | High risk | Judgement Comment: 3.1 N3.2 N3.3 N (Not 90% data available) |
| Bias in measurement of the outcome | Low risk | Question 4.1 Outcome assessors were probably aware of the intervention received by the study participants.  Question 4.2 The assessment were probably not likely to be influenced by the knowledge. |
| Bias in selection of the reported result All outcomes | High risk | Judgement Comment: High risk of selective outcome reporting as change scores are only reported at 3 months. The authors conclude that there was a significant difference, but it only exists at 3 months. The long term (end of intervention) should have been used to conclude. |
| Overall bias All outcomes | High risk |  |

***Merom 2016***

| **Methods** | **Study design:** Randomized controlled trial  **Study grouping:** Parallel group  **Number of groups:** 2, 1) Dance (control) and 2) walking (intervention)  **Ethics and consent:** The study protocol wasapproved by the Human Research Ethics Committee (ref: 9987)University of Western Sydney. The trial was registered a day afterthe first participant was enrolled (retrospective registration)with the Australian and New Zealand Clinical Trials Register(ACTRN12613000782730). |
| --- | --- |
| **Participants** | **Baseline Characteristics**  Intervention   - *Age 60-69*: 61.5% - *Age 70-74*: 18.0% - *Age 75+*: 20.5% - *Gender, %male*: 15% - *Educated from University*: 23.1% - *Age mean (SD) provided by the author*: 78.1 (8.5)   Control   - *Age 60-69*: 50% - *Age 70-74*: 25% - *Age 75+*: 25% - *Gender, %male*: 15.4% - *Educated from University*: 35.0% - *Age mean (SD) provided by the author*: 78.7 (8.5)   Overall   - *Age mean (SD) provided by the author*: 78.1 (8.5)   **Included criteria:** Eligible participants had to be ≥60 years of age and be able to walk unaided for at least 50 m. Participants also had to have GP clearance if they suffered from an unstable or chronic condition limiting their participation in regular exercise (e.g. unstable ischemic heart condition, hypertension, and debilitating arthritis).  **Excluded criteria:** Participants were excluded if they had significant cognitive impairment determined by 21 points on the TelephoneInterview of Cognitive Status (TICS), which is the telephonemodification of the Mini-Mental Cognitive Status Examination(MMSE; equivalent to 24)  **Pretreatment:** - |
| **Interventions** | **Intervention Characteristics**  **Name of intervention:** Step-by-Step program  **Duration: 8 months**  **Short description:** *In between the group sessions, participants were encouraged to walk in their residential areas alone or with others and to reach a step target. A progressive goal was provided every 3 months but adherence to the goals was not tracked, in line with the Step-by-Step program effectiveness trial.*  **Placement of Physical Activity Monitor:** Hip  **Type of Physical Activity Monitor:** Pedometer  **Feedback frequency:** Daily  **Control Intervention:** Other training. |
| **Outcomes** | *Six minute walking test*   - **Outcome type**: Continuous Outcome - **Reporting**: Partially reported - **Unit of measure**: Meters - **Direction**: Higher is better - **Data value**: Endpoint - **Notes**: SDs missing   *Adverse events*   - **Outcome type**: AdverseEvent - **Reporting**: Fully reported - **Direction**: Lower is better - **Data value**: Endpoint |
| **Identification** | **Sponsorship source:** IRT Research Foundation  **Country:** Australia  **Setting:** five suburbs around Sydney. The selection of the suburbs was based on (i) availability of an accessible dance studio (i.e., walking – distance from public transport); (ii) dance studio space at least 10 m long; (iii) owner is a ballroom teacher; (iv) owner agreed to volunteer the studio space for the measurements without cost.  **Comments:** -  **Authors name:** Dafna Merom  **Institution:** School of Science and Health, Western Sydney University, Penrith, NSW, Australia,  **Email:** d.merom@westernsydney.edu.au  **Address:** -  **Aim:** We therefore conducted the first randomized controlled trial ofa dance-based intervention, which aimed to compare (i) changesin age-sensitive cognitive domains between two groups of olderadults (60+) randomly assigned to either ballroom dancing(representing a complex multi-dimensional physical activity) orwalking (representing the simplest, functional, and most accessible physical activity in old age); (ii) changes in exercise capacityand sensorimotor capabilities between the two groups, andwhether these changes were associated with cognitive changes.  **Primary conclusion:** The superior potential of dance over walking on executive functions ofcognitively healthy and active older adults was not supported. Dance improved one ofthe cognitive domains (spatial memory) important for learning dance. Controlled trialstargeting inactive older adults and of a higher dose may produce stronger effects, particularlyfor novice dancers. |
| **Notes** | Paper: <https://www.ncbi.nlm.nih.gov/pmc/articles/PMC4761858/>  Trial registration: <https://www.anzctr.org.au/Trial/Registration/TrialReview.aspx?id=364243>  Dr. Merom has been contacted about the missing SDs in table 5 and the missing mean (SD) age in baseline characteristics.  6MWT SD has been imputed from Pelssers 2013. |

Risk of bias table

| **Bias** | **Authors' judgement** | **Support for judgement** |
| --- | --- | --- |
| Bias arising from the randomization process | Low risk | Question 1.1 Yes, the allocation sequence was random  Question 1.2 Yes, the allocation sequence was concealed  Question 1.3 There were no baseline imbalances to suggest a problem with the randomization. |
| Bias due to deviations from intended interventions | High risk | Judgement Comment: According to figure 1, some participants were allowed to switch group. This could affect the outcome |
| Bias due to missing outcome data | High risk | Judgement Comment: High drop out rate and "treatment received" analysis. 3.1 N3.2 N3.3 N |
| Bias in measurement of the outcome | Low risk | Question 4.1 Outcome assessors were probably aware of the intervention received by the study participants.  Question 4.2 The assessment were probably not likely to be influenced by the knowledge. |
| Bias in selection of the reported result All outcomes | High risk | Judgement Comment: The 6MWT is not described in the trial register. |
| Overall bias All outcomes | High risk |  |

***Mutrie 2012***

| **Methods** | **Study design:** Randomized controlled trial  **Study grouping:** Parallel group  **Ethical approval:** All procedures were approved byappropriate NHS (reference number: 08/S0701/121)and University Ethics Committees (reference number:080855). Trial registration: ISRCTN70658148  **Number of groups:** 2 |
| --- | --- |
| **Participants** | **Baseline Characteristics**  Intervention   - *Age*: 71.6 (6.0) - *Male %*: 35% - *University degree*: 20% - *Married*: 40%   Control   - *Age*: 70.0 (4.3) - *Male %*: 29% - *University degree*: 48% - *Married*: 38%   **Included criteria:** Inclusion criteria were living independently and not meeting current PA recommendations.  **Excluded criteria:** Exclusion criteria were refusals, unableto walk outside independently, unable to understandthe rationale behind the trial, and exclusion for medicalreasons by GP.  **Pretreatment:** |
| **Interventions** | **Intervention Characteristics**  **Name of intervention:**  **Duration:** 12 weeks  **Short description:** *A 12-week individualized graduated walking programme in the form of especially designed booklet and pedometer was given to participants. We did not use a universal goal (e.g. 10 000steps) but encouraged steps to be added to each person’s own baseline.*  **Placement of Physical Activity Monitor:** Hip  **Type of Physical Activity Monitor:** Pedometer  **Feedback frequency:** Daily  **Control Intervention:** Maintain usual physical activity |
| **Outcomes** | *Daily average Pedometer step counts (NL-1000)*   - **Outcome type**: Continuous Outcome - **Reporting**: Fully reported - **Unit of measure**: Steps per day - **Direction**: Higher is better - **Data value**: Endpoint   *Sedentary time*   - **Outcome type**: Continuous Outcome - **Reporting**: Fully reported - **Unit of measure**: Minutes - **Direction**: Lower is better - **Data value**: Change from baseline - **Notes**: SD calculated   *SF-36 Physical Health Dimension Score*   - **Outcome type**: Continuous Outcome - **Reporting**: Fully reported - **Direction**: Higher is better - **Data value**: Change from baseline   *Adverse events*   - **Outcome type**: AdverseEvent - **Reporting**: Fully reported - **Direction**: Lower is better - **Data value**: Endpoint |
| **Identification** | **Sponsorship source:** Chief Scientist Office [CSO] Scotland(CZH/4/457); NHS Research and Development fromGreater Glasgow and Clyde and the Scottish PrimaryCare Research Network.  **Country:** UK  **Setting:** One general practice in Glasgow, Scotland (UK).  **Comments:**  **Authors name:** Nanette Mutrie  **Institution:** School of Psychological Sciences and Health, University of Strathclyde, 76 Southbrae Drive, Glasgow G13 1PP, UK,  **Email:** nanette.mutrie@ed.ac.uk  **Address:**  **Aim:** To assess the feasibility of a pedometer-based walking programme in combination withphysical activity consultations.  **Primary conclusion:** It is feasible to recruit and retain older adults from primary care and help themincrease walking. A larger trial is necessary to confirm findings and consider cost-effectiveness. |
| **Notes** | Paper with results:  <https://www.ncbi.nlm.nih.gov/pmc/articles/PMC3501246/>  Study design paper:  <https://www.ncbi.nlm.nih.gov/pmc/articles/PMC3050749/>  Head author has been contacted to get full data (without exclusion of the outliers).  Access to end-point data has been obtained. |

Risk of bias table

| **Bias** | **Authors' judgement** | **Support for judgement** |
| --- | --- | --- |
| Bias arising from the randomization process | Low risk | Question 1.1 Yes, the allocation sequence was random  Question 1.2 Yes, the allocation sequence was concealed  Question 1.3 There were no baseline imbalances to suggest a problem with the randomization. |
| Bias due to deviations from intended interventions | Unclear risk | Judgement Comment: 2.1 Y2.2 Y2.3 Ni2.5 PN |
| Bias due to missing outcome data | Low risk | Question 3.1 Outcome data were probably available for all participants. |
| Bias in measurement of the outcome | Low risk | Question 4.1 Outcome assessors were probably aware of the intervention received by the study participants.  Question 4.2 The assessment were probably not likely to be influenced by the knowledge. |
| Bias in selection of the reported result All outcomes | Low risk | Judgement Comment: Both 5.1 and 5.2 PN |
| Overall bias All outcomes | Low risk |  |

***Nishiguchi 2015***

| **Methods** | **Study design:** Randomized controlled trial  **Study grouping:** Parallel group  **Ethics:** The study was conducted in accordance with the guidelines of the Declaration of Helsinki, and the ethics committee of the Kyoto University Graduate School of Medicine reviewed and approved the study protocol. The trial registration number is JMA-IIA00108. |
| --- | --- |
| **Participants** | **Baseline Characteristics**  Intervention   - *Age*: 73.0 (4.8) - *Male %*: 54.2% - *Body Mass index*: 21.0 (2.4) - *Education years*: 12.2 (2.2)   Control   - *Age*: 73.5 (5.6) - *Male %*: 54.2% - *Body mass index*: 21.2 (2.9) - *Education years*: 13.0 (2.5)   **Included criteria:** Independently community-living individuals aged 60 and older who were willing to participate in group exercise classes for at least 3 months were recruited from the Kyoto City Silver Human Resources Center, Japan.  **Excluded criteria:** Independently community-living individuals aged 60 andolder who were willing to participate in group exercise classes for at least 3 months were recruited from the Kyoto City Silver Human Resources Center, Japan.  **Pretreatment:** |
| **Interventions** | **Intervention Characteristics**  **Name of intervention: -**  **Duration:** 12 weeks  **Short description:** *Participants were instructed to increase the number of daily steps by15% each month and to record the number of steps taken by the end of each day on a calendar. At the end of every month, a sheet was given to each participant to collect brief feedback about the month’s exercises and to provide reminders to record the exercises. The feedback responses were used to assist in setting the number of daily steps assigned for the next month.*  **Placement of Physical Activity Monitor:** Hip  **Type of Physical Activity Monitor:** Pedometer  **Feedback frequency:** Daily  **Control Intervention:** Maintain usual physical activity |
| **Outcomes** | *Average daily steps*   - **Outcome type**: Continuous Outcome - **Reporting**: Fully reported - **Unit of measure**: Steps per day - **Direction**: Higher is better - **Data value**: Endpoint |
| **Identification** | **Sponsorship source:**  **Country:** Japan  **Setting:** Kyoto  **Comments:**  **Authors name:** Shu Nishiguchi, PT, MSc  **Institution:** Department of Physical Therapy, Human Health Sciences, Graduate School of Medicine, Kyoto University  **Email:** m-yamada@human.tsukuba.ac.jp  **Address:**  **Aim:** To investigate whether a 12-week physical and cognitive exercise program can improve cognitive function and brain activation efficiency in community dwelling older adults.  **Primary conclusion:** A 12-week physical and cognitive exercise program can improve the efficiency of brain activation during cognitive tasks in older adults, which is associated with improvements in memory and executive function. |
| **Notes** | Paper: <http://onlinelibrary.wiley.com/doi/10.1111/jgs.13481/full>  Trial register: <https://dbcentre3.jmacct.med.or.jp/jmactr/App/JMACTRS06/JMACTRS06.aspx?seqno=3023> |

Risk of bias table

| **Bias** | **Authors' judgement** | **Support for judgement** |
| --- | --- | --- |
| Bias arising from the randomization process | Low risk | Question 1.1 Yes, the allocation sequence was random (flip of a coin)  Question 1.2 No information about concealment of allocation sequence  Question 1.3 There were no baseline imbalances to suggest a problem with the randomization. |
| Bias due to deviations from intended interventions | Low risk | Question 2.1 Participants were probably aware of intervention  Question 2.2 Personnel were probably aware of intervention  Question 2.3 Probably no deviations from intended interventions  Question 2.5 Probably not |
| Bias due to missing outcome data | Low risk | Question 3.1 Outcome data were probably available for all participants. |
| Bias in measurement of the outcome | Low risk | Quote: "Physical therapists administered the physical function tests, and occupational therapists administered the cognitive function tests; therapists were blinded to group allocation." |
| Bias in selection of the reported result All outcomes | Unclear risk | No information |
| Overall bias All outcomes | Unclear risk |  |

***Nolan 2017***

| **Methods** | **Study design:** Randomized controlled trial  **Study grouping:** Parallel group  **Ethics:** All participants provided written informed consent. The trial protocol was preregistered with clinicaltrials.gov(NCT01719822) and approved by the West London Research Ethics Committee(reference 11/LO/1021).  **Number of groups:** 2 |
| --- | --- |
| **Participants** | **Baseline Characteristics**  Intervention (pedometer and PR)   - *Age*: 69 (9) - *Male %*: 74% - *BMI*: 28.7 (6.6) - *Walking aid*: 90.8%   Control (PR)   - *Age*: 68 (8) - *Male %*: 71% - *BMI*: 27.6 (4.7) - *Walking aid*: 88.2 %   Overall   - *Age*: 68 (9) - *Male %*: 72% - *BMI*: 28.1 (5.8) - *Walking aid*: 89.5%   **Included criteria:** Eligible participants were at least 35 yearsof age, had a physician’s diagnosis ofCOPD consistent with the Global Initiativefor Chronic Obstructive Lung Disease(GOLD) criteria (13), had a MedicalResearch Council dyspnea scale scoregreater than or equal to 2, and consented to undergo supervised PR.  **Excluded criteria:** Exclusioncriteria included contraindications toexercise (e.g., significant cardiovascularcomorbidities) or participants choosing acommunity PR site without access tospecialist exercise equipment.  **Pretreatment:** |
| **Interventions** | **Intervention Characteristics**  **Name of intervention: -**  **Duration:** 8 weeks  **Short description:** *The additional intervention was provision of a pedometer (Yamax Digi-walker CW700; Yamax, Bridgnorth, UK), an individualized daily pedometer step-count target (with weekly review for 8 wk), and a step-count diary provided during the PR program and the following 6 months. During PR, the daily pedometer step-count target was an increase of 5% on the preceding week’s average daily pedometer step count, with the first week’s target derived from the baseline pre-PR assessment (e.g., 250 additional steps from a mean daily step count of 5,000).*  **Placement of Physical Activity Monitor:** Hip  **Type of Physical Activity Monitor:** Pedometer  **Feedback frequency:** Daily  **Control Intervention:** Usual care |
| **Outcomes** | *Pedometer step counts*   - **Outcome type**: Continuous Outcome - **Reporting**: Fully reported - **Unit of measure**: Steps per day - **Direction**: Higher is better - **Data value**: Change from baseline   *Time spend above 3 METS*   - **Outcome type**: Continuous Outcome - **Reporting**: Fully reported - **Unit of measure**: Minutes/day - **Direction**: Higher is better - **Data value**: Change from baseline - **Notes**: mean=median and SD = IQR/1.35.   *Adverse events*   - **Outcome type**: AdverseEvent - **Reporting**: Fully reported - **Direction**: Lower is better - **Data value**: Endpoint |
| **Identification** | **Sponsorship source:**  **Country:** UK  **Setting:**  **Comments:**  **Authors name:** Claire M. Nolan  **Institution:** National Institute for Health Research Respiratory Biomedical Research Unit, Royal Brompton Harefield NHS Foundation Trust, Harefield, United Kingdom  **Email:** c.nolan15@imperial.ac.uk  **Address:** Harefield Hospital, Middlesex UB9 6JH, UK.  **Aim:** Todeterminewhether using pedometers as anadjunct toPRcan enhance timespent inat leastmoderate-intensityphysical activity (timeexpending>3 metabolic equivalents [METs]) by people with COPD.  **Primary conclusion:** Pedometer-directed step-count targets during anoutpatient PR program did not enhance moderate-intensity physicalactivity levels in people with COPD. |
| **Notes** | Paper:  <https://www.ncbi.nlm.nih.gov/pmc/articles/PMC5443901/>  Clinical trials: <https://clinicaltrials.gov/ct2/show/NCT01719822>  First author has been contacted about full data-set and end-point data has been accessed.  CRQ used and end-point scores calculated from baseline and change scores. |

Risk of bias table

| **Bias** | **Authors' judgement** | **Support for judgement** |
| --- | --- | --- |
| Bias arising from the randomization process | Low risk | Question 1.1 Yes, the allocation sequence was random  Question 1.2 Yes, the allocation sequence was concealed  Question 1.3 There were no baseline imbalances to suggest a problem with the randomization. |
| Bias due to deviations from intended interventions | Unclear risk | Question 2.1 Participants were probably aware of intervention  Question 2.2 Personnel were probably aware of intervention  Question 2.3 No information  Question 2.4 Deviations did probably not affect outcome |
| Bias due to missing outcome data | Low risk | Question 3.1 No, data were not available for all participants randomized.  Question 3.2 Yes there is evidence that the results are robust to the presence of missing data. |
| Bias in measurement of the outcome | Low risk | Question 4.1 Outcome assessors were probably not aware of intervention |
| Bias in selection of the reported result All outcomes | High risk | Judgement Comment: 5.1 Y (SF-36 missing in paper) 5.2 N |
| Bias in selection of the reported result Changes in self- reported outcomes | High risk | Judgement Comment: According to the study protocol, secondary outcomes include: Short Form 36 item questionaire SF-36 [ Time Frame: 8 weeks ], however it has not been reported in the final paper. |
| Overall bias All outcomes | Unclear risk | Judgement Comment: SF 36 HIGH Rest is unclear |
| Overall bias Changes in self- reported outcomes | High risk | Judgement Comment: SF 36 HIGH Rest is unclear |

***Peel 2016***

| **Methods** | **Study design:** Randomized controlled trial  **Study grouping:** Parallel group  **Number of groups:** 2  **Ethics:** The study was approved by the Human Research Ethics Committees at each site. Patients gave informed written consent to participate; in the event of incapacity to consent, assent for participation was sought from next of kin or carers. |
| --- | --- |
| **Participants** | **Baseline Characteristics**  Intervention   - *Age*: 81 (9) - *Male %*: 39% - *Body mass index*: 25.6 (6.7) - *Walking Without Aids n(%)*: 4 (3) - *Number of Co-morbidities mean (SD)*: 8 (4)   Control   - *Age*: 82 (8) - *Male %*: 45% - *Body mass index*: 24.5 (5.2) - *Walking Without Aids n(%)*: 11 (9) - *Number of Co-morbidities mean (SD)*: 8 (4)   **Included criteria:** Patients admitted to post-acute care rehabilitation who were (1) aged 60 years and older; (2) able to ambulate independently or with supervision/assistance and had a rehabilitation goal to become ambulant within the context of the current admission and (3) expected to have a lengt hof stay of at least two weeks, were eligible to participate.  **Excluded criteria:** Exclusion criteria were those (1) with lower limb amputation; (2) with delirium or agitated dementia, as documented by the geriatric treating team; or (3) not expected to walk within four weeks of admission.  **Pretreatment:** |
| **Interventions** | **Intervention Characteristics**  **Name of intervention: -**  **Duration:** 4 weeks  **Short description:** *Accelerometers were used to monitor patients’ activity in both the intervention and control groups. For the intervention group, accelerometer data was downloaded daily. Feedback was provided to the intervention participant and their therapists of the previous day’s walking time in numerical and graphical form, showing walking time outside therapy sessions compared with walking target. The treating therapist, in consultation with the patient, set mobility goals, including provisional targets for daily walking time. These goals were reviewed weekly and modified, informed by the accelerometer data, to motivate the patient to improve incidental activity levels outside of therapy sessions and reach set targets.*  **Placement of Physical Activity Monitor: Hip**  **Type of Physical Activity Monitor:** Accelerometer  **Feedback frequency:** Daily  **Control Intervention:** Usual care |
| **Outcomes** | *Mean weekly walking time*   - **Outcome type**: Continuous Outcome - **Reporting**: Fully reported - **Unit of measure**: Minutes - **Direction**: Higher is better - **Data value**: Endpoint   *Adverse events*   - **Outcome type**: Adverse Event - **Reporting**: Fully reported - **Unit of measure**: Numbers - **Direction**: Lower is better - **Data value**: Endpoint   *MVPA: Total weekly minutes*   - **Outcome type**: Continuous Outcome - **Reporting**: Fully reported - **Direction**: Higher is better - **Data value**: Endpoint |
| **Identification** | **Sponsorship source:** This work was supported by Australian National Health and Medical Research Council(NHMRC) Grant (APP1007886), https://www.nhmrc.gov.au/, LCG NMP SKP IDC SEK MC  **Country:** Australia  **Setting:** The setting was post-acute care Geriatric Rehabilitation Units or Geriatric Evaluation and Management Units with at least a 40 bed capacity, at three Australian sites  **Comments:**  **Authors name:** Nancye M. Peel  **Institution:** Centre for Research in Geriatric Medicine, The University of Queensland, Brisbane, Australia  **Email:** n.peel@uq.edu.au  **Address:**  **Aim:** The study aimed to test whether activity levels can be increased by the provision of monitored activity data to patients and clinicians in the context of explicit goal setting.  **Primary conclusion:** Daily feedback to patients and therapists using an accelerometer increased walking times during rehabilitation admissions. The results of this study suggest objective monitoring of activity levels could provide clinicians with information on clinically important, mobility related activities to assist goal setting. |
| **Notes** | Paper:  <http://journals.plos.org/plosone/article?id=10.1371/journal.pone.0160906>  Protocol:  <http://journals.plos.org/plosone/article/file?id=10.1371/journal.pone.0160906.s002&type=supplementary> |

Risk of bias table

| **Bias** | **Authors' judgement** | **Support for judgement** |
| --- | --- | --- |
| Bias arising from the randomization process | Low risk | Question 1.1 Yes, the allocation sequence was random  Question 1.2 Yes, the allocation sequence was concealed  Question 1.3 There were no baseline imbalances to suggest a problem with the randomization. |
| Bias due to deviations from intended interventions | Unclear risk | Question 2.1 Participants were probably aware of intervention  Question 2.2 Personnel were probably aware of intervention  Question 2.3 No information  Question 2.4 Deviations did probably not affect outcome |
| Bias due to missing outcome data | Low risk | Question 3.1 Outcome data were probably available for all participants. |
| Bias in measurement of the outcome | Low risk | Question 4.1 Outcome assessors were probably not aware of intervention |
| Bias in selection of the reported result All outcomes | Low risk | Judgement Comment: 5.1 N5.2 N |
| Overall bias All outcomes | Low risk |  |

***Pelssers 2013***

| **Methods** | **Study design:** Randomized controlled trial  **Study grouping:** Parallel group  **Number of groups:** 2  **Ethics:** Ethical approval for this study was obtained from the ethical committee of the biomedical sciences group of the KU Leuven. |
| --- | --- |
| **Participants** | **Baseline Characteristics**  Intervention   - *Age*: 69.40 (7.26) - *Male %*: 30.6% - *Married*: 63.3% - *University college /University*: 10.3%   Control   - *Age*: 70.34 (6.38) - *Male %*: 37.8% - *Married*: 73.6% - *University college /University*: 16.5%   **Included criteria:** Older adults (age ≥ 55 yr). |
| **Interventions** | **Intervention Characteristics**  **Name of intervention: -**  **Duration:** 10 weeks  **Short description:** *They received a pedometer and completed the standardized6-min walking test to determine their entry level to the walking intervention. Based on their result, participants received their personal structured walking program. Instructions on the use of the program were provided. Finally, they filled out a questionnaire for the study. In the next 10 weeks, participants completed their personal walking program and attended group walks in the local meeting points*  **Placement of Physical Activity Monitor:** Hip  **Type of Physical Activity Monitor:** Pedometer  **Feedback frequency:** Daily  **Control Intervention:** Advice group |
| **Outcomes** | *Adverse events*   - **Outcome type**: Adverse Event - **Reporting**: Fully reported - **Direction**: Lower is better - **Data value**: Endpoint   *GLTEQ total score*   - **Outcome type**: Continuous Outcome - **Reporting**: Fully reported - **Direction**: Higher is better - **Data value**: Endpoint - **Notes**: Self reported PA   *6MWT*   - **Outcome type**: Continuous Outcome - **Reporting**: Fully reported - **Unit of measure**: Meters - **Direction**: Higher is better - **Data value**: Endpoint   *Physical well being*   - **Outcome type**: Continuous Outcome - **Direction**: Higher is better - **Data value**: Endpoint - **Notes**: Four items of the Marcoen scale for subjective well-being (Marcoen et al.,2002) were used to assess physical well-being. Participants reported on a 7-pointLikert scale ranging from (1) never to (7) always how often they felt as stated ineach item (e.g., “I feel good in my body”). The scale was internally consistent, with Cronbach’s α of .83 at pretest and .84 at posttest.Physical well being used as HRQoL |
| **Identification** | **Sponsorship source:**  **Country:**  **Setting:** Community-based senior organization  **Comments:**  **Authors name:** Johan Pelssers  **Institution:** Dept. of Kinesiology, KU Leuven, Leuven, Belgium.  **Email:**  **Address:** |
| **Notes** | Paper:  <https://www.ncbi.nlm.nih.gov/pubmed/22898451>  The last author (Boen) has been contacted about inclusion/exclusion, BMI, source of funding. |

Risk of bias table

| **Bias** | **Authors' judgement** | **Support for judgement** |
| --- | --- | --- |
| Bias arising from the randomization process | Unclear risk | No information |
| Bias due to deviations from intended interventions | Unclear risk | Question 2.1 Participants were probably aware of intervention  Question 2.2 Personnel were probably aware of intervention  Question 2.3 No information  Question 2.4 Deviations did probably not affect outcome |
| Bias due to missing outcome data | Low risk | Question 3.1 No, data were not available for all participants randomized.  Question 3.2 Yes there is evidence that the results are robust to the presence of missing data. |
| Bias in measurement of the outcome | Unclear risk | Question 4.1 Outcome assessors were probably aware of the intervention received by the study participants.  Question 4.2 No information. |
| Bias in selection of the reported result All outcomes | Unclear risk | Judgement Comment: At least one (5.1 and 5.2) No information but neither Y/PY |
| Overall bias All outcomes | Unclear risk |  |

***Rowley 2017***

| **Methods** | **Study design:** Randomized controlled trial  **Study grouping:** Parallel group  **Number of groups:** 3  **Ethics:** Procedures were approved by the Institutional Review Board (IRB) at a major Midwestern university (IRB Protocol Number 07.02.317). |
| --- | --- |
| **Participants** | **Baseline Characteristics**  Pedometer only   - *Age*: 68.3 (7.1) - *Male*: 21.5% - *College*: 46.4% - *Body mass index*: 30.7 (4.3)   Tailored Internet-mediated pedometer   - *Age*: 67.4 (6.4) - *Male*: 24% - *College*: 40.4% - *Body mass index*: 29.1 (3.6)   Control   - *Age*: 66.1 (4.9) - *Male*: 21.4% - *College*: 57% - *Body mass index*: 29.6 (3.1)   **Included criteria:** Adults aged 55 to 80 years, who were inactive or insufficiently active, definedas achieving less than 7,500 steps per day (Tudor-Locke Bassett, 2004), wererecruited to participate in this study (see Figure 1 for study flow). In addition,participants were included if they had no orthopedic limitations to walking,expressed interest in starting a walking program, and had access to a computerand the Internet.  **Excluded criteria:**  **Pretreatment:** |
| **Interventions** | **Intervention Characteristics**  **Name of intervention:** a) PED and b) TI-PED  **Duration:** 12 weeks  **Short description:** a) *Participants were* *given the goal to increase their daily step count by 10% each week until they met 10,000 stepsper day after which they were instructed to maintain 10,000 steps per day.*  b) *Phase 1,Weeks 1 to 3, was used to provide cognitive understanding of the benefits of PA, education on national recommendations, and self-awareness of current activity levels. Uploaded steps per day were graphically represented for each day for the prior week, and plotted against nationally recommended amounts. Phase 2, Weeks 4 to 12, continued to provide educational information, and now required each individual to, intrinsically, set manageable daily step targets for themselves, such as increasing weekly steps by 10%increments. Pedometer uploading took place at the end of each week. Graphical representations of daily steps were provided along with information on how well they corresponded with intrinsically set goals. At this stage of the program, each individual was either in compliance with set goals (defined as meeting walking step targets 5 out of 7 days) or they were not in compliance. If a participant was in compliance, the user was guided through a series of congratulatory screens and a directive for setting the upcoming week’s PA step goal. If the participant was not in compliance, the user was guided through a series of interactive screens that were designed to collect barriers to accomplishing the goal and then deliver motivational messages tagged and retrieved from a database library.*  **Placement of Physical Activity Monitor:** Hip  **Type of Physical Activity Monitor:** Pedometer  **Feedback frequency: a) daily and b) daily + weekly strategies from website**  **Control Intervention:** Maintain usual physical activity |
| **Outcomes** | *Daily step counts*   - **Outcome type**: Continuous Outcome - **Reporting**: Fully reported - **Unit of measure**: Steps per day - **Direction**: Higher is better - **Data value**: Endpoint |
| **Identification** | **Sponsorship source:** The authors disclosed receipt of the following financial support for the research,authorship, and/or publication of this article: This study was funded by a grant throughthe National Institute on Aging (5K01AG025962).  **Country:** USA  **Setting:** Participants were recruited from the community via mass media announcements, mailed invitations, and recruitment postings.  **Comments:**  **Authors name:** Taylor W. Rowley  **Institution:** Scott J. Strath, Department of Kinesiology, Center for Aging and Translational Research, University of Wisconsin–Milwaukee, Milwaukee, WI 53201, USA.  **Email:** sstrath@uwm.edu  **Address:**  **Aim:** This study determined the effectiveness of an individually tailored, Internet-mediated physical activity (PA) intervention for increasing walking behavior in inactive older adults.  **Primary conclusion:** Individually tailored, Internet-mediated PA interventions are an effective way to significantly increase PA in older adults. |
| **Notes** | Paper:  <http://journals.sagepub.com/doi/abs/10.1177/0733464817735396?url_ver=Z39.88-2003&rfr_id=ori:rid:crossref.org&rfr_dat=cr_pub%3dpubmed>  The corresponding author has been written about access to the protocol. |

Risk of bias table

| **Bias** | **Authors' judgement** | **Support for judgement** |
| --- | --- | --- |
| Bias arising from the randomization process | Unclear risk | Question 1.1 and 1.2 No information about randomization or concealment of allocation sequence  Question 1.3 There were no baseline imbalances to suggest a problem with the randomization. |
| Bias due to deviations from intended interventions | Unclear risk | Question 2.1 Participants were probably aware of intervention  Question 2.2 Personnel were probably aware of intervention  Question 2.3 No information  Question 2.4 Deviations did probably not affect outcome |
| Bias due to missing outcome data | High risk | Outcome data were not available for all participants and no information were available on robustness to missing outcome data. |
| Bias in measurement of the outcome | Low risk | Question 4.1 Outcome assessors were probably aware of intervention  Question 4.2 The assessment of the outcome were not likely to be affected by knowledge on the intervention. |
| Bias in selection of the reported result All outcomes | Unclear risk | Judgement Comment: 5.1 and 5.2 NI |
| Overall bias All outcomes | High risk | Judgement Comment: Randomization and NI about outcome data (and no ITT analysis). |

***Sugden 2008***

| **Methods** | **Study design:** Randomized controlled trial  **Study grouping:** Parallel group  **Number of groups:** 2  **Ethics:** The Tayside Committee on Medical Ethics approved thestudy which was carried out in accordance with the Declarationof Helsinki. Written, informed consent wasobtained. |
| --- | --- |
| **Participants** | **Baseline Characteristics**  Intervention   - *Age*: 76, range 70–86   **Included criteria:** Women aged over 70 years who were insufficiently active or sedentary, i.e. no participation in moderate-intensityphysical activity of at least 30 minutes at least 5 days perweek or at least 20 minutes of continuous vigorous-intensityactivity three or more times a week [14] were includedin the study. Women were asked about their participationin physical exercise and walking at a preliminary phonecall before any visits took place. Women fulfilling physicalactivity recommendations, resident of institutional care,housebound (unable to increase outdoor walking), havingmoderate to severe cognitive impairment (MMSEscore 18) precluding informed consent, having significantvisual impairment and so unable to read pedometercount screen, wheelchair bound or unwilling to participatewere excluded from the study.  **Excluded criteria:**  **Pretreatment:** |
| **Interventions** | **Intervention Characteristics**  **Name of intervention: -**  **Duration:** 12 weeks  **Short description:** *The pedometer group were further randomized to one of three target groups: a 10%, 15% or 20% monthly increase instep count (steps taken per day). The average daily pedometer count over three consecutive days (at baseline before intervention) was taken and used to set a target of achieving a 10%, 15% or 20% increase in steps during the first month. If the participant met her target step count, it was increased again after the first and second months.*  **Placement of Physical Activity Monitor:** Hip  **Type of Physical Activity Monitor:** Pedometer  **Feedback frequency:** Daily  **Control Intervention:** Advice group |
| **Outcomes** | *Change in accelerometry (daily activity count)*   - **Outcome type**: Continuous Outcome - **Reporting**: Fully reported - **Direction**: Higher is better - **Data value**: Endpoint |
| **Identification** | **Sponsorship source:** This study was funded by the Chief Scientist Office, Scottish Executive Health Department grant number CZH/4/310.  **Country:** UK  **Setting:** Primary care  **Comments:**  **Authors name:** Jacqui A Sugden  **Institution:** Section of Ageing Health, Division of Medicine Therapeutics, University of Dundee, Ninewells Hospital Medical School, Dundee, UK,  **Email:** j.a.sugden@dundee.ac.uk  **Address:**  **Aim:** The aim of this pilot study was to evaluate the feasibility of the use of pedometers plus a theory-based intervention to assist sedentary older women to accumulate increasing amounts of physical activity, mainly through walking.  **Primary conclusion:** We have demonstrated that it is feasible to use pedometers and provide theory-based advice to community dwelling sedentary older women to increase physical activity levels and a larger study is planned to investigate this further. |
| **Notes** |  |

Risk of bias table

| **Bias** | **Authors' judgement** | **Support for judgement** |
| --- | --- | --- |
| Bias arising from the randomization process | Unclear risk | Question 1.1 Yes, the allocation sequence was random  Question 1.2 Yes, the allocation sequence was concealed  Question 1.3 There were some baseline imbalances to suggest a problem with the randomization (anxiety) |
| Bias due to deviations from intended interventions | Low risk | Question 2.1 No information and participants were probably aware of intervention  Question 2.2 No information and personel were probably aware of intervention  Question 2.3 Probably not  Question 2.4 Deviations did probably not affect outcome |
| Bias due to missing outcome data | Low risk | Outcome data were not available for all participants but there was evidence to ensure robustness of the results |
| Bias in measurement of the outcome | Low risk | Question 4.1 Outcome assessors were probably aware of intervention  Question 4.2 The assessment of the outcome were not likely to be affected by knowledge on the intervention. |
| Bias in selection of the reported result All outcomes | Unclear risk | No information |
| Overall bias All outcomes | Unclear risk |  |

***Tabak 2014***

| **Methods** | **Study design:** Randomized controlled trial  **Study grouping:** Parallel group  **Number of groups:** 2  **Ethics:** The study was approved by the Medical Ethical Committee Twente and registered in the Netherlands Clinical Trial Register (no. NTR2440). The trial took place between October 2010 and April 2011. |
| --- | --- |
| **Participants** | **Baseline Characteristics**  Intervention   - *Age*: 65.2 (9.0) - *Male %*: 57% - *Body mass index*: 28.4 (7.8)   Control   - *Age*: 67.9 (5.7) - *Male %*: 69% - *Body mass index*: 29.2 (4.7)   **Included criteria:** Patients with a clinical diagnosis of ChronicObstructive Pulmonary Disease were recruited by achest physician or nurse practitioner. Inclusion criteriawere: no infection or exacerbation in the fourweeks prior to measurement; current or formersmoker; able to read and speak Dutch; and internetaccess at home.  **Excluded criteria:** Exclusion criteria were: impairedhand function causing inability to use the application;disorders or progressive disease seriouslyinfluencing daily activities (e.g. amputation); otherdiseases influencing bronchial symptoms and/orlung function (e.g. sarcoidosis); need for regularoxygen therapy (>16 hours per day or pO2 7.2kPa); a history of asthma, and less than six weeksago started training with a physiotherapist.  **Pretreatment:** |
| **Interventions** | **Intervention Characteristics**  **Name of intervention:**  **Duration: 4 weeks**  **Short description:** *The application consisted of two modules: (1) activity coach for ambulant activity registration and feedback and (2) web portal with a symptom diary for self-treatment of exacerbations and an overview of the measured activity levels. The activity coach consisted of a three-dimensional-accelerometer (MTx-W sensor, Xsens Technologies, Enschede, The Netherlands) and a smartphone (HTCP3600/3700)… Participants were asked to try to be active in such a way during the day that the displayed reference line is closely approached. In addition, the users automatically received feedback text messages, for awareness and extra motivation. These messages were based on the difference between the measured activity and the reference line and always consist of (1) a short summary of activity behaviour and (2) advice on how to improve or maintain the activity behaviour. The participant’s measured activity levels were also displayed on the web portal.*  **Placement of Physical Activity Monitor:** Hip  **Type of Physical Activity Monitor:** Accelerometer  **Feedback frequency:** Daily  **Control Intervention:** Usual care |
| **Outcomes** | *Mean steps per day*   - **Outcome type**: Continuous Outcome - **Reporting**: Fully reported - **Unit of measure**: Steps per day - **Direction**: Higher is better - **Data value**: Endpoint |
| **Identification** | **Sponsorship source:** This study was supported by a research grant from TheNetherlands Organization for Health Research andDevelopment (ZonMW).  **Country:** Netherlands  **Setting:**  **Comments:**  **Authors name:** Monique Tabak  **Institution:** Roessingh Research and Development, Telemedicine Group, Enschede, The Netherlands  **Email:** m.tabak@rrd.nl  **Address:** Monique Tabak, MSc, Roessingh Research and Development,Roessinghsbleekweg 33b, PO Box 310, Enschede, 7500 AH,The Netherlands.  **Aim:** First, to investigate the effects of a telerehabilitation intervention on health status and activitylevel of patients with Chronic Obstructive Pulmonary Disease (COPD), compared to usual care. Second,to investigate how patients comply with the intervention and whether compliance is related to treatmentoutcomes.  **Primary conclusion:** This pilot study shows the potential of the telerehabilitation intervention: compliance withthe activity coach was high, which directly related to an improvement in activity levels. |
| **Notes** | Original paper:  <http://journals.sagepub.com/doi/abs/10.1177/0269215513512495?url_ver=Z39.88-2003&rfr_id=ori:rid:crossref.org&rfr_dat=cr_pub%3dpubmed>  First author has been contacted about the study protocol |

Risk of bias table

| **Bias** | **Authors' judgement** | **Support for judgement** |
| --- | --- | --- |
| Bias arising from the randomization process | Unclear risk | Question 1.1 Yes, the allocation sequence was random  Question 1.2 Yes, the allocation sequence was concealed  Question 1.3 There were baseline imbalances to suggest a problem with the randomization. |
| Bias due to deviations from intended interventions | Unclear risk | Question 2.1 Participants were probably aware of intervention  Question 2.2 Personnel were probably aware of intervention  Question 2.3 No information  Question 2.4 Deviations did probably not affect outcome |
| Bias due to missing outcome data | High risk | Judgement Comment: 3.1 Data were not available for all participants.  22% Dropout in intervention group 3.2 N 3.3 N |
| Bias in measurement of the outcome | Low risk | Question 4.1 Outcome assessors were probably aware of the intervention received by the study participants.  Question 4.2 The assessment were probably not likely to be influenced by the knowledge. |
| Bias in selection of the reported result All outcomes | Unclear risk | Judgement Comment: 5.1 NI5.2 NI |
| Overall bias All outcomes | Unclear risk |  |

***Talbot 2003***

| **Methods** | **Study design:** Randomized controlled trial  **Study grouping:** Parallel group  **Ethics:** Participants read and signed an informed consent form approved by the Johns HopkinsUniversity Institutional Review Board. |
| --- | --- |
| **Participants** | **Baseline Characteristics**  Pedometer group   - *Age*: 69.6 (6.7) - *Male*: 23.5% - *Body mass index*: 31.01 (5.9) - *Married %*: 47.1 %   Control group   - *Age*: 70.8 (4.7) - *Male*: 23.5% - *Body mass index*: 232.63 (6.9) - *Married %*: 47.1 %   **Included criteria:** Inclusion criteria included aged 60 and older, pain in one or both knees on mos tdays, difficulty performing at least one functional task becauseof pain, and radiographic evidence of OA. Exclusionarycriteria included current participation in an exerciseresearch study; a medical condition for which exerciseis contraindicated, such as unstable angina pectoris or recent myocardial infarction; and a score of less than 24 onthe Mini-Mental State Examination  **Excluded criteria:**  **Pretreatment:** |
| **Interventions** | **Intervention Characteristics**  **Name of intervention:** Walk group program  **Duration:** 12 weeks  **Short description:** *At the initiation of the program, participants were instructed to wear a pedometer for monitoring their daily steps. The number of daily steps was modified to the individuals’ baseline step count and increased by 10% every 4 weeks. Thus, by the end of 12weeks, participants would be walking 30% above their baseline step count*  **Placement of Physical Activity Monitor:** Hip  **Type of Physical Activity Monitor:** Pedometer  **Feedback frequency:** Daily  **Control Intervention:** Advice group |
| **Outcomes** | *Steps per day*   - **Outcome type**: Continuous Outcome - **Reporting**: Fully reported - **Unit of measure**: Steps/day - **Direction**: Higher is better - **Data value**: Endpoint |
| **Identification** | **Sponsorship source:** Funded by the Fund for Geriatric Medicine and Nursing, Johns Hopkins University, and the Intramural Research Program of the National Institute on Aging.  **Country:** USA  **Setting:** Community located in the Baltimore-Washington area.  **Comments:** Community located in the Baltimore-Washington area.  **Authors name:** Laura A. Talbot  **Institution:** Johns Hopkins University, School of Nursing, Baltimore, Maryland; † Erickson Foundation, Baltimore, Maryland  **Email:** ltalbot@son.jhmi.edu  **Address:**  **Aim:** To investigate whether a pragmatic structured education program with and without pedometer use is effective for promoting physical activity and improving glucose tolerance in those with impaired glucose tolerance (IGT).  **Primary conclusion:** This study suggests that a pragmatic structured education program that incorporates pedometer use is effective for improving glucose tolerance in those with IGT. This result is likely to have important implications for future primary care– based diabetes prevention initiatives. |
| **Notes** | Paper:  <http://onlinelibrary.wiley.com/doi/10.1046/j.1532-5415.2003.51113.x/full>  First author has been contacted about the study protocol |

Risk of bias table

| **Bias** | **Authors' judgement** | **Support for judgement** |
| --- | --- | --- |
| Bias arising from the randomization process | Low risk | Question 1.1 Yes, the allocation sequence was random  Question 1.2 Yes, the allocation sequence was concealed  Question 1.3 No information |
| Bias due to deviations from intended interventions | Unclear risk | Question 2.1 Participants were probably aware of intervention  Question 2.2 Personnel were probably aware of intervention  Question 2.3 No information  Question 2.4 Deviations did probably not affect outcome |
| Bias due to missing outcome data | Low risk | Outcome data were not available for all participants but there is evidence to suggest robustness for missing outcome data. |
| Bias in measurement of the outcome | Low risk | Question 4.1 Outcome assessors were probably aware of intervention  Question 4.2 The assessment of the outcome were not likely to be affected by knowledge on the intervention. |
| Bias in selection of the reported result All outcomes | Unclear risk | No information |
| Overall bias All outcomes | Unclear risk |  |

***Yates 2009***

| **Methods** | **Study design:** Randomized controlled trial  **Study grouping:** Parallel group  **Ethics:** This study was approved by the Leicestershire, Northamptonshire, and Rutland National Health Service Research Ethics Committee in June 2006. |
| --- | --- |
| **Participants** | **Baseline Characteristics**  Intervention with pedometer   - *Age*: 66 (8) - *Male %*: 69% - *Body mass index*: 28.7 (4.8)   Intervention without pedometer   - *Age*: 64 (7) - *Male %*: 69% - *Body mass index*: 29.5 (4.9)   **Included criteria:** Overweight or obese individuals (BMI 25 or 23 kg/m2 for South Asians) with screening-detected IGT (7) were contacted by letter and follow-up telephone call by a member of their screening team and invited to take part in the study. Individuals were recruited into the study within 12 months of their screening visit. As part of the screening programs, all individuals had their physical activity levels assessed by the short version of the international physical activity questionnaire (IPAQ) (8).  **Excluded criteria:** Individuals who reported taking steroids were excluded.  **Pretreatment:** |
| **Interventions** | **Intervention Characteristics**  **Name of intervention:** PREPARE  **Duration:** One year  **Short description:** In addition to a physical activity education. Participants received goal setting and a pedometer.  *As part of the program, participants were provided with a pedometer (SW- 200; Yamax, Tokyo, Japan) and encouraged to set personalized steps-per-day goals based on their baseline ambulatory activity level. Sedentary participants were encouraged to increase their activity levels by at least 3,000 steps per day, equivalent to 30 min of walking (12). Those achieving 6,000 steps per day were encouraged to try to reach at least 9,000 steps per day, an amount that is likely to include 30 min of walking activity in addition to usual daily activity (12). Those achieving 9,000 steps per day were encouraged to at least maintain their current activity levels and were informed that health benefits could be achieved by increasing their activity levels further.*  **Placement of Physical Activity Monitor: Hip**  **Type of Physical Activity Monitor:** Pedometer  **Feedback frequency:** Daily  **Control Intervention:** Goal setting |
| **Outcomes** | *Ambulatory activity*   - **Outcome type**: Continuous Outcome - **Unit of measure**: Steps per day - **Direction**: Higher is better - **Data value**: Change from baseline   *Total MVPA*   - **Outcome type**: Continuous Outcome - **Reporting**: Fully reported - **Unit of measure**: Minutes - **Direction**: Higher is better - **Data value**: Change from baseline   *Adverse events*   - **Outcome type**: AdverseEvent - **Reporting**: Fully reported - **Direction**: Lower is better - **Data value**: Endpoint |
| **Identification** | **Sponsorship source:** This trial was funded by a grant from Diabetes UK.  **Country:** UK  **Setting:** University Hospitals of Leicester  **Comments:**  **Authors name:** THOMAS YATES  **Institution:** Department of Cardiovascular Sciences, University of Leicester, U.K  **Email:** ty20@le.ac.uk.  **Address:**  **Aim:** To investigate whether a pragmatic structured education program with and without pedometer use is effective for promoting physical activity and improving glucose tolerance in those with impaired glucose tolerance (IGT).  **Primary conclusion:** This study suggests that a pragmatic structured education program that incorporates pedometer use is effective for improving glucose tolerance in those with IGT. This result is likely to have important implications for future primary care– based diabetes prevention initiatives. |
| **Notes** | Paper:  <https://www.ncbi.nlm.nih.gov/pmc/articles/PMC2713638/>  Trial number:  Clinical trial reg. no. NCT00566319, [clinicaltrials.gov](http://clinicaltrials.gov/).  All authors has been contacted about table 2.  End point scores was calculated with baseline scores and change scores. SD from baseline were used as SD for end point. |

Risk of bias table

| **Bias** | **Authors' judgement** | **Support for judgement** |
| --- | --- | --- |
| Bias arising from the randomization process | Low risk | Question 1.1 Yes, the allocation sequence was random (flip of a coin)  Question 1.2 No information about concealment of allocation sequence  Question 1.3 There were no baseline imbalances to suggest a problem with the randomization. |
| Bias due to deviations from intended interventions | Unclear risk | Question 2.1 Participants were probably aware of intervention  Question 2.2 Personnel were probably aware of intervention  Question 2.3 No information  Question 2.4 Deviations did probably not affect outcome |
| Bias due to missing outcome data | Low risk | The reasons for missing outcome data are similar across groups |
| Bias in measurement of the outcome | Low risk | Question 4.2 The assessment were probably not likely to be influenced by knowledge on participant allocation. |
| Bias in selection of the reported result All outcomes | High risk | Judgement Comment: BMI was not reported and measured at follow up. The Clinical Trials register has BMI as a secondary outcome. |
| Overall bias All outcomes | Low risk | Judgement Comment: BMI should have been reported. However, for the outcome measures extracted, no bias seems present. |

| **Table S2**. Univariate meta-regressions between standardized mean differences from all outcomes and age, gender distribution, number of participants with walking aids, intervention length, baseline physical activity and body mass index. | | | | |
| --- | --- | --- | --- | --- |
| **Physical activity** | **Slope for one-unit change** | **95%CI** | **K** | ***Tau^2^*** |
| **Age** (years) | -0.037 | (-0.095 to 0.021) | 21 | 0.26 |
| **Gender distribution** (percent male) | -0.0064 | (-0.019 to 0.007) | 19 | 0.26 |
| **Walking aids** (percent users) | - | - | - | - |
| **Baseline physical activity** (1000 steps/day) | 0.061 | (-0.217 to 0.340) | 12 | 0.39 |
| **Intervention length** (weeks) | -0.012 | (-0.027 to 0.003) | 21 | 0.24 |
| **Body mass index** (kg/m^2^) | 0.006 | (-0.15 to 0.16) | 13 | 0.47 |
| Overall Tau^2^ value: 0.2648. Insufficient observations for ‘walking aids’. | | | | |
| **Moderate to vigorous physical activity** | **Slope for one-unit change** | **95%CI** | **K** | ***Tau^2^*** |
| **Age** (years) | 0.006 | (-0.055 to 0.067) | 8 | 0.074 |
| **Gender distribution** (percent male) | -0.011 | (-0.023 to 0.001) | 8 | 0.26 |
| **Walking aids** (percent users) | - | - | - | - |
| **Baseline physical activity** (1000 steps/day) | 0.100 | (-0.200 to 0.400) | 4 | 0.028 |
| **Intervention length** (weeks) | -0.001 | (-0.019 to 0.007) | 8 | 0.048 |
| **Body mass index** (kg/m^2^) | 0.0035 | (-0.394 to 0.401) | 6 | 0.127 |
| Overall Tau^2^ value: 0.052. Insufficient observations for ‘walking aids’. | | | | |
| **Physical capacity** | **Slope for one-unit change** | **95%CI** | **K** | ***Tau^2^*** |
| **Age** (years) | -0.103 | (-0.41 to 0.20) | 4 | 0.028 |
| **Gender distribution** (percent male) | -0.0093 | (-0.075 to 0.056) | 3 | 0.0 |
| **Walking aids** (percent users) | - | - | - | - |
| **Baseline physical activity** (1000 steps/day) | - | - | - | - |
| **Intervention length** (weeks) | -0.014 | (-0.052 to 0.024) | 4 | 0.022 |
| **Body mass index** (kg/m^2^) | - | . | - | - |
| Overall Tau^2^ value: 0.047. Insufficient observations for ‘walking aids’ and ‘Body mass index’ | | | | |
| **Body mass index** | **Slope for one-unit change** | **95%CI** | **K** | ***Tau^2^*** |
| **Age** (years) | -0.012 | (-0.29 to 0.27) | 3 | 0.0 |
| **Gender distribution** (percent male) | -0.006 | (-0.13 to 0.11) | 3 | 0.0 |
| **Walking aids** (percent users) | - | - | - | - |
| **Baseline physical activity** (steps/day) | - | - | - | - |
| **Intervention length** (weeks) | 0.15 | (-0.20 to 0.50) | 3 | 0.0 |
| **Body mass index** (kg/m^2^) | 0.05 | (-0.87 to 0.98) | 3 | 0.0 |
| Overall Tau^2^ value: 0.0 Insufficient observations for ‘walking aids’ and ‘baseline physical activity’. |  |  |  |  |
| **Health-related quality of life** | **Slope for one-unit change** | **95%CI** | **K** | ***Tau^2^*** |
| **Age** (years) | -0.019 | (-0.11 to 0.08) | 5 | 0.0 |
| **Gender distribution** (percent male) | -0.001 | (-0.016 to 0.013) | 5 | 0.0 |
| **Walking aids** (percent users) | - | - | - | - |
| **Baseline physical activity** (steps/day) | - | - | - | - |
| **Intervention length** (weeks) | -0.02 | (-0.01 to 0.009) | 5 | 0.0 |
| **Body mass index** (kg/m^2^) | 0.02 | (-0.85 to 0.86) | 3 | 0.0 |
| Overall Tau^2^ value: 0.0. K: number of included studies. Insufficient observations for ‘walking aids’ and ‘baseline physical activity’. |  |  |  |  |
| A p-value ≤ 0.05 is considered significant. Coef.: Coefficient, 95%CI: 95% confidence interval, K: Number of studies included in the meta-regression analysis, | | | | |

**Table S3.** Citations and reasons for exclusion from full text screening

| Daily self-monitoring of body weight, step count, fruit/vegetable intake, and water consumption: a feasible and effective long-term weight loss maintenance approach. | Akers JD.; Cornett RA.; Savla JS.; Davy KP.; Davy BM. | 2012 | Journal of the Academy of Nutrition and Dietetics | 10.1016/j.jand.2012.01.022 | Exclusion reason: Population mean age not above 65 years; |
| --- | --- | --- | --- | --- | --- |
| A randomized trial of a combined physical activity and environmental intervention in nursing home residents: do sleep and agitation improve? | Alessi CA.; Yoon EJ.; Schnelle JF.; Al-Samarrai NR.; Cruise PA. | 1999 | Journal of the American Geriatrics Society |  | Exclusion reason: Not PAM versus other; |
| Continuous glucose monitoring counseling improves physical activity behaviors of individuals with type 2 diabetes: A randomized clinical trial. | Allen NA.; Fain JA.; Braun B.; Chipkin SR. | 2008 | Diabetes research and clinical practice | 10.1016/j.diabres.2008.01.006 | Exclusion reason: Population mean age not above 65 years; |
| Behavioural intervention to increase physical activity in adults with coronary heart disease in Jordan. | Alsaleh E.; Windle R.; Blake H. | 2016 | BMC public health | 10.1186/s12889-016-3313-5 | Exclusion reason: Population mean age not above 65 years; |
| Efficacy of a pedometer-based physical activity program on parameters of diabetes control in type 2 diabetes mellitus. | Araiza P.; Hewes H.; Gashetewa C.; Vella CA.; Burge MR. | 2006 | Metabolism: clinical and experimental | 10.1016/j.metabol.2006.06.009 | Exclusion reason: Population mean age not above 65 years; |
| The effect of a pedometer-based community walking intervention "Walking for Wellbeing in the West" on physical activity levels and health outcomes: a 12-week randomized controlled trial | Baker, Graham; Gray, Stuart R.; Wright, Annemarie; Fitzsimons, Claire; Nimmo, Myra; Lowry, Ruth; Mutrie, Nanette | 2008 | International Journal of Behavioral Nutrition and Physical Activity | 10.1186/1479-5868-5-44 | Exclusion reason: Population mean age not above 65 years; |
| Home-based telerehabilitation in older patients with chronic obstructive pulmonary disease and heart failure: a randomised controlled trial | Bernocchi, P; Vitacca, M; Rovere, Mt; Volterrani, M; Galli, T; Baratti, D; Paneroni, M; Campolongo, G; Sposato, B; Scalvini, S | 2017 | Age and ageing | 10.1093/ageing/afx146 | Exclusion reason: Not PAM versus other; |
| Automated interventions for multiple health behaviors using conversational agents. | Bickmore TW.; Schulman D.; Sidner C. | 2013 | Patient education and counseling | 10.1016/j.pec.2013.05.011 | Exclusion reason: Population mean age not above 65 years; |
| A randomized controlled trial of an automated exercise coach for older adults. | Bickmore TW.; Silliman RA.; Nelson K.; Cheng DM.; Winter M.; Henault L.; Paasche-Orlow MK. | 2013 | Journal of the American Geriatrics Society | 10.1111/jgs.12449 | Exclusion reason: Not PAM versus other; |
| Regular use of pedometer does not enhance beneficial outcomes in a physical activity intervention study in type 2 diabetes mellitus. | BjÃ¸rgaas MR.; Vik JT.; StÃ¸len T.; Lydersen S.; Grill V. | 2008 | Metabolism: clinical and experimental | 10.1016/j.metabol.2007.12.002 | Exclusion reason: Population mean age not above 65 years; |
| An Internet-Based Physical Activity Intervention to Improve Quality of Life of Inactive Older Adults: A Randomized Controlled Trial. | Broekhuizen K.; de Gelder J.; Wijsman CA.; Wijsman LW.; Westendorp RG.; Verhagen E.; Slagboom PE.; de Craen AJ.; van Mechelen W.; van Heemst D.; van der Ouderaa F.; Mooijaart SP. | 2016 | Journal of medical Internet research | 10.2196/jmir.4335 | Exclusion reason: Population mean age not above 65 years; |
| Objectively-measured physical activity and incident cardiovascular events among mobility limited older adults: The life study | Buford T.W.; Cochrane S.; Fitzgerald J.; Chen S.-H.; Dodson J.; Fielding R.; King A.; Manini T.; Marsh A.; McDermott M.; Newman A.; Pahor M.; Tudor-Locke C.; Ambrosius W. | 2016 | Circulation |  | Exclusion reason: Not PAM versus other; |
| Peer Volunteers Improve Long-Term Maintenance of Physical Activity With Older Adults: A Randomized Controlled Trial. | Buman MP.; Giacobbi PR.; Dzierzewski JM.; Morgan AA.; McCrae CS.; Roberts BL.; Marsiske M. | 2011 | Journal of physical activity & health | 10.1123/jpah.8.s2.s257 | Exclusion reason: Not PAM versus other; |
| Effects of a pedometer-based intervention on physical activity levels after cardiac rehabilitation: a randomized controlled trial. | Butler L.; Furber S.; Phongsavan P.; Mark A.; Bauman A. | | Journal of cardiopulmonary rehabilitation and prevention | 10.1097/HCR.0b013e31819a01ff | Exclusion reason: Population mean age not above 65 years; |
| Impact of APOE e4 on the association of physical activity and cognition in older adults with memory complaints | Cox K.; Flicker L.; Almeida O.; Van Bockxmeer F.; Greenop K.; Hendriks J.; Phillips M.; Lautenschlager N. | 2012 | Journal of Science and Medicine in Sport | http://dx.doi.org/10.1016/j.jsams.2012.11.060 | Exclusion reason: Not RCT/RCO |
| The FABS trial: a randomised control trial of the effects of a 6-month physical activity intervention on adherence and long-term physical activity and self-efficacy in older adults with memory complaints. | Cox KL.; Flicker L.; Almeida OP.; Xiao J.; Greenop KR.; Hendriks J.; Phillips M.; Lautenschlager NT. | 2013 | Preventive medicine | 10.1016/j.ypmed.2013.09.010 | Exclusion reason: Not PAM versus other; |
| The predictors of short- and long-term physical activity levels of older adults from fabs (Fitness for the aging brain study) | Cox, K; Flicker, L; Almeida, O; Greenop, Kk; Hendricks, J; Phillips, M; Lautenschlager, Nt | 2012 | Alzheimer's & dementia | 10.1016/j.jalz.2012.05.200 | Exclusion reason: Not PAM versus other; |
| Combining Fast-Walking Training and a Step Activity Monitoring Program to Improve Daily Walking Activity After Stroke: A Preliminary Study. | Danks KA.; Pohlig R.; Reisman DS. | 2016 | Archives of physical medicine and rehabilitation | 10.1016/j.apmr.2016.01.039 | Exclusion reason: Population mean age not above 65 years; |
| An intervention to promote walking amongst the general population based on an 'extended' theory of planned behaviour: A waiting list randomised controlled trial | Darker C.D.; French D.P.; Eves F.F.; Sniehotta F.F. | 2010 | Psychology and Health | http://dx.doi.org/10.1080/08870440902893716 | Exclusion reason: Population mean age not above 65 years; |
| Preventing diabetes in primary care: a feasibility cluster randomized trial. | Dawes D.; Ashe M.; Campbell K.; Cave D.; Elley CR.; Kaczorowski J.; Sohal P.; Ur E.; Dawes M. | 2015 | Canadian journal of diabetes | 10.1016/j.jcjd.2014.08.004 | Exclusion reason: Not PAM versus other; |
| The effects of a lifestyle physical activity counseling program with feedback of a pedometer during pulmonary rehabilitation in patients with COPD: a pilot study. | de Blok BM.; de Greef MH.; ten Hacken NH.; Sprenger SR.; Postema K.; Wempe JB. | 2006 | Patient education and counseling | 10.1016/j.pec.2005.02.005 | Exclusion reason: Population mean age not above 65 years; |
| A cognitive-behavioural pedometer-based group intervention on physical activity and sedentary behaviour in individuals with type 2 diabetes. | De Greef K.; Deforche B.; Tudor-Locke C.; De Bourdeaudhuij I. | 2010 | Health education research | 10.1093/her/cyq017 | Exclusion reason: Population mean age not above 65 years; |
| Exercise to enhance mobility and prevent falls after stroke: the community stroke club randomized trial. | Dean CM.; Rissel C.; Sherrington C.; Sharkey M.; Cumming RG.; Lord SR.; Barker RN.; Kirkham C.; O'Rourke S. | | Neurorehabilitation and neural repair | 10.1177/1545968312441711 | Exclusion reason: Not PAM versus other; |
| Promoting physical activity for persons with diabetes. | Diedrich A.; Munroe DJ.; Romano M. | | The Diabetes educator | 10.1177/0145721709352382 | Exclusion reason: Population mean age not above 65 years; |
| Dose of physical activity, physical functioning and disability risk in mobility-limited older adults: Results from the LIFE study randomized trial. | Fielding RA.; Guralnik JM.; King AC.; Pahor M.; McDermott MM.; Tudor-Locke C.; Manini TM.; Glynn NW.; Marsh AP.; Axtell RS.; Hsu FC.; Rejeski WJ.; . | 2017 | PloS one | 10.1371/journal.pone.0182155 | Exclusion reason: Not PAM versus other; |
| Efficacy of quantified home-based exercise and supervised exercise in patients with intermittent claudication: a randomized controlled trial. | Gardner AW.; Parker DE.; Montgomery PS.; Scott KJ.; Blevins SM. | 2011 | Circulation | 10.1161/CIRCULATIONAHA.110.963066 | Exclusion reason: Not PAM versus other; |
| Adverse events among high-risk participants in a home-based walking study: A descriptive study | Goodrich D.E.; Larkin A.R.; Lowery J.C.; Holleman R.G.; Richardson C.R. | 2007 | International Journal of Behavioral Nutrition and Physical Activity | http://dx.doi.org/10.1186/1479-5868-4-20 | Exclusion reason: Population mean age not above 65 years; |
| Effect of a Primary Care Walking Intervention with and without Nurse Support on Physical Activity Levels in 45- to 75-Year-Olds: The Pedometer And Consultation Evaluation (PACE-UP) Cluster Randomised Clinical Trial. | Harris T.; Kerry SM.; Limb ES.; Victor CR.; Iliffe S.; Ussher M.; Whincup PH.; Ekelund U.; Fox-Rushby J.; Furness C.; Anokye N.; Ibison J.; DeWilde S.; David L.; Howard E.; Dale R.; Smith J.; Cook DG. | 2017 | PLoS medicine | 10.1371/journal.pmed.1002210 | Exclusion reason: Population mean age not above 65 years; |
| Evaluation of a Shelter-Based Diet and Physical Activity Intervention for Homeless Adults. | Kendzor, Darla E.; Allicock, Marlyn; Businelle, Michael S.; Sandon, Lona F.; Gabriel, Kelley Pettee; Frank, Summer G. | 2017 | Journal of Physical Activity & Health |  | Exclusion reason: Population mean age not above 65 years; |
| Sit to stand activity during stroke rehabilitation. | Kerr A.; Dawson J.; Robertson C.; Rowe P.; Quinn TJ. | 2017 | Topics in stroke rehabilitation | 10.1080/10749357.2017.1374687 | Exclusion reason: Wrong outcomes; |
| Cluster randomized controlled trial of a multilevel physical activity intervention for older adults. | Kerr J.; Rosenberg D.; Millstein RA.; Bolling K.; Crist K.; Takemoto M.; Godbole S.; Moran K.; Natarajan L.; Castro-Sweet C.; Buchner D. | 2018 | The international journal of behavioral nutrition and physical activity | 10.1186/s12966-018-0658-4 | Exclusion reason: Not RCT/RCO; |
| Promoting physical activity through hand-held computer technology. | King AC.; Ahn DK.; Oliveira BM.; Atienza AA.; Castro CM.; Gardner CD. | 2008 | American journal of preventive medicine | 10.1016/j.amepre.2007.09.025 | Exclusion reason: Population mean age not above 65 years; |
| Healthy steps trial: effectiveness of a pedometer-based green prescription for low-active older adults in primary care | Kolt, G; Schofield, Gm; Kerse, N; Garrett, N; Ashton, T | 2011 | Physiotherapy (united kingdom). | 10.1016/j.physio.2011.04.002 | Exclusion reason: Duplicate; |
| A randomized exercise trial in older women: increased activity over two years and the factors associated with compliance. | Kriska AM.; Bayles C.; Cauley JA.; LaPorte RE.; Sandler RB.; Pambianco G. | 1986 | Medicine and science in sports and exercise |  | Exclusion reason: Full text unavailable; |
| The feasibility and RE-AIM evaluation of the TAME health pilot study | Lewis Z.H.; Ottenbacher K.J.; Fisher S.R.; Jennings K.; Brown A.F.; Swartz M.C.; Martinez E.; Lyons E.J. | 2017 | International Journal of Behavioral Nutrition and Physical Activity | http://dx.doi.org/10.1186/s12966-017-0560-5 | Exclusion reason: Population mean age not above 65 years; |
| Impact of increasing physical activity over 12 weeks in previously sedentary older adults: a 1-year follow up | Malik, Ma; Suboc, T; Strath, Sj; Wang, J; Tanner, Mj; Ying, R; Widlansky, M | 2015 | Journal of the american college of cardiology. |  | Exclusion reason: Population mean age not above 65 years; |
| Use of Accelerometer-Based Feedback of Walking Activity for Appraising Progress With Walking-Related Goals in Inpatient Stroke Rehabilitation: a Randomized Controlled Trial | Mansfield, A; Wong, Js; Bryce, J; Brunton, K; Inness, El; Knorr, S; Jones, S; Taati, B; McIlroy, We | 2015 | Neurorehabilitation and neural repair | 10.1177/1545968314567968 | Exclusion reason: Population mean age not above 65 years; |
| Should all steps count when using a pedometer as a measure of physical activity in older adults? | Marshall AL. | 2007 | Journal of physical activity & health |  | Exclusion reason: Population mean age not above 65 years; |
| Pedometer-facilitated walking intervention shows promising effectiveness for reducing cancer fatigue: a pilot randomized trial. | Mayo NE.; Moriello C.; Scott SC.; Dawes D.; Auais M.; Chasen M. | 2014 | Clinical rehabilitation | 10.1177/0269215514536209 | Exclusion reason: Population mean age not above 65 years; |
| Assessing the Effects of Interpersonal and Intrapersonal Behavior Change Strategies on Physical Activity in Older Adults: a Factorial Experiment. | McMahon SK.; Lewis B.; Oakes JM.; Wyman JF.; Guan W.; Rothman AJ. | 2017 | Annals of behavioral medicine : a publication of the Society of Behavioral Medicine | 10.1007/s12160-016-9863-z | Exclusion reason: Not PAM versus other; |
| Increasing walking among older people: a test of behaviour change techniques using factorial randomised N-of-1 trials | Nyman, Sr; Goodwin, K; Kwasnicka, D; Callaway, A | 2016 | Psychology & health | 10.1080/08870446.2015.1088014 | Exclusion reason: Not PAM versus other; |
| A randomised controlled trial to investigate walking 6,000 steps per day on pain and function in knee osteoarthritis progression: the walkout study | O'Hanlon, Ms; Siddiqi, Q; Allen, Sl; Edwards, Kl | 2016 | Osteoarthritis and cartilage. |  | Exclusion reason: Full text unavailable |
| Efficacy of a computer-based intervention to promote walking in older adults | Paasche-Orlow M.; Silliman R.; Winter M.; Cheng D.; Henault L.; Bickmore T. | 2012 | Journal of the American Geriatrics Society | http://dx.doi.org/10.1111/j.1532-5415.2012.04000.x | Exclusion reason: Full text unavailable; |
| Effects of the DASH Diet and Walking on Blood Pressure in Patients With Type 2 Diabetes and Uncontrolled Hypertension: A Randomized Controlled Trial. | Paula TP.; Viana LV.; Neto AT.; LeitÃ£o CB.; Gross JL.; Azevedo MJ. | 2015 | Journal of clinical hypertension (Greenwich, Conn.) | 10.1111/jch.12597 | Exclusion reason: Population mean age not above 65 years; |
| Efficacy of multiparametric telemonitoring on respiratory outcomes in elderly people with COPD: a randomized controlled trial. | Pedone C.; Chiurco D.; Scarlata S.; Incalzi RA. | 2013 | BMC health services research | 10.1186/1472-6963-13-82 | Exclusion reason: Wrong outcomes; |
| A population-based randomized controlled trial of the effect of combining a pedometer with an intervention toolkit on physical activity among individuals with low levels of physical activity or fitness. | Petersen CB.; Severin M.; Hansen AW.; Curtis T.; GrÃ¸nbÃ¦k M.; Tolstrup JS. | 2012 | Preventive medicine | 10.1016/j.ypmed.2011.12.012 | Exclusion reason: Population mean age not above 65 years; |
| Effects of a pedometer-based intervention on the physical performance and mobility-related self-efficacy of community-dwelling older adults: an interdisciplinary preventive health care intervention. | Richeson NE; Croteau KA; Jones DB; Farmer BC | | Therapeutic Recreation Journal |  | Exclusion reason: Wrong outcomes; |
| Physical activity promotion in the primary care setting in pre- and type 2 diabetes - the Sophia step study, an RCT. | Rossen J.; Yngve A.; HagstrÃ¶mer M.; Brismar K.; Ainsworth BE.; Iskull C.; MÃ¶ller P.; Johansson UB. | 2015 | BMC public health | 10.1186/s12889-015-1941-9 | Exclusion reason: Not RCT/RCO; |
| A randomized trial to increase physical activity among native elders. | Sawchuk CN.; Charles S.; Wen Y.; Goldberg J.; Forquera R.; Roy-Byrne P.; Buchwald D. | 2008 | Preventive medicine | 10.1016/j.ypmed.2008.03.011 | Exclusion reason: Population mean age not above 65 years; |
| Does the effect of walking intervention wear off during ramadhan in older malays with type 2 diabetes? | Sazlina, S-G; Browning, C; Yasin, S | 2013 | Diabetes technology and therapeutics. | 10.1089/dia.2012.1221 | Exclusion reason: Full text unavailable; |
| Effect of a Randomized Controlled Trial Walking Program on Walking, Stress, Depressive Symptoms and Cardiovascular Biomarkers in Elderly Korean Immigrants | Sin, Mk; Ibarra, B; Tae, T; Murphy, Pj | 2015 | J korean biol nurs sci | 10.7586/jkbns.2015.17.2.89 | Exclusion reason: Wrong outcomes; |
| Daily physical activity in COPD: Quantification by tri-axial accelerometry | Sirichana W.; Moore-Gillon C.E.; Patel M.H.; Taylor M.; Storer T.W.; Cooper C.B. | 2013 | American Journal of Respiratory and Critical Care Medicine |  | Exclusion reason: Not RCT/RCO; |
| Effects of lifestyle modifications on patients with type 2 diabetes: the Japan Diabetes Complications Study (JDCS) study design, baseline analysis and three year-interim report. | Sone H.; Katagiri A.; Ishibashi S.; Abe R.; Saito Y.; Murase T.; Yamashita H.; Yajima Y.; Ito H.; Ohashi Y.; Akanuma Y.; Yamada N.; . | 2002 | Hormone and metabolic research = Hormon- und Stoffwechselforschung = Hormones et metabolisme | 10.1055/s-2002-34791 | Exclusion reason: Population mean age not above 65 years; |
| A Randomized Clinical Trial of an Activity and Exercise Adherence Intervention in Chronic Pulmonary Disease | Steele B.G.; Belza B.; Cain K.C.; Coppersmith J.; Lakshminarayan S.; Howard J.; Haselkorn J.K. | 2008 | Archives of Physical Medicine and Rehabilitation | http://dx.doi.org/10.1016/j.apmr.2007.11.003 | Exclusion reason: Not PAM versus other; |
| The impact of moderate intensity physical activity on cardiac structure and performance in older sedentary adults | Suboc T.B.; Strath S.J.; Dharmashankar K.; Harmann L.; Couillard A.; Malik M.; Haak K.; Knabel D.; Widlansky M.E. | 2014 | IJC Heart and Vessels | http://dx.doi.org/10.1016/j.ijchv.2014.08.007 | Exclusion reason: Population mean age not above 65 years; |
| Relative importance of step count, intensity, and duration on physical activity's impact on vascular structure and function in previously sedentary older adults. | Suboc TB.; Strath SJ.; Dharmashankar K.; Coulliard A.; Miller N.; Wang J.; Tanner MJ.; Widlansky ME. | 2014 | Journal of the American Heart Association | 10.1161/JAHA.113.000702 | Exclusion reason: Population mean age not above 65 years; |
| A telehealth program for self-management of COPD exacerbations and promotion of an active lifestyle: a pilot randomized controlled trial. | Tabak M.; Brusse-Keizer M.; van der Valk P.; Hermens H.; Vollenbroek-Hutten M. | 2014 | International journal of chronic obstructive pulmonary disease | 10.2147/COPD.S60179 | Exclusion reason: Population mean age not above 65 years; |
| Randomized controlled trial on cardiovascular risk management by practice nurses supported by self-monitoring in primary care. | Tiessen AH.; Smit AJ.; Broer J.; Groenier KH.; van der Meer K. | 2012 | BMC family practice | 10.1186/1471-2296-13-90 | Exclusion reason: Optional PAM Intervention in the intervention group; |
| Effects of exercise intervention in breast cancer patients: is mobile health (mHealth) with pedometer more effective than conventional program using brochure? | Uhm K.E.; Yoo J.S.; Chung S.H.; Lee J.D.; Lee I.; Kim J.I.; Lee S.K.; Nam S.J.; Park Y.H.; Lee J.Y.; Hwang J.H. | 2017 | Breast Cancer Research and Treatment | http://dx.doi.org/10.1007/s10549-016-4065-8 | Exclusion reason: Population mean age not above 65 years; |
| Maintenance of physical activity in breast cancer survivors after a randomized trial. | Vallance JK.; Courneya KS.; Plotnikoff RC.; Dinu I.; Mackey JR. | 2008 | Medicine and science in sports and exercise | 10.1249/mss.0b013e3181586b41 | Exclusion reason: Population mean age not above 65 years; |
| Analyzing theoretical mechanisms of physical activity behavior change in breast cancer survivors: results from the activity promotion (ACTION) trial. | Vallance JK.; Courneya KS.; Plotnikoff RC.; Mackey JR. | 2008 | Annals of behavioral medicine : a publication of the Society of Behavioral Medicine | 10.1007/s12160-008-9019-x | Exclusion reason: Population mean age not above 65 years; |
| Randomized controlled trial of the effects of print materials and step pedometers on physical activity and quality of life in breast cancer survivors. | Vallance JK.; Courneya KS.; Plotnikoff RC.; Yasui Y.; Mackey JR. | 2007 | Journal of clinical oncology : official journal of the American Society of Clinical Oncology | 10.1200/JCO.2006.07.9988 | Exclusion reason: Population mean age not above 65 years; |
| The Impact of Different Degrees of Feedback on Physical Activity Levels: A 4-Week Intervention Study. | Van Hoye K.; Boen F.; Lefevre J. | 2015 | International journal of environmental research and public health | 10.3390/ijerph120606561 | Exclusion reason: Population mean age not above 65 years; |
| Effects of a web-based intervention on physical activity and metabolism in older adults: randomized controlled trial. | Wijsman CA.; Westendorp RG.; Verhagen EA.; Catt M.; Slagboom PE.; de Craen AJ.; Broekhuizen K.; van Mechelen W.; van Heemst D.; van der Ouderaa F.; Mooijaart SP. | 2013 | Journal of medical Internet research | 10.2196/jmir.2843 | Exclusion reason: Population mean age not above 65 years; |
| Effects of a web-based intervention on physical activity and metabolism in older adults: randomized controlled trial | Wijsman, Ca; Westendorp, Rgj; Verhagen, Ealm; Catt, M; Slagboom, Pe; Craen, Ajm; Broekhuizen, K; Mechelen, W; Heemst, D; Ouderaa, F; Mooijaart, Sp | 2015 | Diabetes technology & therapeutics | 10.1089/dia.2015.1507 | Exclusion reason: Population mean age not above 65 years; |
| Pedometer-Based Behavioral Change Program Can Improve Dependency in Sedentary Older Adults: A Randomized Controlled Trial. | Yamada M.; Mori S.; Nishiguchi S.; Kajiwara Y.; Yoshimura K.; Sonoda T.; Nagai K.; Arai H.; Aoyama T. | 2012 | The Journal of frailty & aging | 10.14283/jfa.2012.7 | Exclusion reason: Full text unavailable; |
| The Pre-diabetes Risk Education and Physical Activity Recommendation and Encouragement (PREPARE) programme study: are improvements in glucose regulation sustained at 2 years? | Yates T.; Davies MJ.; Sehmi S.; Gorely T.; Khunti K. | 2011 | Diabetic medicine : a journal of the British Diabetic Association | 10.1111/j.1464-5491.2011.03357.x | Exclusion reason: Wrong outcomes; |
| Walking Away from Type 2 diabetes: a cluster randomized controlled trial. | Yates T.; Edwardson CL.; Henson J.; Gray LJ.; Ashra NB.; Troughton J.; Khunti K.; Davies MJ. | 2017 | Diabetic medicine : a journal of the British Diabetic Association | 10.1111/dme.13254 | Exclusion reason: Population mean age not above 65 years; |
| Effectiveness of long-term intervention in physical activity of type 2 diabetics (prospective randomized controlled group study for 1 year using a unique pedometer) | Yokochi M.; Niinomi M.; Kato Y.; Yanbe Y.; Tsushita K.; Ohiso Y. | 2002 | Journal of the Japan Diabetes Society |  | Exclusion reason: Population mean age not above 65 years; |
| Efficacy of a gender-tailored intervention to prevent weight regain in men over 3 years: A weight loss maintenance RCT | Young M.D.; Callister R.; Collins C.E.; Plotnikoff R.C.; Aguiar E.J.; Morgan P.J. | 2017 | Obesity | http://dx.doi.org/10.1002/oby.21696 | Exclusion reason: Population mean age not above 65 years; |
