# Supplementary material for: Physical activity monitors to enhance amount of physical activity in older adults – a systematic review and meta-analysis
Source: Eur Rev Aging Phys Act. 2019 May 4;16:7. doi: 10.1186/s11556-019-0213-6 (PMC6500067; doi:10.1186/s11556-019-0213-6)
Supplement: Supplementary file 2 — Summary of findings table. (DOCX 17 kb) [file 11556_2019_213_MOESM2_ESM.docx]

**Summary of findings table**

| **Physical activity monitor interventions compared to control interventions in older adults** | | | | | | |
| --- | --- | --- | --- | --- | --- | --- |
| Outcomes | **Anticipated absolute effects^*^**  (95% CI) | | **Standardized mean difference**  (95% CI) | **№ of studies**  (participants) | **Certainty of the evidence** (GRADE) | **Comments** |
| **Physical activity** - objectively measured or self-reported | The translated weighted mean difference was hence 1,297 steps per day (95% CI: 817 to 1,753) with more steps in the intervention groups | | SMD 0.53  (0.34 to 0.73) in favor of the intervention | 20 studies (21 study comparisons and 2,704 participants) | ⊕⊕⭘⭘^1,2^  Low | None |
| **Time spent sedentary** | The mean difference of weekly sedentary time was 44 minutes (37.1 to 50.9) with the control group being more sedentary. | | SMD 0.40 (-0.27 to 1.07) in favor of the intervention | One study with 39 participants | ⊕⭘⭘⭘^4^  Very low | None |
| **Moderate to vigorous physical activity** - objectively measured or self-reported | The translated weighted mean difference was hence 5.5 minutes per day (95% CI: 2.4 to 8.4) with more MVPA in the intervention groups | | SMD 0.34  (0.15 to 0.52) in favor of the intervention | 8 studies (1,686 participants) | ⊕⊕⊕⭘^1^  Moderate | None |
| **Physical capacity** measured with walking tests | The translated weighted mean difference was hence 15 meters (95% CI: -8 to 38) with more meters walked on a 6MWT in the intervention groups. | | SMD 0.19  (-0.10 to 0.48) in favor of the intervention | 4 studies (754 participants) | ⊕⊕⭘⭘^1,3^  Low | None |
| **Body mass index** | The translated weighted mean difference was hence 0.72 kg/m^2^ (95% CI: -0.048 to 1.5) with the control group having the lowest BMI. | | SMD 0.15  (-0.01 to 0.31) in favor of the control intervention | 3 studies (570 participants) | ⊕⊕⊕⭘^1^  Moderate | None |
| **Self-reported HRQoL** – assessed with questionnaires | No mean value available as the studies used different outcome measures. | | SMD 0.01  (-0.12 to 0.14) in favor of the intervention | 5 studies (1,038 participants) | ⊕⊕⭘⭘^1,3^  Low | None |
| **Meeting the study specific recommended level of physical activity’** | No data | | No data | No data | No data | No data |
| **Adverse events and withdrawals** | Calculated with 64 withdrawals of 881 participants in control groups | Calculated with 63 withdrawals of 1,149 participants in intervention groups | Relative risk for withdrawal due to illness or adverse events 0.91 (0.66 to 1.25) with higher risk in control group | 11 studies (1,927 participants) | ⊕⊕⊕⭘^3^  Moderate | None |

*The absolute effects are calculated from the standardized mean differences and a relevant standard deviation according to the method section.
**HRQoL**: Health related quality of life
**CI:** Confidence interval; **SMD:** Standardized mean difference

^1^: Downgraded by one level due to inconsistency (unexplained heterogeneity)

^2^: Downgraded by one level due to publication bias

^3^: Downgraded by one level due to imprecision of the results

^4^: Sparse data

**GRADE Working Group grades of evidence**
**High certainty:** We are very confident that the true effect lies close to that of the estimate of the effect
**Moderate certainty:** We are moderately confident in the effect estimate: The true effect is likely to be close to the estimate of the effect, but there is a possibility that it is substantially different
**Low certainty:** Our confidence in the effect estimate is limited: The true effect may be substantially different from the estimate of the effect
**Very low certainty:** We have very little confidence in the effect estimate: The true effect is likely to be substantially different from the estimate of effect
